# Expanding Iron Acquisition in Maize: Root Sector-Specific Responses and Gibberellin Regulation of Ferric and Ferrous Iron Uptake

**DOI:** 10.3390/ijms27031323

**Published:** 2026-01-28

**Authors:** Yannis E. Ventouris, Idyli Elissavet Charatsidou, Kimon Ionas, Georgios P. Stylianidis, Chrysoula K. Pantazopoulou, Dimitris L. Bouranis, Styliani N. Chorianopoulou

**Affiliations:** Plant Physiology and Morphology Laboratory, Crop Science Department, Agricultural University of Athens, 11855 Athens, Greece; yannisventouris@gmail.com (Y.E.V.); idylicharatsidou@aua.gr (I.E.C.); kimonionas@gmail.com (K.I.); g.stylianidis@aua.gr (G.P.S.); c.pantazopoulou@aua.gr (C.K.P.); bouranis@aua.gr (D.L.B.)

**Keywords:** gibberellins, iron homeostasis, maize, root phenotype, strategy I, strategy II, *Zea mays*

## Abstract

Iron (Fe) is an essential micronutrient for plant development and productivity. Nevertheless, the role of gibberellins (GAs) in the control of iron homeostasis is less studied compared to other growth regulators. We found that GAs modulate iron homeostasis in maize by inducing deficiency-like responses independent of rhizosphere iron availability. Plant phenotyping demonstrated that exogenous GA_3_ application under iron-sufficient conditions phenocopied iron deprivation, while inhibiting GA biosynthesis with mepiquat chloride prevented the development of typical symptoms of Fe deficiency (–Fe). Gibberellins positively control strategy II Fe uptake genes, albeit indirectly, as opposed to the direct negative transcriptional regulation of phytosiderophore biosynthesis. Additionally, gibberellins disrupt iron partitioning by suppressing root-to-shoot Fe translocation, causing iron overaccumulation in roots of GA_3_ treated plants. A functional ferrous iron uptake pathway was identified and was found to operate in conjunction with the strategy II uptake pathway via the differentially regulated *Zea mays* Iron-Regulated Transporter (IRT) paralogs *ZmIRT1* and *ZmIRT2*. Root responses are spatially organized: gene expression in the lateral root sector reflects the shoot iron status, while transcriptional responses in the root apex correlate with local Fe demands. This study demonstrates that maize leverages a hybrid ferric/ferrous iron uptake strategy and establishes novel roles of GAs as pivotal regulators of iron homeostasis.

## 1. Introduction

Iron (Fe) is an essential micronutrient for plant development and productivity, primarily due to its involvement in redox reactions, chlorophyll biosynthesis, and as a cofactor in key enzymes of photosynthesis and respiration [1,2]. Among the various micronutrients, iron is typically required by plants in the highest quantities [3]. Despite its abundance in soils, Fe bioavailability is often limited under aerobic and alkaline conditions due to its propensity to form insoluble hydroxides and oxides [4].

Based on the redox state of the iron acquired (Fe^2+^ or Fe^3+^) and the set of mechanisms employed for Fe uptake, plants have been traditionally categorized as strategy I or strategy II species. Strategy I (or reduction strategy), typical of dicots and non-graminaceous monocots, involves rhizosphere acidification, the secretion of Fe^3+^ chelating coumarins, the subsequent reduction of Fe^3+^ to Fe^2+^ by ferric-chelate reductases (FROs), and ultimately the uptake of ferrous iron via an IRT transporter [5,6]. In contrast, members of the Poaceae family utilize strategy II (or chelation strategy), which relies on the synthesis and secretion of mugineic acid family phytosiderophores (PSs) for the chelation of Fe^3+^, while a yellow stripe1 (YS1)/YS1-like (YSL) transporter facilitates the uptake of the Fe^3+^-PS complex [7]. Although traditionally viewed as mutually exclusive, growing evidence indicates that, beyond rice, which is long known to leverage a combined strategy using ferrous iron as a primary Fe source, other strategy II plants retain functional strategy I components as well. For example, in maize, both IRT homologues, i.e., ZmIRT1 and ZmIRT2, are Fe-responsive and have been demonstrated to possess Fe transporting activity [8,9,10]. Taken together, these observations support the Ancient Combined Strategy model, which proposes that strategy I and strategy II elements coexisted in the common ancestor of flowering plants, and consequently grasses likely continue to utilize strategy I components alongside their canonical strategy II machinery [11].

Beyond uptake, the distinction between the two strategies is also becoming increasingly blurred at the regulatory level. In *Arabidopsis*, the bHLH transcription factor FIT forms heterodimers with subgroup Ib bHLHs to activate Fe uptake genes like IRT1 and FRO2 [12]. Similarly in rice, and more recently in maize, FIT homologs have been shown to interact with IRO2 proteins, the grass homologs of the Ib bHLH transcription factors, to regulate Fe uptake genes [13].

The role of gibberellins (GAs) in the control of iron homeostasis is markedly less studied compared to other hormones and growth regulators such as auxin, ethylene, abscisic acid, NO, and even brassinosteroids. In *Arabidopsis*, GA promotes the degradation of DELLA proteins that otherwise inhibit FIT function, thereby enhancing Fe uptake [14]. In rice, however, exogenous GA exacerbates Fe-deficiency chlorosis, suppresses root-to-shoot Fe translocation, and reduces shoot iron content regardless of Fe availability. Analogously, in *Oseui1* mutants, the increase in endogenous GAs due to impaired GA deactivation led to suppressed Fe translocation and lower shoot Fe, whereas the GA biosynthesis inhibitor paclobutrazol restored Fe homeostasis and reduced deficiency symptoms. The opposite effect was observed in *OsEUI1* overexpressors [15,16]. Iron deficiency has been found to decrease the levels of endogenous bioactive GAs in rice and maize shoots [15,17], as well as in Arabidopsis [14], primarily in roots [18]. The roots of maize seedlings treated with exogenous GA_3_ develop the BTR (Branching at the Terminal 5 cm of the Root) phenotype [19], a phenotype normally developing under Fe deficiency [20], in which lateral roots ectopically emerge in closer proximity to the root tip. A close connection between gibberellins and the BTR phenotype has been described [19].

Despite these insights, key questions remain. For example, ZmIRT2 was only recently characterized [10], and although ZmIRT1 is able to transport Fe^2+^ [21,22], it is not clear to what extent these strategy I components contribute to Fe homeostasis in maize. Moreover, although gibberellins have been demonstrated to positively regulate strategy I in *Arabidopsis* at the molecular level [14,18], how gibberellins modulate the expression of strategy II genes or that of strategy I components in grasses remains unknown. Similarly, whether GAs directly control key iron homeostasis regulators needs to be elucidated. Importantly, as most investigations focusing on –Fe responses in the root system do not distinguish between different root parts, not much is known regarding the responses of different root sectors to iron deprivation. On the other hand, the majority of studies describing iron deficiency responses at the tissue level (i.e., root epidermis, cortex, stele, or vascular tissues) do so without referring to particular phytomeres (i.e., the root apex, the root sector baring lateral roots, etc.), even though the roles and hence the physiology of different phytomeres may vary substantially. In the present translational study, we attempted to address these open research questions with implications for agricultural practice, as well as for the quest to improve crop iron (Fe) use efficiency (FeUE).

## 2. Results

### 2.1. Plant Phenotype

#### 2.1.1. Plant Growth and Chlorophyll Content

**Figure 1:**
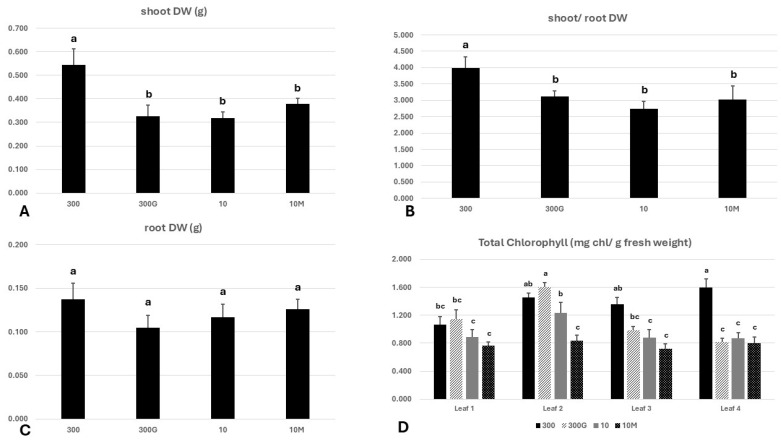
Shoot dry weight (g) per plant (**A**), shoot/root dry weight ratio (**B**), whole root system dry weight (g) per plant (**C**), and total chlorophyll content for each of the four leaves present, from the older (leaf 1) to the younger (leaf 4) (**D**), among the four treatments (300, 10, 300G, 10M) on day 7. 300: maize seedlings grown in iron-sufficient conditions (300 μM Fe). 300G: maize seedlings grown under iron sufficiency (300 μM Fe) in the presence of exogenous gibberellic acid (G). 10: maize seedlings grown in iron-insufficient conditions (10 μM Fe). 10M: maize seedlings grown under iron insufficiency (10 μM Fe) but with the addition of the gibberellin biosynthesis inhibitor mepiquat chloride (M) in the growth medium. Mean ± SE. Different letters denote significant differences (Tukey’s multiple comparison test *p* < 0.005).

The shoots of plants grown under iron sufficiency (treatment “300”) had accumulated a markedly higher amount of dry mass on day 7 (0.545 g per plant) compared to all other treatments (Figure 1A), whilst no significant differences were observed regarding the whole root system dry mass among the treatments (Figure 1C). The shoot-to-root ratio, an important parameter of plant growth and indicator of resource prioritization and allocation within the plant body, closely resembled the shoot dry weight (Figure 1B).

Regarding the leaf chlorophyll content, “300” seedlings were the only to exhibit a high total chlorophyll content in all four leaves present, which ranged from 1.063 to 1.598 mg chlorophyll/gr leaf fresh weight when the sampling was carried out (day 7) (Figure 1D). A pattern typical of iron starvation emerged in the low-iron group (10 μM Fe): leaf 1 appeared fairly chlorotic, with a total chlorophyll content similar to that of leaves 3 and 4, since it had already entered senescence on day 7. No chlorosis was evident in the older non-senescent mature leaf (Leaf 2). More interestingly, the same –Fe pattern observed in plants subjected to a low Fe supply (group “10”) also appeared in plants grown under iron sufficiency (300 μM Fe) when treated with exogenous GA_3_ (Figure 1D). Although all leaves, except the youngest leaf (Leaf 4), of treatment “300G” had elevated total chlorophyll contents compared to the respective leaves of Fe-starved plants (treatment “10”), these differences were of no statistical significance. Oddly, a diverse, unique pattern developed when Fe-starved seedlings were treated with mepiquat chloride (“10M”). Surprisingly, in these plants, the first three leaves were even more severely chlorotic relative to their “10” counterparts, with a chlorophyll concentration of approximately 0.78 mg/gr fresh weight. In any case, however, “10M” did not exhibit a typical –Fe chlorosis pattern (Figure 1D).

#### 2.1.2. Root Length and Elongation Rate

**Figure 2:**
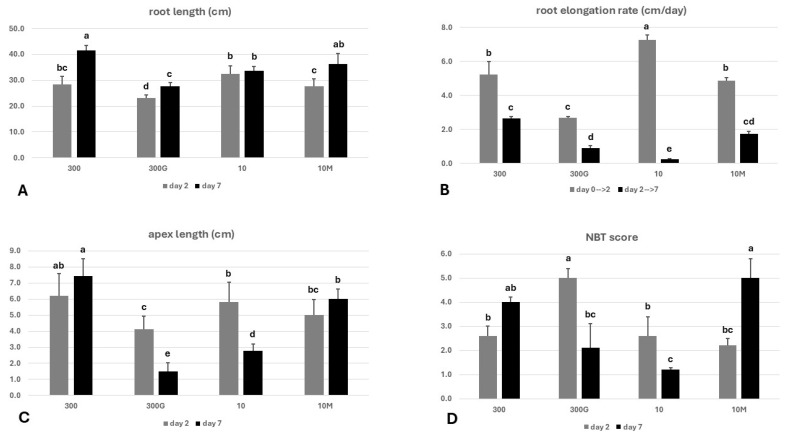
Root length (cm) (**A**), root elongation rate (cm/day) (**B**), root apex length (cm) (**C**), and Nitroblue Tetrazolium (NBT) staining for the in situ detection of O_2_**^.^**^−^ formation on the surface of root apices (**D**) of the first row of crown roots (CR1) of maize seedlings, among the four treatments (300, 10, 300G, 10M) on days 2 and 7. 300: maize seedlings grown in iron-sufficient conditions (300 μM Fe). 300G: maize seedlings grown under iron sufficiency (300 μM Fe) in the presence of exogenous gibberellic acid (G). 10: maize seedlings grown in iron-insufficient conditions (10 μM Fe). 10M: maize seedlings grown under iron insufficiency (10 μM Fe) but with the addition of the gibberellin biosynthesis inhibitor mepiquat chloride (M) in the growth medium. Mean ± SE. Different letters denote significant differences (Tukey’s multiple comparison test *p* < 0.005).

The length of first-row crown roots (CR1) was measured on days 2 and 7 (Figure 2A). Additionally, the elongation rate of these roots was determined from day 0 to day 2 and from day 2 to day 7 (Figure 2B). The CR1 roots of GA_3_-treated seedlings grown in ample Fe (300 μM Fe) were the shortest among all treatments on day 2 (having a mean length of 23.20 cm), which was in sharp contrast to Fe-starved plants (treatment “10”), in which CR1 roots were the lengthiest (with a mean length of 32.32 cm), although the differences from “300” roots were not significant (Figure 2A). This somewhat paradoxical acceleration of the root growth in “10” plants was also reflected in the significantly elevated elongation rate in those roots from day 0 to day 2 in comparison to the rest of the treatments (Figure 2B). On day 7, the trend regarding “300G” roots was maintained, and hence these were again the shortest among all treatments, differing significantly from the roots of plants grown either under Fe sufficiency (300 μM Fe) or Fe insufficiency (10 μM Fe) (Figure 2A). The highest elongation rates between days 2 and 7 were observed in the roots of “300” and “10M” seedlings (2.6 and 1.7 cm/day, respectively) (Figure 2B), while these two treatments also had lengthier CR1 roots on day 7, even though the differences from “10” were not of statistical significance (Figure 2A).

#### 2.1.3. Root Apex Length—BTR Phenotype

While no significant difference in length was observed between “300” and “10” apices on day 2, the addition of exogenous GA_3_ in the growth medium of Fe-sufficient plants (treatment “300G”) resulted in the development of the BTR phenotype from early on (day 2) (Figure 2C). On day 7, the apex length in the group “300” was 7.4 cm, significantly greater in relation to other treatments. Thus, a clear pattern had developed by day 7 regarding the emergence of the BTR phenotype. As expected, plants cultivated in a low-Fe-supply environment (treatment “10”) had developed this iron deficiency-related phenotype, with an apex length of 2.8 cm. Beyond “10” roots, the BTR phenotype could also be clearly observed in CR1 roots of GA_3_-treated plants grown in Fe abundancy (300 μM Fe), where the apex length was 1.5 cm. In an analogous manner, even though “10M” seedlings were grown under conditions of low Fe supply (10 μM Fe), their roots did not develop the BTR phenotype (apex length 6.0 cm), unlike their non-mepiquat-treated counterparts (treatment “10M”) (Figure 2C).

### 2.2. In Situ Superoxide Anion (O_2_**^.^**^−^) Detection on the Surface of Root Apices—Nitroblue Tetrazolium (NBT) Staining

The root apex was found to be the only site on the root surface with significant amounts of O_2_**^.^**^−^ formation, as indicated through NitroBlue Tetrazolium (NBT) staining (Figure 2D). On day 2, the NBT score value was most elevated on the surface of “300G” apices, being about twice as high as the other three treatments, while no statistically significant differences were present between the treatments “300”, “10”, and “10M”. This trend, however, was reversed on day 7. The formation of superoxide on the surface of “300G” and “10” apices was comparable but considerably lower as opposed to treatments “300” and “10M”, even though the difference between “300” and “300G” did not reach statistical significance (Figure 2D).

### 2.3. Iron Speciation on Root Surface: In Situ Detection of Ferric Iron (Fe^3+^) Deposition and Ferrous Iron (Fe^2+^) Formation on the Root Surface—Prussian Blue and Turnbull Blue Staining

**Figure 3:**
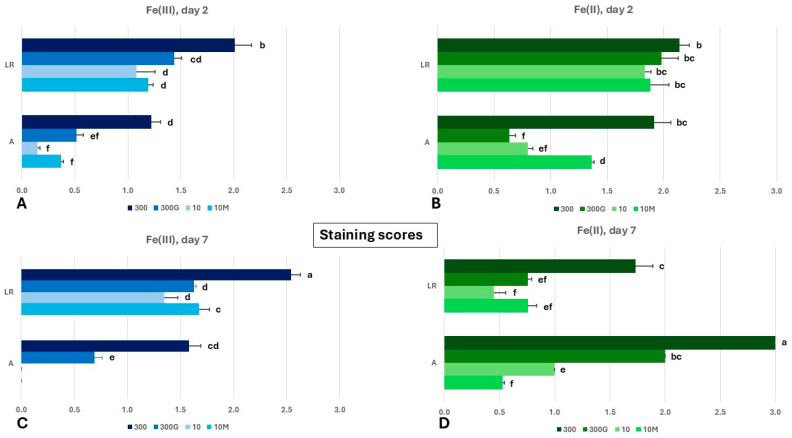
Prussian blue staining for ferric iron (Fe^3+^) deposition plaques (**A**,**C**) and Turnbull blue staining indicating ferrous iron (Fe^2+^) formation (**B**,**D**) in situ on the root surface of the apex (A) and the lateral root (LR) sectors on day 2 and day 7. For both Prussian blue and Turnbull blue, the stain coverage is expressed as a score accounting for both the extent of the root surface stained, as well as the intensity of the staining. 300: maize seedlings grown in iron-sufficient conditions (300 μM Fe). 300G: maize seedlings grown under iron sufficiency (300 μM Fe) in the presence of exogenous gibberellic acid (G). 10: maize seedlings grown in iron-insufficient conditions (10 μM Fe). 10M: maize seedlings grown under iron insufficiency (10 μM Fe) but with the addition of the gibberellin biosynthesis inhibitor mepiquat chloride (M) in the growth medium. Mean ± SE. Different letters denote significant differences (Tukey’s multiple comparison test *p* < 0.005).

On both sampling days (day 2 and day 7), ferric iron (Fe^3+^) deposition (Figures S1 and S2) was more evident in the lateral root sectors (LR) of all treatments (Figure 3A,C). Significantly higher score values of Fe^3+^ deposition were observed in both root sectors (LR, A) of “300” roots on day 2, reaching score values of 2, 0 and 1.2, respectively, while the scores did not differ between treatments “300G”, “10”, and “10M” in either root sector. On the other hand, on day 7, a significant increase in Fe^3+^ plaques was evident in the LR of “300” roots (reaching a score value of 2.5), indicating that the pattern observed on day 2 was maintained through day 7 (Figure 3C). A smaller yet significant increase in Fe^3+^ deposition was also apparent in the LR of Fe-starved plants grown in the presence of mepiquat chloride (treatment “10M”). No changes in ferric iron plaque levels were seen between day 2 and day 7 in the LR of treatments “10” and “300G” (with score values 1.3 and 1.6, respectively) (Figure 3C).

In contrast to Fe^3+^ deposition, which on both days predominantly occurred on the LRs, the formation of Fe^2+^ was more equally distributed on the root surface (Figures S1 and S2). On day 2, similar levels of Fe^2+^ formation could be seen in the LRs of all treatments (Figure 3B). However, considerable differences existed in the root apices (A), where a significantly higher score was found in “300” apices (score value 1.9), followed by treatment “10M” (score value 1.4). Ferrous iron (Fe^2+^) formation levels were similar in the apices of “10” and “300G” roots, reaching score values of 0.8 and 0.6, respectively (Figure 3B).

Notably, all root apices on day 7, apart from those in treatment “10M”, had significantly greater score values compared to their respective LR sectors (Figure 3D). Moreover, on day 7, Fe^2+^ formation occurred at similar levels in the LR of “300G”, “10”, and “10M” roots (with score values ranging from 0.4 to 0.8), being markedly lower in relation to the score value of 1.7 in treatment “300” (Figure 3B,D). While, in general, Fe^2+^ formation on day 7 preferentially occurred in the A, “10M” was the only treatment where Fe^2+^ formation levels were higher in the LR compared to the A (Figure 3D). It should be noted that, overall, both root sectors of “300” roots demonstrated elevated scores for both Fe^3+^ deposition and Fe^2+^ formation compared to their counterparts in other treatments.

### 2.4. Iron Concentration in Maize Shoots and Roots

**Figure 4:**
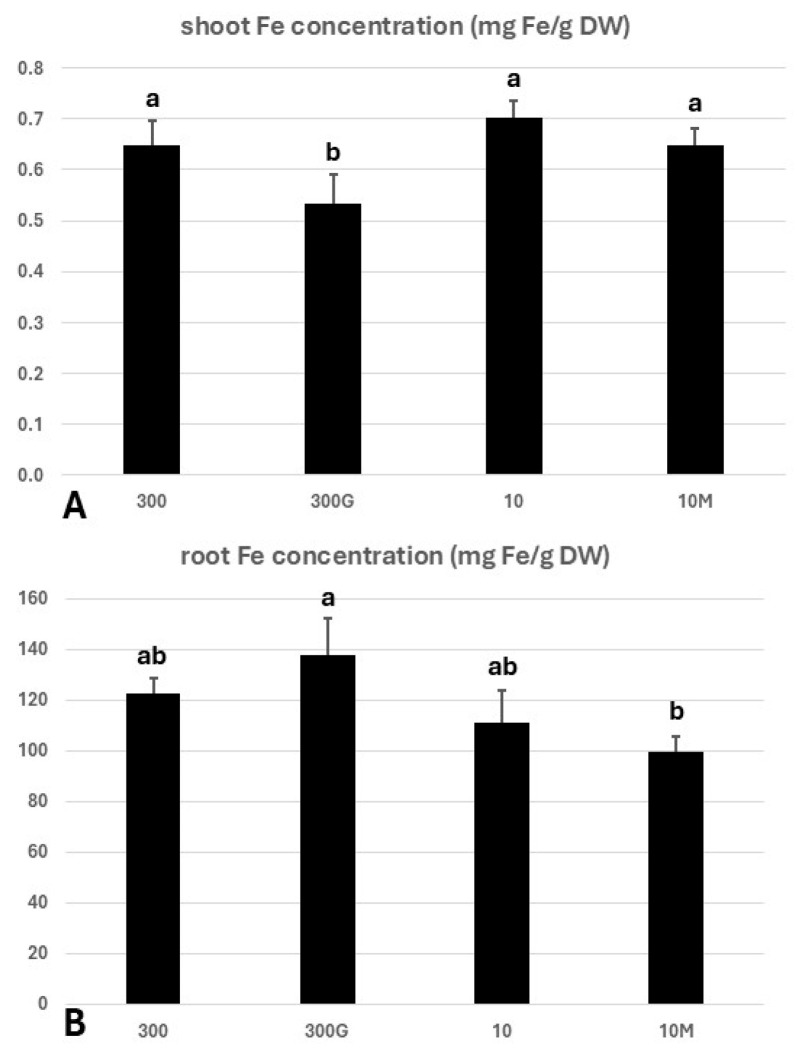
Shoot (**A**) and root (**B**) total iron concentration on day 7, given as milligrams of Fe per gram of shoot dry weight (mg Fe/g DW). 300: maize seedlings grown in iron-sufficient conditions (300 μM Fe). 300G: maize seedlings grown under iron sufficiency (300 μM Fe) in the presence of exogenous gibberellic acid (G). 10: maize seedlings grown in iron-insufficient conditions (10 μM Fe). 10M: maize seedlings grown under iron insufficiency (10 μM Fe) but with the addition of the gibberellin biosynthesis inhibitor mepiquat chloride (M) in the growth medium. Mean ± SE. Different letters denote significant differences (Tukey’s multiple comparison test *p* < 0.005).

Shoot and root iron concentrations (mg Fe/g of shoot dry weight) on day 7 are given in Figure 4. The shoots of GA_3_-treated plants grown in ample Fe (treatment “300G”) exhibited a significantly lower iron concentration (0.532 mg/g dry weight) compared to all other treatments. No statistically significant differences were observed between the shoots of “10”, “300”, and “10M” seedlings, although the Fe concentration in “10” shoots was slightly increased, despite the low Fe content of the growth medium.

Interestingly, the highest Fe concentration of 137 mg/g dry weight was found in the roots of “300G” seedlings. This was in sharp contrast to what was observed in the shoots of these plants. On the other hand, the root systems of “10M” plants showed the lowest Fe concentration among all treatments, although this difference was significant only compared to “300G”.

### 2.5. Gene Expression Profile of Strategy II and Strategy I Iron Uptake Genes in Maize

**Figure 5:**
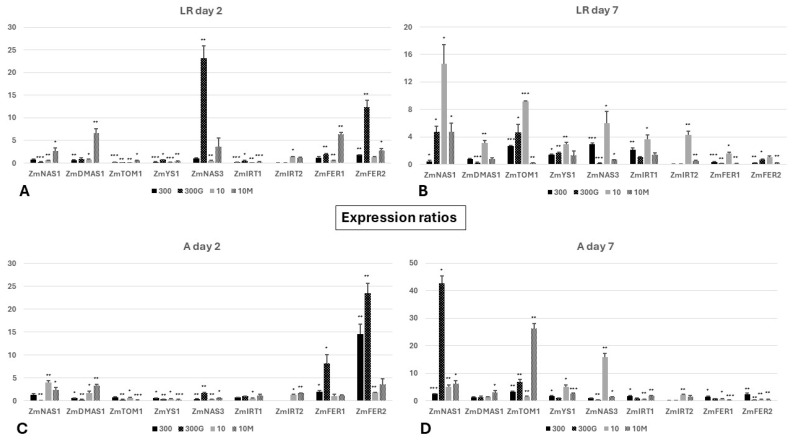
Gene expression ratios of strategy II (*ZmNAS1*, *ZmDMAS1*, *ZmTOM1*, *ZmYS1*) and strategy I (*ZmIRT1*, *ZmIRT2*) genes, as well as of the nicotianamine (NA) synthase gene *ZmNAS3* and ferritin genes *ZmFER1* and *ZmFER2* in the LR (**A**,**B**) and A (**C**,**D**) in the CR1 roots of maize seedlings on day 2 and on day 7. 300: maize seedlings grown in iron-sufficient conditions (300 μM Fe). 300G: maize seedlings grown under iron sufficiency (300 μM Fe) in the presence of exogenous gibberellic acid (G). 10: maize seedlings grown in iron-insufficient conditions (10 μM Fe). 10M: maize seedlings grown under iron insufficiency (10 μM Fe) but with the addition of the gibberellin biosynthesis inhibitor mepiquat chloride (M) in the growth medium. LR: lateral root sector, A: root apex, CR1: first row of crown roots. Mean ± SE. Asterisks indicate statistically significant overexpression or downregulation. *p* < 0.05 (*), *p* < 0.01 (**), *p* < 0.001 (***).

The two phytosiderophore (PS) biosynthesis genes studied were generally downregulated (*ZmDMAS1*) or remained unaltered (*ZmNAS1*) in both root sectors of “300” CR1 roots on day 2. However, when GA_3_ was added to the growth medium of seedlings grown in adequate Fe (“300G”), *ZmNAS1* was furthermore considerably downregulated in the whole root, and the same could be observed for *ZmDMAS1* in the A, compared to their non-GA_3_-treated counterparts. Iron limitation (treatment “10”) transcriptionally induced both *ZmNAS1* and *ZmDMAS1* exclusively in the apex (A), but the robust upregulation of these genes was evident in the LR in addition to the A when these plants were treated with the GA biosynthesis inhibitor mepiquat chloride (treatment “10M”). Interestingly, regarding the two strategy II uptake genes *ZmTOM1* and *ZmYS1*, both were downregulated in the whole root (LR, A) of all four treatments from day 0 to day 2, except *ZmTOM1* in the A of “300” plants, the expression levels of which had not fluctuated significantly (Figure 5A,C and Figure S3A,C).

For a more detailed depiction of the expressions at low ratio values, please see Figure S3.

On the other hand, on day 7, *ZmNAS1* and the strategy II Fe uptake genes were mostly upregulated across the entire CR1 roots of all four treatments (Figure 5B,D and Figure S3B,D). Yet this did not hold true for *ZmDMAS1*, as an overexpression was observed only in “10” roots regarding the LR sector and in the A of roots in the treatment “10M”. In line with the initial response, *ZmDMAS1* was further significantly suppressed in the LR of GA_3_-treated roots on day 7. Remarkably, *ZmNAS1* was considerably downregulated on day 7 in the LR of Fe-sufficient seedlings (treatment “300”), making this treatment the only case where neither of the two PS biosynthesis genes studied was induced in the LR (Figure 5B and Figure S3B). In sharp contrast to “300” roots, the concomitant upregulation of *ZmNAS1* and *ZmDMAS1* was documented in the LR of Fe-starved plants. Yet this response was not present in the A of these roots as, despite the upregulation of *ZmNAS1*, the expression of *ZmDMAS1* had not changed significantly from day 2 (Figure 5D and Figure S3D). The application of mepiquat chloride in Fe-deficient seedlings, however, further elevated the expression of both PS biosynthesis genes in the A, but not in the LR where *ZmDMAS1* expression did not change between days 2 and 7. Furthermore, in the LR of “300G” roots, a significant suppression of *ZmDMAS1* expression was evident on day 7 (Figure 5B and Figure S3B). Beyond the PS biosynthesis genes, the strategy II Fe uptake genes *ZmTOM1* and *ZmYS1* were robustly overexpressed in the apex of all treatments during the same period, with the only exception being *ZmYS1* in the A of “300G” roots (Figure 5D and Figure S3D). In the case of the LR sector, nevertheless, the application of mepiquat chloride attenuated the induction of *ZmTOM1* and *ZmYS1* observed in all other treatments, despite the low Fe nutrition of these seedlings (Figure 5B and Figure S3B).

The existence of *IRT1* homolog genes in maize has warranted a further investigation of their possible role in iron homeostasis under varying conditions of Fe availability. Most importantly, *ZmIRT1* expression was detected across the entire root in all treatments in both days studied. More specifically, *ZmIRT1* was preferentially expressed in the A on day 2, as it was downregulated in the LR of all seedlings (Figure 5A,C and Figure S3A,C). This pattern had shifted by day 7. A significant overexpression of *ZmIRT1* was detected in the LR of treatments “300” and “10” but not in the case of “300G” and “10M” plants (Figure 5B and Figure S3B). In the A, *ZmIRT1* was markedly upregulated in treatments “300” and “10M”, but not in the case of “300G”. Remarkably, *ZmIRT1* was even further downregulated on day 7 in “10” apices (Figure 5D and Figure S3D). Conversely, *ZmIRT2* was exclusively expressed under conditions of low external Fe levels, irrespective of mepiquat chloride addition (treatments “10” and “10M”), but not when plants were grown under Fe sufficiency (treatments “300” and “300G”). Even though Fe limitation led to the significant induction of *ZmIRT2* across the root on both days, the addition of mepiquat chloride seemed to attenuate this response. In this regard, a considerably lower expression of *ZmIRT2* was reported in the LR on day 2 and in the whole root on day 7 compared to non-treated Fe-deprived seedlings (treatment “10”). Only in the A on day 2 were *ZmIRT2* expression levels comparable between the two low-Fe treatments (‘10” and “10M” plants) (Figure 5C and Figure S3C).

Interestingly, *ZmNAS3* was significantly downregulated on day 2 in the A of “300”, “10”, and “10M” roots, while a robust induction was detected in “300G” (Figure 5C and Figure S3C). Likewise, a similar upregulation of *ZmNAS3* in the LR was seen again only in “300G” roots, as the induction observed in the case of “10M” did not reach statistical significance (Figure 5A and Figure S3A). On day 7, *ZmNAS3* was simultaneously upregulated in both root sectors solely in “10” roots. The induction detected initially in “300G” roots was not maintained from day 2 to day 7, and moreover *ZmNAS3* was strongly downregulated across the whole root (Figure 5B,D and Figure S3B,D).

On day 2, in Fe-sufficient plants (treatment “300”), *ZmFER1* was upregulated only in the A, whereas *ZmFER2* was induced in the entire root. When seedlings grown in similar conditions were treated with exogenous GA_3_ (treatment “300G”), the induction of *ZmFER1* in the A was even more intense, while it was also significantly upregulated in the LR. An enhanced upregulation across the whole root was also seen for *ZmFER2* in these plants (Figure 5A,C and Figure S3A,C). In “10M” seedlings, the two ferritin genes were induced in the LR, showing a different response from “10” roots. However, the addition of mepiquat chloride did not decisively impact ferritin expression in the A (Figure 5A,C and Figure S3A,C). On the other hand, on day 7, both *ZmFER1* and *ZmFER2* were significantly downregulated in the LR of all treatments except “10”, in which *ZmFER2* remained unaltered, and a marked upregulation could be seen for *ZmFER1* (Figure 5B and Figure S3B). Interestingly, both maize ferritin genes were found to be overexpressed in the A of “300” roots but significantly downregulated in the apices of all other treatments (Figure 5D and Figure S3D).

Finally, it should be noted that the expression of *ZmFRO2*, the closest maize homolog of Fe(III)-chelate Reductase 1 (FRO1) from *Arabidopsis thaliana*, was undetectable across the root on both sampling days.

### 2.6. Phylogenetic Analysis of Key Fe Homeostasis Transcriptional Regulators

#### 2.6.1. Phylogenetic Analysis of the Maize, Rice, and *Brachypodium distachyon* Iron Deficiency Response Element 1 Binding Factor (IDEF1) and Iron Deficiency Response Element 2 Binding Factor (IDEF2) Proteins and Their Homologs from *Arabidopsis thaliana*

**Figure 6:**
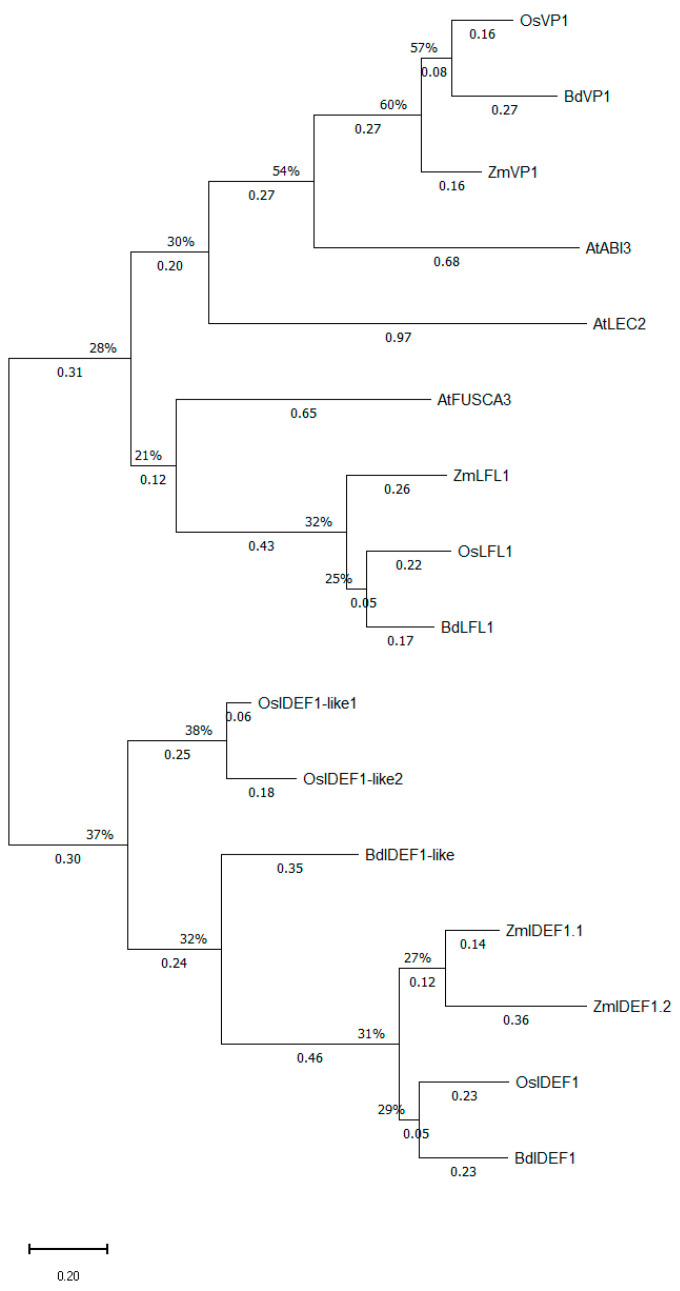
Evolutionary analysis using the maximum likelihood of the amino acid sequences of Iron Deficiency response Element 1 binding Factor (IDEF1) homologs and the putative IDEF1-like proteins from *Oryza sativa*, *Zea mays*, and *Brachypodium distachyon*, as well as the closely related ViviParous 1 (VP1) and LeaFy cotyLedon 1 (LFL1) amino acid sequences from maize, rice, and *Brachypodium distachyon*. The highly homologous ABscisic acid Insensitive 3 (ABI3), FUS3/FUSCA3, and LEafy Cotyledon 2 (LEC2) proteins from *Arabidopsis thaliana* are also included. Lists of the genes included in the phylogenetic tree are listed in Tables S1 and S2.

Even though their nomenclature might suggest that IDEF1 and IDEF2 are evolutionarily related, this impression is not accurate. It has already been shown that OsIDEF1 belongs to the ABscisic acid Insensitive 3/ViviParous 1 (ABI3/VP1) family of transcription factors in rice [2], whereas OsIDEF2 is a member of the NAC family [No apical meristem (NAM)/*Arabidopsis* transcription activation factor (ATAF1/2)/Cup-shaped cotyledon 2 (CUC2)] [23]. Given their unrelated nature, the construction of two separate phylogenetic trees was deemed necessary. The amino acid sequences of OsIDEF1 (NP_001409295.1) and OsIDEF2 (XP_015639254.1) were leveraged to reveal all IDEF1 and IDEF2 homolog proteins in maize, respectively. As shown in Figure 6, ZmIDEF1.1 (Zm00001eb198710) and ZmIDEF1.2 (Zm00001eb259870) are the two IDEF1 paralogous proteins in maize, which form a distinct subgroup along with their orthologs from rice (OsIDEF1) and *Brachypodium distachyon* (BdIDEF1) within the IDEF1/IDEF1-like clade. In conformity with what is found for OsIDEF1, the Maize Genome Database annotates Zm00001eb198710 [P002] (369 a.a.) as ABI3-VP1 transcription factor 47 (ZmABI47) and Zm00001eb259870 [P002] (322 a.a.) as ABI3-VP1 transcription factor 49 (ZmABI49). Moreover, the BLASTing of the IDEF1 amino acid sequences form rice, maize, and *Brachypodium distachyon* in their respective genomes revealed the presence of two highly homologous proteins to OsIDEF1 in rice and one such protein strongly resembling BdIDEF1 in *Brachypodium*. Despite their similarity to IDEF1 members, these newly identified proteins are conspicuously distinguishable from the IDEF1 subgroup, suggesting that they are probably not IDEF1 paralogs. Since the exact functions of these transcription factors remained unknown by the time this analysis was conducted, we decided to classify these proteins under the term IDEF1-like. Hence, XP_025880492.1 and NP_001406901 from rice were designated as OsIDEF1-like1 and OsIDEF1-like2, respectively, whereas BdIDEF1-like was the name proposed for XP_003579476.4 from *Brachypodium distachyon* (Figure 6). Beyond AtABI3, the BLAST performed in the Arabidopsis genome using the grass IDEF1 amino acid sequences also yielded the B3 domain, containing proteins AtLEC2 (LEafy Cotyledon 2) and AtFUSCA3 (AtFUS3), which both share a high degree of similarity with AtABI3. According to Figure 6, OsLFL1 (NP_001384109.1), ZmLFL1 (Zm00001eb361390 [P_001]), and BdLFL1 (XP_0014754315.1) are the homologs of AtFUSCA3 in rice, maize, and *Brachypodium*, while OsVP1 (XP_025878207.1), ZmVP1 (Zm00001eb143690 [P_001]), and BdVP1 (XP_010232722.1) are proteins homologous to AtABI3, but are also closely related to AtLEC2. Several versions of the phylogenetic tree of Figure 6 were tested prior to the final version provided here, and the incorporation of the ViviParous 1 (VP1) and LeaFy cotyLedon 1 (LFL1) orthologs for rice, maize, and *Brachypodium* was regarded as necessary for the clearest and most accurate illustration of the evolutionary relationships between the proteins of the IDEF1/IDEF1-like clade and their closest, non-grass B3 domain homologous TFs from *Arabidopsis thaliana*.

**Figure 7:**
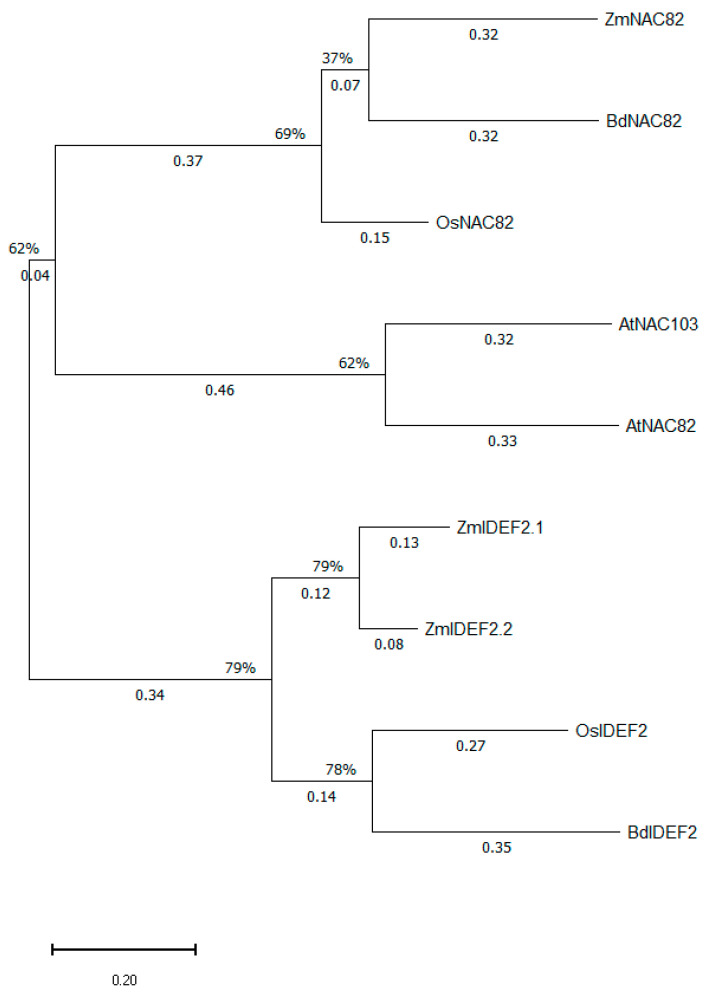
Evolutionary analysis using the maximum likelihood method referring to the Iron Deficiency response Element 2 binding Factor (IDEF2) from *Oryza sativa*, *Zea mays*, and *Brachypodium distachyon*. The *Arabidopsis thaliana* No apical meristem (NAM)/*Arabidopsis* transcription activation factor (ATAF1/2)/Cup-shaped cotyledon 2 (CUC2) 82 and 103 (AtNAC82 and AtNAC103) sequences and the highly homologous OsNAC82, ZmNAC82, and BdNAC82 were also included for comparison. Lists of the genes included in the phylogenetic tree are listed in Tables S1 and S2.

Two IDEF2 paralogs are present in maize (Figure 7). These are the NAC transcription factor members Zm00001eb288420 [P001] (ZmNAC112) and Zm00001eb349010 [P001] (ZmNAC38), dubbed here as ZmIDEF2.1 (452 a.a.) and ZmIDEF2.2 (445 a.a.), respectively, based on their sequence homology to OsIDEF2 (XP_015639254.1). BdIDEF2 (XP_003568407.1) is the sole OsIDEF2 orthologous protein in the model grass *Brachypodium distachyon*. The BLAST of the OsIDEF2 amino acid sequence in the genome of *Arabidopsis thaliana* identified AtNAC82 and AtNAC103 as the proteins exhibiting the highest homology to IDEF2. In spite of this, the BLASTing of maize, rice, and *Brachypodium* IDEF2 homologs against their respective genomes yielded the highly homologous XP_066165793.1 (rice), Zm00001eb076630 [P001] (maize), and XP_003581627.1 (*Brachypodium distachyon*). Remarkably, the *Arabidopsis thaliana* AtNAC82 and AtNAC103 share a higher level of homology with these three grass proteins compared to IDEF2 paralogs. Furthermore, the protein alignment results indicated that XP_066165793.1, Zm00001eb076630 [P001], and XP_003581627.1 were all slightly more similar to AtNAC82 as opposed to AtNAC103, and as a result it was postulated that all these three proteins are likely the NAC82 homologs in grasses (Figure 7).

#### 2.6.2. Phylogenetic Analysis of the Maize, Rice, and *Brachypodium distachyon* Iron-Related Transcription Factor 2 (IRO2) Proteins and Their Ib Clade bHLH Homologs from *Arabidopsis thaliana*

**Figure 8:**
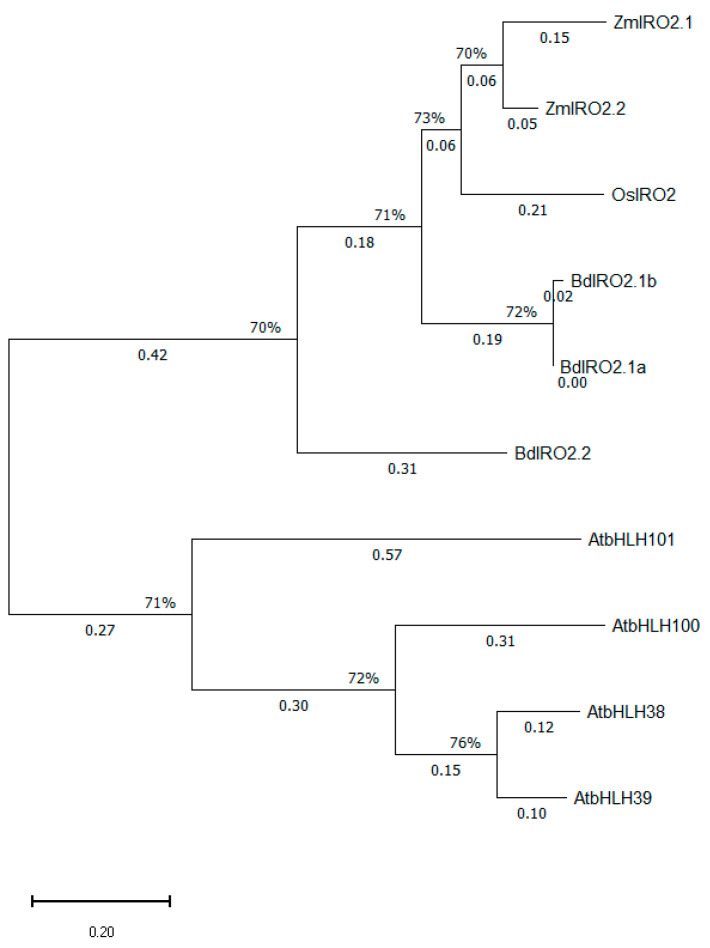
Evolutionary analysis using the maximum likelihood method including the protein sequences of the Ib clade of basic Helix–Loop–Helix (bHLH) transcription factors from *Arabidopsis thaliana* (AtbHLH38/39/100/101) and the Iron-related transcription factor 2 (IRO2) members from *Oryza sativa*, *Zea mays*, and *Brachypodium distachyon*, the Ib bHLH homologous proteins in grasses. Lists of the genes included in the phylogenetic tree are listed in Tables S1 and S2.

The protein sequence of OsIRO2 (XP_015612709.1) served as a template to reveal IRO2 homologs in maize and *Brachypodium*. The BLAST analysis performed yielded two IRO2 paralogs in maize, Zm00001eb140680 [P001] (237 a.a.), dubbed as ZmIRO2.1, and Zm00001eb362800 [P001] (246 a.a.), termed ZmIRO2.2, as well as three IRO2 paralogous proteins in the model grass *Brachypodium distachyon*: XP_003565088.1 (BdIRO2.1a), XP_003565095.1 (BdIRO2.1b), and XP_024317684.1 (BdIRO2.2) (Figure 8). Notably, BdIRO2.1a and BdIRO2.1b are closely related to each other, and both the respective genes were found to be located on chromosome 2, in relatively close proximity. As shown in Figure 8, the bHLH transcription factors ZmIRO2.1 (ZmbHLH126) and ZmIRO2.2 (ZmbHLH54), along with OsIRO2 as well as BdIRO2.1a, BdIRO2.1b, and BdIRO2.2, are collectively homologous to the *Arabidopsis thaliana* Ib Subgroup bHLH transcription factors (AtbHLH38/39/100/101). Interestingly, Zm00001eb140680 (ZmIRO2.1) was the only hit obtained when BLAST was performed in maize for both AtBHLH38 and AtBHLH101, while Zm00001eb362800 (ZmIRO2.2) was the only result in the case of AtBHLH39 and AtBHLH100.

#### 2.6.3. Phylogenetic Analysis of the Maize, Rice, and *Brachypodium distachyon* Iron-Related Transcription Factor 3 (IRO3) Proteins and Their IVb Clade bHLH Homologs from *Arabidopsis thaliana*

**Figure 9:**
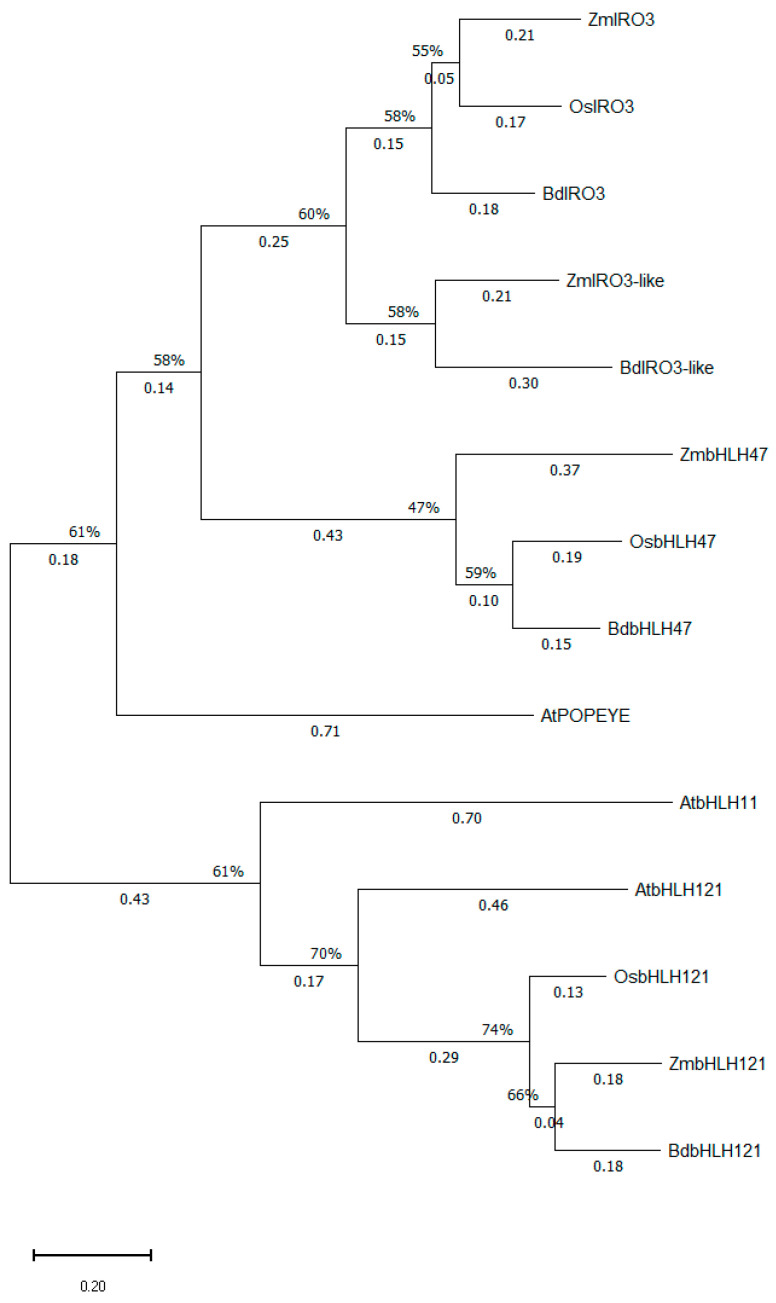
Evolutionary analysis using the maximum likelihood method of the IVb clade basic Helix–Loop–Helix (bHLH) members AtPYE (AtPOPEYE/AtbHLH47), AtbHLH11, and AtbHLH121 from *Arabidopsis thaliana* and their homologous proteins in *Oryza sativa*, *Zea mays*, and *Brachypodium distachyon*, including the Iron-related transcription factor 3 (IRO3) members. Lists of the genes included in the phylogenetic tree are listed in Tables S1 and S2.

The phylogenetic tree of Figure 9 illustrates the evolutionary relationship between the IVb clade basic Helix–Loop–Helix (bHLH) members from *Arabidopsis thaliana* [AtPYE (AtPOPEYE/AtbHLH47), AtbHLH11, and AtbHLH121] and their homologous proteins from *Oryza sativa*, *Zea mays*, and *Brachypodium distachyon*. The bHLH transcription factor Zm00001eb018460 [P004] (268 a.a.), annotated as ZmbHLH185 in the MGDB, is the orthologous protein of the rice OsIRO3, in agreement with previous findings [24]. Similarly to rice and maize, BdIRO3 (XP_003557817.1) is the sole IRO3 homologous protein present in the grass model *Brachypodium distachyon*. Furthermore, the bHLH transcription factors Zm00001eb328250 [P003] (maize) and XP_003562634.1 (*Brachypodium distachyon*) were found to be closely related to the ZmIRO3 and BdIRO3 paralogs and were termed as ZmIRO3-like and BdIRO3-like, respectively. As shown in Figure 9, ZmIRO3-like and BdIRO3-like form a distinct subgroup within the IRO3/IRO3-like clade. Remarkably, in addition to the IRO3/IRO3-like clade members, the bHLH proteins Zm00001eb167310 [P003] (ZmbHLH187) from maize, XP_015617819.1 (OsbHLH062-like) from rice, and XP_010237571.1 (BdbHLH062) from *Brachypodium* can also be considered as orthologs of AtPYE/AtbHLH47. In order to highlight their homology to AtbHLH47 (AtPYE), these elusive transcription factors were labeled here as OsbHLH47 (XP_015617819.1), ZmbHLH47 (Zm00001eb167310 [P003]), and BdbHLH47 (XP_010237571.1). On the other hand, the maize bHLH transcription factor Zm00001eb159750 [P001] (ZmbHLH166), along with XP_015626280.1 (rice) and XP_024317977.1 (*Brachypodium*), is the grass homolog of the *Arabidopsis thaliana* IVb clade bHLH member AtbHLH121 (AtURI).

#### 2.6.4. Phylogenetic Analysis of the Strategy I Fe Homeostasis Regulator FER-like Iron Deficiency-Induced Transcription Factor (FIT) from *Arabidopsis thaliana* and Its Homologous Proteins from Maize, Rice, and *Brachypodium distachyon*

**Figure 10:**
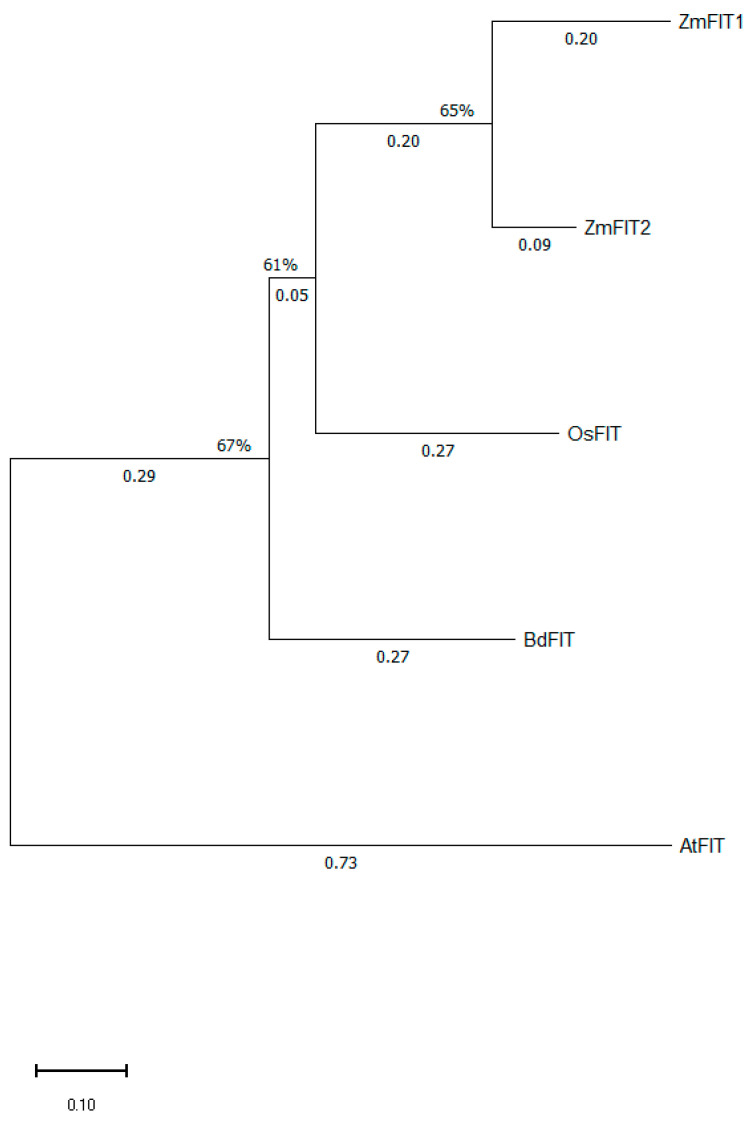
Evolutionary analysis using the maximum likelihood method among the FER-like Iron Deficiency-Induced Transcription Factor (FIT) protein sequences of *Arabidopsis thaliana*, *Oryza sativa*, *Brachypodium distahyon*, and *Zea mays*. Lists of the genes included in the phylogenetic tree are listed in Tables S1 and S2.

Although AtFIT and its orthologs in other plant species are associated with the regulation of strategy I, FIT homologous genes are also present in the genomes of strategy II plants as well as in rice, a mixed strategy plant. Here, we report that, similarly to rice, the model grass *Brachypodium distachyon* possesses one AtFIT homologous protein (BdFIT), while maize possesses two FIT homologous proteins, namely Zm00001eb085690 [P001] (ZmbHLH101), measuring 399 a.a. long, and Zm00001eb420910 [P001] (ZmbHLH100), with a length of 381 a.a. Even though Zm00001eb420910 [P001] is the best BLAST hit for both AtFIT (NP_850114.1) and OsFIT (XP_015634338.1) in maize, Wairich et al. [25] have referred to Zm00001eb085690 as ZmFIT1, and thus we chose to denominate Zm00001eb420910 [P001] as ZmFIT2 (Figure 10).

### 2.7. Expression Profile of Core Iron Homeostasis Transcription Factor Genes

**Figure 11:**
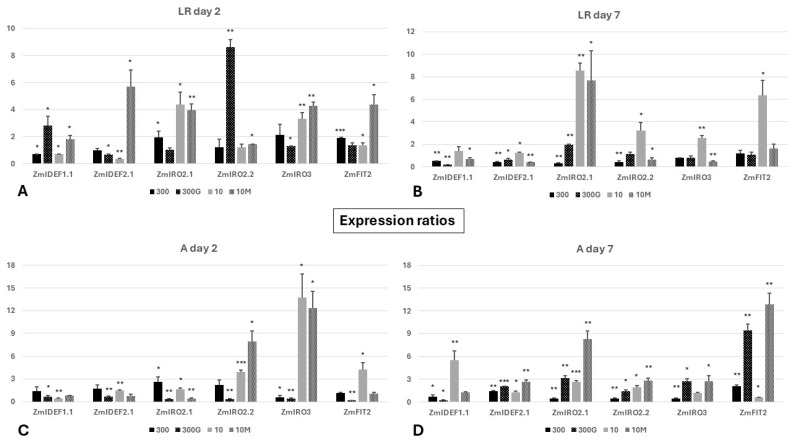
Gene expression ratios of Fe homeostasis-regulating transcription factors in the LR (**A**,**B**) and A (**C**,**D**) in the CR1 roots of maize seedlings on day 2 and day 7. Genes included the ABI member TF *ZmIDEF1.1*, the NAC member TF *ZmIDEF2.1*, and the bHLH TFs *ZmIRO2.1*, *ZmIRO2.2*, *ZmIRO3*, and *ZmFIT2*. 300: maize seedlings grown in iron-sufficient conditions (300 μM Fe). 300G: maize seedlings grown under iron sufficiency (300 μM Fe) in the presence of exogenous gibberellic acid (G). 10: maize seedlings grown in iron-insufficient conditions (10 μM Fe). 10M: maize seedlings grown under iron insufficiency (10 μM Fe) but with the addition of the gibberellin biosynthesis inhibitor mepiquat chloride (M) in the growth medium. LR: lateral root sector, A: root apex, CR1: first row of crown roots. Mean ± SE. Asterisks indicate statistically significant overexpression or downregulation. *p* < 0.05 (*), *p* < 0.01 (**), *p* < 0.001 (***).

In plants grown under Fe sufficiency (treatment “300”), *ZmIDEF1.1* was slightly, though nevertheless significantly, downregulated in the LR on day 2, while expression in the A remained constant. During this same period, the expression of *ZmIDEF2.1* did not fluctuate considerably in these roots (Figure 11A,C and Figure S4A,C). In contrast, in Fe-starved maize seedlings (treatment “10”), *ZmIDEF1.1* was markedly downregulated in both root sectors, while *ZmIDEF2.1* was overexpressed in the root apex and downregulated in the LR. Moreover, in the treatment “300G”, besides the upregulation seen in the case of *ZmIDEF1.1* in the LR, both *ZmIDEF1.1* and *ZmIDEF2.1* were downregulated across the whole root on day 2. Lastly, *ZmIDEF1.1* and *ZmIDEF2.1* were induced in the LR but not in the A of “10M” roots, where no significant alterations in gene expression could be detected (Figure 11A,C and Figure S4A,C).

For a more detailed depiction of the expressions at low ratio values, please see Figure S4.

On day 7, *ZmIDEF1.1* and *ZmIDEF2.1* were generally downregulated in the LR of all treatments, with the exception of “10” plants, where both genes were mildly upregulated, albeit significantly in the case of *ZmIDEF2.1* (Figure 11B and Figure S4B). On the other hand, regarding the A, an overexpression of *ZmIDEF1.1* was observed exclusively in plants cultivated under a low Fe supply (treatment “10”). In the root apices of seedlings placed in Fe abundance (treatments “300”, “300G”), *ZmIDEF1.1* was downregulated, and this response was stronger in GA_3_-treated plants. In contrast to the LR, *ZmIDEF2.1* was considerably upregulated in the apices of all treatments from day 2 to day 7 (Figure 11D and Figure S4D).

With regard to the *IRO2* and *IRO3* maize homolog genes studied here, *ZmIRO2.1* and *ZmIRO3* appeared to be upregulated on day 2 in the LR of plants experiencing Fe sufficiency (treatment “300”), but only in the case of *ZmIRO2.1* was this overexpression significant (Figure 11A and Figure S4A). The marked induction of *ZmIRO2.1* and *ZmIRO3* but not *ZmIRO2.2* was observed when plants were grown in a low-Fe environment (“10” roots), while the addition of mepiquat chloride (treatment “10M”) led to the upregulation of all three *ZmIRO* paralogs in the LR. Moreover, in the LR of “300G” roots, *ZmIRO2.2* and *ZmIRO3*, but not *ZmIRO2.1*, exhibited significant upregulation by day 2 (Figure 11A and Figure S4A). At the same time, in the A of “300” roots, *ZmIRO2.1* and *ZmIRO3* were markedly induced and downregulated, respectively. The expression levels of *ZmIRO2.2* were elevated but not to the point of statistical significance (Figure 11C and Figure S4C). Furthermore, in the A of roots in treatment “10”, expression was significantly enhanced in *ZmIRO2.1*, *ZmIRO2.2*, and *ZmIRO3* from day 0 to day 2. The treatment of seedlings cultivated in sufficient Fe with exogenous GA_3_ was associated with a considerable downregulation of all three genes. Meanwhile, *ZmIRO2.1* and *ZmIRO3* were overexpressed, whereas *ZmIRO2.1* levels were diminished in “10M” apices (Figure 11C and Figure S4C). In the LR of “300” roots, *ZmIRO2.1* and *ZmIRO2.2* were downregulated on day 7, while the expression of *ZmIRO3* did not change significantly (Figure 11B and Figure S4B). *ZmIRO2.1*, however, was induced in the LR of all other treatments. In addition to treatment “300”, *ZmIRO2.2* was also transcriptionally suppressed in the LR of “10M” roots. The only case where all three *ZmIRO* genes were concomitantly upregulated in the LR on day 7 was observed in seedlings subjected to a low Fe supply (“10”). Interestingly, treatment with the GA biosynthesis inhibitor mepiquat chloride (“10M” plants) was associated with a considerable reduction in *ZmIRO2.2* and *ZmIRO3* expression levels. In the lateral root sectors of “300G” roots, no significant changes in the expression levels of *ZmIRO2.2* and *ZmIRO3* were detected (Figure 11B and Figure S4B). Regarding the root apex (A) during the same period (day 2 to day 7), *ZmIRO2.1* and *ZmIRO2.2* were significantly upregulated in treatments “10”, “300G”, and “10M”. In seedlings grown under Fe sufficiency (treatment “300”), both *ZmIRO2* paralogs were downregulated (Figure 11D and Figure S4D), consistent with the response observed in the LR. Similarly, the transcription levels of *ZmIRO3* were considerably reduced in “300” LR sectors. By contrast, *ZmIRO3* expression was strongly upregulated in the apices of “300G” and “10M” plants, while no variation was reported in the A of plants grown under a low-Fe status (treatment “10”) (Figure 11D and Figure S4D).

Finally, of the two *FIT* homolog genes present in the maize genome, only *ZmFIT2* was expressed, since no expression could be detected for *ZmFIT1* in the roots of any treatment. In contrast, *ZmFIT2* was overexpressed in the LR of “300”, “10”, and “10M” plants and in the A of “10” roots on day 2, whereas at the same time *ZmFIT2* was downregulated in the A of “300G” roots (Figure 11A,C and Figure S4A,C). A significant variation in *ZmFIT2* transcriptional levels from day 2 to day 7 was absent from the LR of all treatments but “10” roots, where *ZmFIT2* was significantly upregulated (Figure 11B and Figure S4B). In sharp contrast, the opposite pattern was seen in the A. The expression of *ZmFIT2* was significantly enhanced in the A of all treatments except “10” roots, in which a significant downregulation was reported (Figure 11D and Figure S4D).

## 3. Discussion

### 3.1. Gibberellin Modulation Shapes Iron Deficiency-Associated Phenotypic Responses in Maize Seedlings

Reliably determining whether a plant experiences iron deficiency at the organismal level and/or in specific tissues requires taking into account the physiological and molecular signatures of Fe starvation, in addition to phenotypic manifestations (chlorosis predominantly in younger leaves, stunted growth, decreased biomass accumulation, and reduced shoot-to-root dry weight ratio [20,26,27,28]). As expected, seedlings grown under a low-Fe status (10 μM Fe), but not under Fe sufficiency (300 μM), had developed a typical Fe deficiency phenotype by day 7 (Figure 1A,B,D). Remarkably, however, the application of exogenous GA_3_ under Fe-sufficient conditions (“300G”) reproduced key –Fe symptoms, including marked reductions in the shoot dry weight and the shoot/root dry weight ratio, as well as the emergence of a chlorosis pattern, reminiscent of “10” leaves (Figure 1D). Analogous effects have been observed in GA_3_-treated rice [15,16]. On the other hand, mepiquat chloride-treated seedlings grown under iron insufficiency (“10M”) did not display a clear –Fe chlorosis pattern, as all leaves were equally chlorotic. This suggests that GA biosynthesis inhibition alters the manifestation of Fe deficiency symptoms in the shoot by dampening the contrast in chlorophyll content between younger and older leaves. It should be nevertheless notedthat, although the application of mepiquat chloride has been associated with increases in chlorophyll content, this has been observed only under non-Fe-limiting conditions [29,30,31]. Low iron availability also strongly influences root growth (Figure S5) by adversely affecting root length and the elongation rate (Figure 2A,B), consistent with previous findings [19,32,33]. This is likely due to a decline in cell division rates in the root apical meristem, as seen in other species [34,35,36]. Reductions in root length by –Fe were demonstrated to be greater in iron-inefficient maze genotypes [37]. In their work, Benke et al. [20] have identified a single maize QTL (“*jpsb79-umc60*”) associated with decreased root length under Fe deficiency (RL). An in silico analysis of this locus (Dataset S1) provides genetic evidence for the involvement of GAs in root length determination in relation to the Fe nutritional status, as the gene encoding GRAS23 is among the four genes contained in the “*psb79-umc60*” locus. GRAS23 is a member of the GRAS family transcription factors, which are involved in GA signaling [38]. The effect of GA modulation on root growth can be seen in 300G plants, in which treatment with GA_3_ significantly compromised root growth (Figure 2B) compared not only to their non-treated counterparts (“300”) but also to Fe-starved seedlings (“10”), despite a sufficient Fe supply. On the other hand, the inhibition of GA biosynthesis in maize seedlings grown under Fe limitation restored the root growth metrics studied (Figure 2A,B). These results contradict previous findings in rice, in which GA_3_ treatment promoted root growth and elongation while the inhibition of GA synthesis negatively affected these parameters [21]. It is possible that the contrasting adaptation response to GA modulation between the two grass species stems from differences in Fe solubility under distinct edaphic conditions.

More recently, –Fe-induced alterations of the maize root architecture have been described. Iron starvation leads not only to enhanced lateral root formation, but also to shorter root apices due to the ectopic emergence of lateral roots in greater proximity to the root tip, and this phenotype has been termed as BTR (Branching at the Terminal 5 cm of Root) [19,20]. In accordance with these observations, the apex length of CR1 roots was significantly decreased after 7 days of Fe starvation, and the BTR phenotype developed in the roots of plants grown under Fe insufficiency (10 μM Fe) but not in Fe-sufficient conditions (300 μM Fe) (Figure 2C). An in silico analysis of the BTR phenotype-associated locus “*chrom7-glb1*” identified two transcription factor genes involved in GA signaling, providing a genetic link of this –Fe phenotype to gibberellins [19]. Interestingly, the exogenous application of GA_3_ to plants cultivated in 300 μM Fe resulted in markedly shorter apices as early as two days after treatment initiation. By day 7, “300G” CR1 roots had developed the BTR phenotype, which was found to be even more severe compared to Fe-starved seedlings (treatment “10”). Inversely, the addition of mepiquat chloride in the nutrient solution of seedlings cultivated in a low-Fe status (“10M”) prevented the BTR phenotype and restored the apex length (Figure 2C, day 7).

Beyond phenotypic manifestations, whether a plant experiences iron deficiency can be traced to the expression patterns of *NAS1* and *YS1* in the roots, the concomitant upregulation of which serves as an Fe starvation marker [39,40,41,42]. Nevertheless, it is important to highlight that the studies establishing *ZmNAS1* and *ZmYS1* expression patterns as a marker of the internal Fe status did not distinguish between the lateral root zone and the root apex, but rather intact roots were obtained in which the apex only constitutes a relatively small fraction of the total root tissue. As anticipated, *ZmNAS1* and *ZmYS1* were overexpressed in the whole root of seedlings cultivated under limited iron supply (“10”) on day 7 (Figure 5B,D and Figure S3B,D), confirming systemic Fe starvation after a week under these conditions. Furthermore, in seedlings grown either under Fe sufficiency (treatment “300”) or in a low-Fe status in the presence of mepiquat chloride (“10M” plants), *ZmNAS1* and *ZmYS1* were simultaneously overexpressed only in the A, from day 2 to day 7 (Figure 5D). In the case of “300” seedlings, this is somewhat perplexing, as these plants were placed in Fe abundance, their phenotype indicated iron sufficiency, and *ZmNAS1* was significantly downregulated in the LR on day 7, which suggests that the largest part of the root was not subjected to iron deprivation (Figure 5B and Figure S3B). The apices in both these treatments, however, exhibited increased elongation rates, which could cause a local Fe shortage (Figure 2B), as rapidly growing root tips are strong Fe sinks. Iron is indeed crucial for meristematic cell function and cell division [43,44], and the nucleoli of actively dividing plant cells are particularly abundant in Fe [45]. Moreover, even though chlorosis was evident in all leaves of “10M” seedlings (Figure 1D), and despite the reduced root Fe concentration (Figure 4), these plants did not experience intense iron starvation (*ZmNAS1* and *ZmYS1* expression patterns in the LR: Figure 5B and Figure S3; no BTR phenotype: Figure 2C) and were able to maintain a robust root growth (Figure 2A,B). Therefore, the application of mepiquat chloride might have shifted the iron homeostasis of maize seedlings grown under Fe limitation to a new, lower Fe status, “equilibrium”, by readjusting the Fe use efficiency (FeUE). Whether the mitigation of the –Fe response in the LR of “10M” plants is the result of a negative shoot-borne signal, possibly LODIS (Long-Distance inhibitory Iron Signal) [46,47,48], or due to the suppression of a long-distance –Fe signal remains elusive.

**Figure 12:**
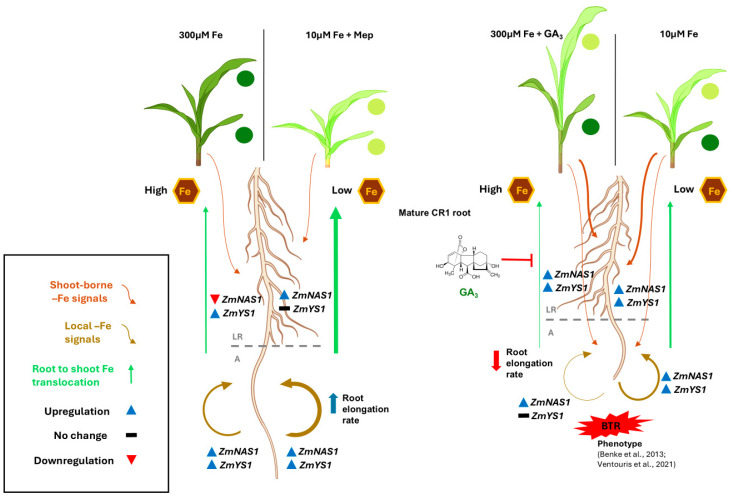
Illustration depicting the impact of iron deprivation and GAs on maize shoot and root phenotypes. Iron limitation (10 μM Fe) and GA_3_ treatment of plants cultivated in ample iron (300 μM Fe) both result in the development of a typical –Fe pattern, with chlorosis evident in the younger leaves. In addition, CR1 roots were shorter, with lateral roots emerging ectopically in close proximity to the root tip (BTR phenotype) [19,20]. The addition of mepiquat chloride to Fe-starved plants blunted this response, resulting in decreased but comparable chlorophyll contents between younger and older leaves, while mepiquat treatment also restored the root growth and prevented the advancement of the BTR phenotype despite the low external Fe levels (10 μM Fe). The Fe status of the shoot strongly impacts the responses of the LR sector, likely via long-distance shoot-borne –Fe signal(s). The influence of the shoot to the lateral root zone (LR) sector, irrespective of external Fe levels, is reflected by the expression profile of *ZmNAS1* and *ZmYS1*, the concomitant expression of which indicates iron starvation. On the other hand, the effects of local –Fe signals are more evident in the root apex (A), where Fe demand fluctuates depending on root growth. Thus, responses in the A are the outcome of the dynamic interaction between long-distance, shoot-derived, and local –Fe signals. Gibberellins negatively regulate the translocation of iron from the roots towards the shoot, leading to the high accumulation of Fe in roots. This illustration refers to mature CR1 roots (day 7). Line width is analogous to effect strength and/or intensity. Light green circles are used to indicate leaf chlorosis, while dark green is used to represent non-chlorotic leaves. Shoot and root illustrations adapted from BioRender.com.

Interestingly, *ZmTOM1* and *ZmYS1* were also concurrently upregulated in the lateral root zone (LR) of “300G” roots (Figure 5B), despite the increased root iron concentration (Figure 4) and the fact that these seedlings were grown in Fe abundance, suggesting that long-distance –Fe signals from the iron-starved shoot likely override weaker local signals of iron excess to modulate this response. In agreement, high root iron levels do not suppress iron acquisition genes in *Arabidopsis*, but rather this is mediated by a shoot-derived Fe signal reaching the roots via the phloem [46], highlighting the crucial role of the shoot in the orchestration of Fe uptake in the roots [49]. Such signals have also been associated with the induction of *AtIRT1* in GA_4_-treated plants under Fe sufficiency or deprivation [18]. The leaf vasculature has emerged as a major Fe sensing organ, responding to varying iron levels more rapidly than the root tissues [50]. Several studies have identified the ubiquitous IMA/FEP peptides as candidate shoot-borne –Fe signals, which are positive regulators of the –Fe response in roots [51,52,53,54]. The lack of an analogous expression pattern in the apex (A) is consistent with the high external Fe levels, together with the decreased Fe demand due to the low root elongation rate (Figure 2B, Figure 5C,D and Figure S3C,D). Together, these observations suggest that responses in the LR sector predominantly reflect the Fe status of the shoot, while the apex integrates systemic Fe signals with local signals shaped by the Fe demand in the apex (Figure 12).

### 3.2. Ferric Iron Uptake Pathway: Phytosiderophore Biosynthesis and Strategy II Fe Uptake Genes Are Differentially Regulated by GA, Depending on Root Developmental Stage and Sector

The root apex has been identified as the primary site of both PS release and the uptake of the PS-Fe^3+^ complexes under –Fe conditions in barley [55,56]. In line with this, *ZmNAS1* and *ZmDMAS1* were rapidly induced in the apex under low Fe (10 μM), but not in seedlings transferred into Fe abundance (300 μM Fe) (Figure 5C and Figure S3C). As the iron deprivation stress progressed (day 7), this trend was further reinforced in the A of “10” roots, while *ZmNAS1* was additionally significantly upregulated in the lateral root zone (Figure 5B,D and Figure S3B,D). Unlike PS biosynthesis, the strategy II Fe uptake pathway was downregulated on day 2 irrespective of Fe nutrition (Figure 5A,C and Figure S3A,C), likely because maize at that developmental stage relies more on the embryonic root system for nutrient uptake, and newly emerged CR1 roots function primarily as Fe sink tissues rather than active uptake sites. Consequently, even though the expression profile of *ZmNAS1* and *ZmYS1* is generally a well-established Fe status marker [39,40,41,42], it is probably not suitable when studying newly emerged roots at their initial developmental stages. By day 7, however, the strategy II uptake pathway was transcriptionally induced not only under Fe starvation but also in Fe-sufficient plants, as plant growth and biomass accumulation are nutritionally demanding processes, although their expression remained more robust under –Fe (Figure 5B,D and Figure S3B,D).

How GAs regulate the strategy II Fe acquisition pathway is poorly understood. In agreement with our results, Wang et al. [15] have demonstrated that the exogenous application of GAs exacerbates iron deficiency-associated phenotypes in rice, regardless of Fe supply. A closer look at the expression patterns of the key phytosiderophore synthesis genes *ZmNAS1* and *ZmDMAS1* suggests that PS biosynthesis is negatively regulated by gibberellins in maize (Figure 5A,C and Figure S3A,C). This effect of GA modulation was more pronounced shortly after treatment initiation with growth regulators (day 2), but was progressively attenuated (day 7), especially in the case of *ZmNAS1* (Figure 5B,D and Figure S3B,D). Thus, the long-term transcriptional behavior of these genes may be shaped by the interplay between GA modulation and the chronic differences in Fe nutrition. Interestingly, our in silico promoter analysis revealed that, among all strategy II genes included in this study, GA-responsive motifs are present only in the promoters of *ZmNAS1* and *ZmDMAS1*, suggesting that gibberellins may directly control these genes (Dataset S2). The mechanism by which GAs could impact the expression of these genes is not clear, given that the existence of gibberellin-responsive elements is usually associated with a positive regulation of downstream genes by GAs. Importantly, however, under Fe-deficient conditions, the endogenous levels of bioactive GAs were found to be reduced in rice, as well as in shoots of maize [15]. Declining levels of bioactive GA_4_ have also been reported in *Arabidopsis thaliana* under Fe starvation [14,15], primarily in roots [18], although GAs are known to positively regulate iron uptake in strategy I plants [14,18,57,58]. To the best of our knowledge, nevertheless, the impact of iron deficiency specifically on root GA concentrations in graminaceous species has not been addressed as of now. It should also be noted that the enzymes in the GA biosynthetic/catabolic pathway downstream of GA_12_ are Fe-dependent 2′ODDs [59,60,61], and therefore lower levels of bioactive GAs can be expected under iron deficiency, as low Fe levels could interfere with proper 2′ODD function, which might then limit the conversion of GA_12_ to bioactive GA species.

Gibberellins have been shown to suppress the root-to-shoot translocation of Fe in rice [15,16]. GAs could have a similar effect on Fe translocation in maize, as indicated by the root and shoot iron concentrations of “300G” plants, the prompt and robust upregulation of both *ZmFER* genes (day 2) likely due to the high root Fe accumulation, and the occurrence of Fe starvation in “300G” shoots leading to the overexpression of strategy II Fe uptake genes in the LR (Figure 4 and Figure 5A–C and Figure S3A,C). In this regard, we postulate that the impairment of root-to-shoot iron translocation in GA_3_-treated seedlings could be, at least partly, the result of compromised 2′-deoxymugineic acid (DMA) biosynthesis given the suppression of *ZmDMAS1* by exogenous GA, especially in the LR (Figure 5B,D and Figure S3B,D). Although DMA is chiefly involved in iron uptake in maize and other graminaceous plants, the presence of DMA-Fe^3+^ complexes has been confirmed in the xylem sap [62,63,64,65] in addition to the phloem [66], indicating that DMA participates in the translocation of Fe within the plant body. Beyond its primary role in DMA biosynthesis to mediate Fe uptake from the rhizosphere, DMAS1 may be additionally implicated in the production of DMA utilized for internal Fe translocation. In rice, for example, although *OsDMAS1* is robustly induced in several root tissues under –Fe, its expression is nevertheless limited to cells involved in long-distance transport when iron is sufficient [67], suggesting that DMAS1 is a substantial contributor among the DMAS/DMAS-L members in this regard [65]. Likewise, the root iron concentration in mepiquat-treated seedlings was reduced on day 7, while their shoot contained more Fe compared to “300G” seedlings (Figure 4). Also, the rapid and strong induction of *ZmDMAS1* seen initially in “10M” roots was maintained in the LR on day 7, or it was even further upregulated in the case of the root apex (Figure 5B,D and Figure S3B,D). Hence, it can be inferred that GA biosynthesis inhibition facilitates Fe allocation from roots towards the shoot by stimulating *ZmDMAS1* expression.

**Figure 13:**
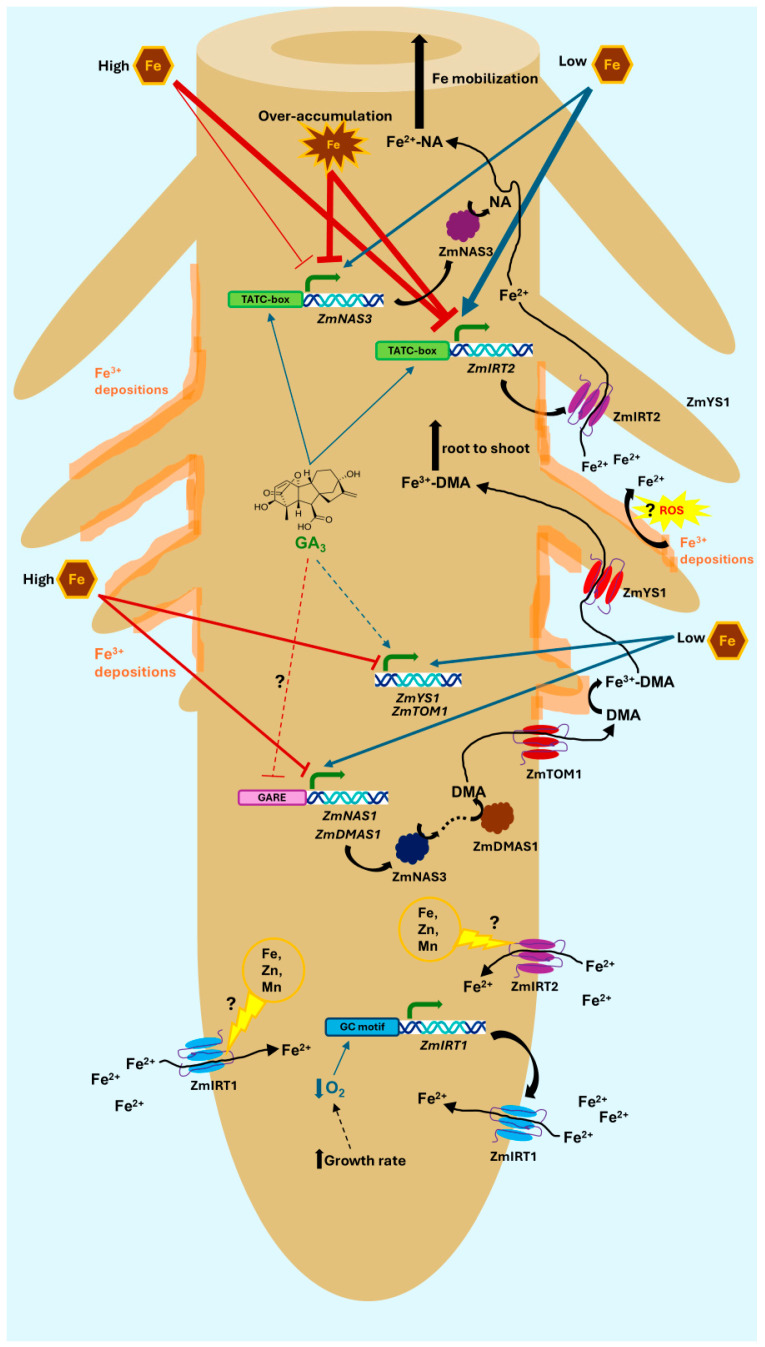
Proposed model for the modulation of Fe uptake by the interplay between gibberellins and the Fe status in maize roots. A regulatory and functional coupling of ZmNAS3 and ZmIRT2 is postulated. Low Fe levels induce *ZmNAS3* in roots, while they are a prerequisite for *ZmIRT2* expression. Under limited iron, gibberellins positively control both *ZmNAS3* and *ZmIRT2*, likely via the TATC-box found in the promoters of both genes. Iron overaccumulation in root tissues strongly suppresses *ZmNAS3*, while, under high internal or external Fe levels, *ZmIRT2* expression is undetectable in CR1 roots. This co-regulation ensures that incoming Fe^2+^ is readily and sufficiently chelated by NA to prevent iron toxicity and to facilitate Fe mobilization towards sink tissues. The depositions of Fe^3+^ present on the root surface presumably serve as an iron pool for the formation of Fe^2+^, although the exact mechanism remains elusive. Gibberellins negatively control *ZmNAS1* and *ZmDMAS1*, although the role of the GARE element found in the respective promoters is not clear. The strategy II uptake genes *ZmTOM1* and *ZmYS1* are induced by GAs in a rather indirect manner as a consequence of gibberellins’ impact on plant physiology and Fe homeostasis. Contrary to *ZmIRT2*, Fe availability and GAs do not directly control *ZmIRT1*, while its expression is influenced by root growth. We thus hypothesize that ZmIRT1 could serve as a more constitutive, developmentally modulated Fe^2+^ uptake component. The possible role of the two ZmIRT homologs as metal sensors through their histidine rich loops warrants further investigation. DNA molecule illustration created with BioRender.com.

In addition to DMA, nicotianamine (NA) is also known to be involved in long-distance Fe transport by chelating both Fe^2+^ and Fe^3+^ [68,69]. In contrast to *ZmNAS1*, which is primarily associated with phytosiderophore biosynthesis for Fe uptake [40], the NA produced by ZmNAS3 is utilized for the chelation and translocation of Fe inside the plant body, especially in developing tissues [39,42]. In rice, the expression of *OsNAS3* is almost exclusively limited to the shoot when Fe is sufficient. However, in cases of Fe deficiency, *OsNAS3* is suppressed in rice shoots but is specifically induced in companion cells and protoxylem-adjacent pericycle cells in the root [65,68,70,71,72], possibly to promote root-to-shoot Fe translocation. Our RT-PCR analysis demonstrates that, similarly to *OsNAS3*, *ZmNAS3* is induced in maize roots under prolonged Fe starvation (day 7) (Figure 5B,D and Figure S3B,D). The core promoter of *ZmNAS3* contains a TATC-box (Supplementary Dataset S2), a motif found in several gibberellin-inducible genes [73], which might explain the upregulation of *ZmNAS3* only in “300G” roots on day 2 and the blunting of its induction in the root apex of Fe-starved plants treated with mepiquat chloride on day 7 (Figure 5 and Figure S3). However, *ZmNAS3* was strongly downregulated on day 7 in both root sectors of “300G” seedlings. The suppression of NA production by *ZmNAS3* downregulation highlights a second mechanism, beyond the reduction in DMA synthesis, by which GA treatment impedes the translocation of iron towards the shoot. A possible explanation is that *ZmNAS3* is regulated by the Fe status in a similar way to *OsNAS3*, the expression of which is decreased in rice roots under Fe abundance [68,70,71,72]. In that case, the overaccumulation of Fe in “300G” roots (Figure 4) could act as a suppressive local signal, overriding the initial positive regulation of *ZmNAS3* by GAs (Figure 5A,C and Figure S3A,C). Alternatively, exogenously applied GA_3_ might interfere with endogenous GA production and distribution via negative feedback loops, ultimately reducing *ZmNAS3* expression. Collectively these results provide compelling evidence that, similarly to rice, GAs negatively control Fe root-to-shoot translocation in maize, at least partly, in a DMA-dependent manner in both the short term and long term, as well as through the mitigation of ZmNAS3-driven NA synthesis under prolonged GA_3_ treatment (Figure 13).

Gibberellins may impact strategy II Fe uptake genes indirectly, as opposed to the direct modulation of PS biosynthesis. The effects of exogenously applied GA_3_ or mepiquat chloride on the expression of *ZmTOM1* and *ZmYS1* likely stem from changes in plant physiology and Fe homeostasis resulting from altered GA dynamics. For instance, both genes are upregulated on day 7 in the LR of “300G”, reflecting the Fe-deficient conditions in the shoots of GA_3_-treated seedlings (Figure 5B and Figure S3B). On the other hand, in “10M” roots, the expression of strategy II uptake genes was stimulated in the A in response to increased root growth but not in the LR due to the blunted –Fe response in the shoot (Figure 5B,D and Figure S3B,D).

The accumulation of Fe^3+^ plaques on the root surface is determined by the chemical environment of the rhizodermal cell walls and the Fe^3+^ uptake rate, while it is also analogous to root tissue age and the external iron levels.

Ferric iron uptake was enhanced by day 7 in the LR of “300G” roots, as indicated by the reduced Fe^3+^ deposition compared to their non-GA_3_-treated counterparts (Figure 3A,C), reflecting the Fe starvation observed in the shoot of these plants. Additionally, the high iron concentration observed on day 7 (Figure 4) and the strong induction of *ZmFER1* and *ZmFER2* from early on (day 2) (Figure 5A,C and Figure S3A,C) provide evidence of a rapid increase in iron influx in roots after GA_3_ application. By contrast, the Fe^3+^ deposition score in the LR of “10M” roots was substantially higher compared to “10” plants (Figure 3C), consistent with the observation that “10M” shoots were not sensing Fe starvation, despite the low iron nutrition (10 μM Fe) and the low chlorophyll content in all four leaves (Figure 1D). The upregulation of strategy II Fe uptake genes in the apex of “10” and “10M” roots, combined with the low external Fe levels, resulted in the depletion of ferric iron plaques there (Figure 3C, Figure 5D and Figure S3D). Interestingly, however, even though *ZmYS1* was not upregulated in the A of “300G” roots, as opposed to “300” apices on day 7, Prussian blue scores were significantly lower in the former compared to the latter. This peculiarity can be attributed to the robust overexpression of *ZmTOM1* in the A of “300G” roots. In that case, the enhanced PS release would result in an excessive solubilization of the Fe^3+^ contained in the iron plaques. In agreement with our observations, a decrease in ferric iron plaque accumulation and the promotion of Fe uptake by exogenously applied GA_3_ has also been reported in rice roots [74].

### 3.3. Ferrous Iron Uptake in Maize: Evidence for a ZmIRT1- and ZmIRT2-Mediated Fe^2+^ Uptake Pathway Operating in Conjunction with Strategy II

In addition to rice, which follows a mixed strategy for iron uptake, the existence of *IRT1* homologs in the genomes of graminaceous members thought to be strictly II species (Figure S6) challenges the stringent distinction often imposed between the two Fe sequestration strategies. The high homology of the grass *IRT* homologs to their non-graminaceous counterparts points to a common ancestor gene (Figure S6), and such conclusions can also be drawn from previous studies [8]. These observations provide strong evidence in favor of the Ancient Combined Strategy model proposed for the evolution of the combined Fe uptake strategy [11]. Beyond *Oryza sativa*, evidence shows that other rice species, as well as strategy II plants including maize, likely follow a mixed strategy for iron acquisition [22,25]. Moreover, maize *ys1* mutants grown under Fe sufficiency are not lethal, but they are rather considered as Fe-inefficient, showing a relatively mild chlorotic phenotype in the form of “yellow stripes” [27,75,76,77]. Similarly, maize seedlings defective in phytosiderophore secretion (*tom1*(*ys3*) mutants) are viable, show near-wild-type (WT) growth, and have a chlorosis phenotype similar to that of *zmys1* mutants under Fe-sufficient conditions. In contrast, Fe-deficient WT maize plants are characterized by severe chlorosis [24,27]. Combined, these observations suggest that additional Fe acquisition mechanisms, independent of ZmTOM1-ZmYS1, which is the main Fe sequestration pathway under Fe starvation [77,78], plausibly exist in maize.

Rice has two functional IRT1 homologous proteins, OsIRT1 and OsIRT2, both being able to transport Fe^2+^ [79,80]. Since rice is usually cultivated in waterlogged soils, ferrous iron is the primary Fe source, rendering the existence of an Fe^2+^ acquisition system indispensable. Maize on the other hand grows in well-aerated soils where ferric iron prevails, and thus the Fe^3+^ chelating strategy guarantees an efficient Fe uptake. However, ferrous iron might constitute a significant Fe source for maize as well. Here we report that, under well-aerated hydroponic conditions with the only iron source being Fe^3+^ in the form of FeNaEDTA, significant amounts of Fe^2+^ form on the root surface of maize seedlings (Figure 3B,D). The presence of ferrous iron was detected in situ by Turnbull blue staining, which selectively reacts with Fe^2+^ to form a blue precipitate. Considering that ferrous iron is highly soluble and given that the nutrient solutions were well aerated, Fe^2+^ formation on the root surface must be a continuous process. In strategy I plants, Fe^3+^ is reduced to Fe^2+^ by FRO homologs. To test whether a similar mechanism is present in maize, we studied the expression of *ZmFRO2*, the closest homolog of *AtFRO1* in maize (Figure S7). Importantly, *ZmFRO2* transcription was undetectable in all treatments, on both sampling days. We then hypothesized that ferrous iron could be produced from Fe^3+^ iron depositions in a non-enzymatic way through the Haber–Weiss and/or the Fenton reaction [81]. Staining with KI/starch reagent [82] failed to detect H_2_O_2_ on the root surface. The superoxide anion was present exclusively in root tips in the lower part of the apex (Figure 2D), where Fe^3+^ depositions were scarce or absent. The generation of the superoxide anion there is most probably associated with root growth, as reactive oxygen species (ROS) have already been linked to this process [83,84]. Indeed, a close correlation can be seen between NBT scores and root elongation rates, especially from day 2 to day 7 (Figure 2B,D). Whether the failure to detect H_2_O_2_ and O_2_**^.^**^−^ (in parts other than the root tip) is due to their consumption from the Fenton/Haber–Weiss reactions or not remains elusive. Therefore, it is plausible that an IRT-mediated but FRO-independent Fe^2+^ uptake pathway exists in maize and possibly other strategy II plants. It has been shown that transgenic *Arabidopsis* lines overexpressing *ZmIRT1* accumulate Fe and Zn in roots and seeds [9]. Additionally, *ZmIRT1* expression in maize was found to be robustly induced in roots and shoots through Fe deficiency, while Fe excess had the opposite effect [8,85]. Our results, conversely, suggest that *ZmIRT1* transcription is only minimally influenced by the Fe nutritional status (Figure 5 and Figure S3). It should also be noted that the promoter of *ZmIRT1* contains GC-motif sequences (Dataset S2), which are often found in anaerobic responsive elements (AREs) and are associated with the transcriptional induction of control genes under anoxic conditions [86,87,88]. The coupling of *ZmIRT1* expression with hypoxia is reasonable, given that under such circumstances ferrous iron is more abundant. In line with this, RNA-Seq data [89] from the Maize Genome Database demonstrate that *ZmIRT1* is induced in maize root tips amid waterlogging. Furthermore, under normoxia, the root apical meristem is characterized by low oxygen levels as hypoxia is implicated in meristem maintenance and function [90,91]. In this context, *ZmIRT1* may be constitutively expressed for the acquisition of Fe^2+^ to satisfy the meristem’s need for iron [44]. This might explain why, on day 2, *ZmIRT1* expression was higher in the A compared to the respective LR sectors in all treatments (Figure 5A,C and Figure S3A,C). In addition to meristematic tissues, substantial oxygen consumption also occurs in root apex parts exhibiting a heightened metabolic activity, such as the elongation zone; therefore, increased root elongation rates can enhance local oxygen demands and may promote hypoxia, especially when oxygen supply from diffusion is insufficient [92]. On day 7, *ZmIRT1* was upregulated in “300” and “10M” apices but not in those of the other two treatments (Figure 5D and Figure S3D). This pattern correlates fairly well with the root elongation rates from day 2 to day 7 (Figure 2B). The enhanced elongation rate observed in “300” and “10M” apices (Figure 2B) could reflect elevated oxygen consumption [93], thereby exacerbating hypoxia, which along with the increased Fe demand from rapid growth justifies *ZmIRT1* stimulation. To avoid Fe toxicity from constitutive expression, especially in the meristem [94], ZmIRT1 could be additionally regulated at the posttranscriptional and/or posttranslational level in a comparable manner to AtIRT1 [58,95,96].

Considerably less is known about ZmIRT2. Li et al. [10] have shown that *ZmIRT2* transcription is induced in roots under Fe and Zn starvation and that the ZmIRT2 protein localizes to the plasma membrane and the endoplasmic reticulum, raising the possibility of ZmIRT2 being a functional component of Fe homeostasis in maize. Remarkably, no *ZmIRT2* transcription could be detected in “300” and “300G” roots, neither on day 2 nor on day 7 (Figure 5 and Figure S3). An in silico analysis of the core promoter region revealed the presence of a TATC-box (Supplementary Dataset S2), a motif found in several gibberellin-inducible genes [73]. Gibberellins seem to influence *ZmIRT2* expression only under Fe-insufficient conditions, but not when Fe is abundant in the growth medium. This is clearly demonstrated in Figure 5 and Figure S3 as the addition of mepiquat (treatment “10M”) generally halts the upregulation of *ZmIRT2*, as opposed to “10” roots. Conversely, the treatment of Fe-sufficient seedlings with exogenous GA_3_ fails to trigger *ZmIRT2* transcription. Taken together, these results suggest that low external Fe levels are required for *ZmIRT2* expression, and under these conditions GAs likely positively regulate *ZmIRT2*. Interestingly, in Fe-deprived plants, *ZmIRT2* was the only *IRT* homolog found to be upregulated in the short term (on day 2), and this response was evident in both root sectors (Figure 5A,C and Figure S3A,C). It has been postulated that ZmIRT2 may have a role in Fe and Zn toxicity prevention in a manner analogous to AtIRT2 in Arabidopsis [10]. In that case, *ZmIRT2* upregulation would be expected in the roots of plants growing in high Fe concentrations (300 μM Fe) and especially in GA_3_-treated seedlings (treatment “300G”) given the high root iron content (Figure 4) and the existence of the TATC-box motif in the core promoter region of the *ZmIRT2* gene (Dataset S2). Alternatively, ZmIRT2 may cooperate with ZmIRT1 to mediate ferrous iron uptake under Fe starvation but not under Fe sufficiency, where ZmIRT1 could function as a default Fe^2+^ transport module. Whether ZmIRT1 and ZmIRT2 can form homo- or heterodimers remains unknown, and further studies will be needed to elucidate such a possibility. An interesting feature of the two maize IRT homologs is the histidine-rich loop between transmembrane domains 3 and 4 (Figures S8 and S9), common in metal transporter families in various organisms and which likely serves as a metal sensing domain [8,97]. For example, AtIRT1 can detect Zn/Mn excess through a similar His-rich cytosolic loop, subsequently enabling its own degradation [98,99,100]. Whether this characteristic renders AtIRT1 a transceptor or not is still debated [101]. The possibility of ZmIRT1 and/or ZmIRT2 possessing an analogous metal sensing capability warrants further investigation.

As already mentioned, the formation of ferrous iron on the root surface combined with the expression of *ZmIRT1* and *ZmIRT2* in maize roots provides compelling evidence of an IRT-mediated Fe^2+^ acquisition pathway in maize. Importantly, *ZmIRT1* does not appear to be expressed in the root stele [102], raising the possibility that ZmIRT1 is indeed involved in Fe^2+^ uptake from the rhizosphere, although in situ localization studies are needed to confirm ZmIRT1 presence in the root epidermis and/or outer cortex layers. The amount of ferrous iron detected on the root surface is dependent on multiple factors, such as the Fe levels of the growth medium, the amount of Fe^3+^ depositions present which serve as an Fe^2+^ source, the rate of oxidation of Fe^2+^ to Fe^3+^, and the uptake of Fe^2+^ by the ZmIRTs. This complicates any attempts to correlate Fe^2+^ scores with Fe^2+^ uptake by ZmIRT proteins. For instance, Fe^2+^ scores are generally higher in “300” roots (Figure 3B,D), owing to the high external Fe levels, the increased amount of Fe^3+^ depositions (Figure 3A,C), and the absence of a strong induction of iron uptake (Figure 5 and Figure S3). On the other hand, the induction of both *ZmIRT1* and *ZmIRT2* in the LR of “10” roots (Figure 5B and Figure S3B) might account for the decreased Fe^2+^ score value observed there (Figure 3D) due to enhanced Fe^2+^ uptake. Likewise, the A of “10M” roots had significantly lower Fe^2+^ scores compared to their “10” counterparts (Figure 3D). This is expected given that in “10M” apices *ZmIRT2* was upregulated from day 0 to day 2, with no change thereafter, and *ZmIRT1* was induced from day 2 to day 7. By contrast, in the A of “10” roots, *ZmIRT1* was downregulated between day 2 and 7, and only *ZmIRT2* was overexpressed during the same timeframe (Figure 5D and Figure S3D). Plausibly, ZmIRT1 and ZmIRT2 could have complementary roles in maize as an Fe^2+^ uptake module, with *ZmIRT1* being responsive to hypoxia (reductive environment, increased abundance of Fe^2+^ as a source of iron) and *ZmIRT2* being expressed only upon low external Fe levels. A proposed model for the modulation of Fe uptake through the interplay between gibberellins and the Fe status in maize roots is depicted in Figure 13.

### 3.4. Transcriptional Regulation of Iron Deficiency Responses in the Lateral Root (LR) and Apex (A) Sectors of Maize Roots

Two IDEF1 (ZmIDEF1.1, ZmIDEF1.2) and two IDEF2 (ZmIDEF2.1, ZmIDEF2.2) homologous proteins are found in maize (Figure 6 and Figure 7). In rice, *OsIDEF1* and *OsIDEF2* are thought to be constitutively expressed regardless of Fe nutrition [2], a feature largely attributable to their function as iron sensors by directly binding Fe and to their position upstream of other key –Fe response regulators like IRO2s [103]. However, variations in the expression of both genes were observed in several cases on days 2 and 7, indicating that *ZmIDEF1.1* and *ZmIDEF2.1* could be differentially expressed according to growth stage and/or root sector (Figure 11 and Figure S4). In rice, OsIDEF1 promotes the expression of several Fe uptake and utilization genes, such as *OsIRO2*, *OsIRT1*, *OsNAS1*, *OsNAS2*, *OsNAS3*, and *OsYSL2*, not only under iron deficiency but also when iron is ample [2,16,104,105]. The expression of *ZmIDEF1.1* was positively correlated with that of *ZmIRO2.1*, *ZmIRO2.2*, the PS biosynthesis genes *ZmNAS1* and *ZmDMAS1*, and *ZmIRT2* in the fully developed LR (day 7) of Fe-sufficient (300 μM Fe) and Fe-deprived (10 μM Fe) seedlings. In the A, however, a similar trend was evident only for *ZmIRO2.1*, *ZmIRO2.2*, *ZmIRT2*, and additionally also for *ZmNAS3* (Figure 5, Figure 11 and Figures S3 and S4). Even though decisive regulatory relationships and interactions cannot be inferred from correlations in expression patterns alone, these results could indicate that ZmIDEF1.1 differentially controls downstream genes in a root sector-specific manner and that such differences are probably masked when studying whole roots. The target genes of ZmIDEF1 homologs might shift at subsequent stages of iron deprivation. In rice, for example, OsIDEF1 positively regulates *OsIRO2* as well as Fe uptake and translocation genes via the *IDE1* element under Fe-sufficient conditions and during the early stages of Fe deficiency, but under prolonged Fe starvation OsIDEF1 shifts its downstream genes by preferentially binding to the *RY* element (5′-CATGCA-3′) [16]. Notably, according to the PlantCARE database, the *RY* element is present in the core promoter region of *ZmTOM1*, *ZmNAS3*, and *ZmFER2*, although 5′-CATGCA-3′ motif repeats were also found in the promoters of *ZmIRT1*, *ZmIRT2*, *ZmIDEF2.1*, *ZmIRO2.1*, *ZmIRO2.2*, and *ZmIRO3* after a manual search (Dataset S2). On the other hand, a closer look at the expression profiles of *ZmIDEF1.1*, *ZmIDEF2.1*, *ZmIRO2.1*, *ZmIRO2.2*, and *ZmIRO3* collectively reveals that, in conditions of low Fe supply (treatments “10” and “10M”) or in settings that mimic an Fe-deficient state (“300G” seedlings), the upregulation of *ZmIDEF2.1* in both root sectors coincides with the induction of *ZmIRO2.1*, *ZmIRO2.2*, and *ZmIRO3*, irrespective of *ZmIDEF1.1* expression. This association was not observed in the roots of iron-sufficient plants (Figure 11 and Figure S4). These results, along with the existence of *IDE2 cis*-element sequences in the promoters of all three of *ZmIRO2.1*, *ZmIRO2.2*, and *ZmIRO3*, indicate that these genes could be directly regulated by ZmIDEF2.1 under –Fe (Dataset S2).

Iron-related bHLH transcription factor 2 (IRO2) is a grass bHLH transcription factor which is strongly induced under Fe deficiency and positively regulates the –Fe response. For the first time, it is demonstrated that maize possesses two IRO2 homologous proteins, ZmIRO2.1 and ZmIRO2.2 (Figure 8). According to Nozoye et al. [27], *GRMZM2G057413* (which corresponds to *ZmIRO2.1*) is upregulated in Fe-deficient maize roots.

The bHLH protein OsIRO3 (Iron-related bHLH transcription factor 3) is reportedly the ortholog of AtPYE in rice [2], while ZmIRO3 and BdIRO3 are the AtPYE orthologous proteins in maize and *Brachypodium distachyon*, respectively (Figure 9). In agreement with previous studies [27,106], Fe limitation (10 μM Fe) stimulated *ZmIRO3* across CR1 roots shortly after treatment initiation (day2), irrespective of GA inhibition by mepiquat chloride (Figure 11A,C and Figure S4A,C). This induction was attenuated at subsequent stages of –Fe, suggesting that *ZmIRO3* is an early response component, at least at the transcriptional level (Figure 11B and Figure S4B). The presence of *IDE1*, *RY*, and *IDE2* sequences in the promoter of *ZmIRO3* (Dataset S2) implies that its induction under –Fe is mediated, at least partly, by the ZmIDEF1 and/or ZmIDEF2 paralogs. Remarkably, although the newly identified ZmIRO3-like in maize and BdIRO3-like in *Brachypodium* display a high homology to IRO3 proteins from these species, they are not IRO3 paralogs, and they rather form a distinct subgroup within the IRO3/IRO3-like clade (Figure 9). Recent studies have demonstrated that the grass FIT homologs have a role in the regulation of iron homeostasis in strategy II plants like rice [107] and maize [13]. In these plant species, FIT is speculated to facilitate the localization of IRO2 to the nucleus [13,107]. Maize was found to have two FIT homologous proteins, ZmFIT1 and ZmFIT2 (Figure 10). However, no *ZmFIT1* expression could be detected. Given that the expression patterns of *ZmFIT2* and its target genes were generally not sufficiently correlated (Figure 5, Figure 11 and Figures S3 and S4A), it is possible that GAs modulate ZmFIT primarily at the posttraslational level in an analogous manner to the DELLA-FIT interaction found in *Arabidopsis* [14], although this remains a hypothesis.

Overall, while all the Fe homeostasis regulator genes studied, except *ZmIDEF1.1*, were simultaneously upregulated exclusively in the LR of “10” roots on day 7, a similar response was observed earlier (day 2) in the LR of mepiquat treated seedlings (Figure 11 and Figure S4). A comparable expression profile was maintained on day 7 in the A of mepiquat-treated roots. This “priming” effect of mepiquat chloride, regarding the prompt induction of the –Fe response under iron limitation, warrants further investigation, as it may prove to be a useful practice for enhancing low-Fe tolerance and improving the FeUE of crops.

## 4. Materials and Methods

### 4.1. Plant Material, Growth Conditions, and Treatments

**Figure 14:**
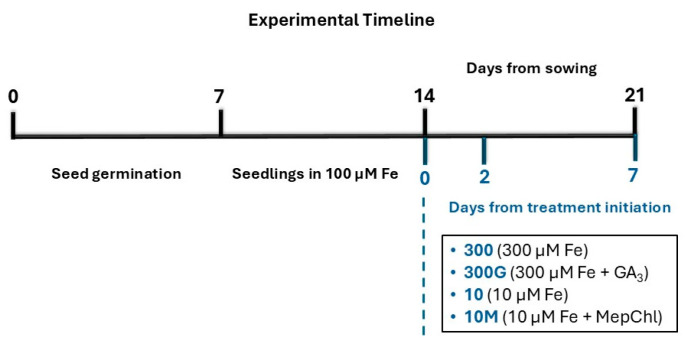
Schematic illustration of the experimental timeline.

Maize seeds (*Zea mays* L., Corteva Agriscience P0937) were germinated on wet filter paper (Figure S10A), and after four days a uniform subset of plants was selected and placed in a hydroponic batch culture for 3 days in well-aerated distilled water (Figure S10B). Seven days after sowing, the plants were transferred in a second hydroponic batch culture (Figure S10C,D), containing a complete nutrient solution which consisted of 5 mM KNO_3_, 1 mM KH_2_PO_4_, 2 mM Mg(NO_3_)_2_ 6H_2_O, 2.5 mM CaSO_4_ 2H_2_O, 1 mM MgSO_4_ 7H_2_O, 4 mM Ca(NO_3_)_2_ 4H_2_O, 0.9 μM ZnCl_2_, 30 μM H_3_BO_3_, 0.9 μM CuCl_2_, 0.5 μM MoO_3_ 85%, and 20 μM MnCl_2_ 4H_2_O and 100 μM Fe (as FeNaEDTA). A week later, the plants were divided into four groups (day 0), marking the initiation of treatments (Figure S10E). The seedlings were kept under these conditions for a total of 7 days (Figure S10F). For all four groups, the nutrient concentrations were the same as mentioned above, apart from iron, for which two different concentration levels were applied. Thus, half of the groups were grown under iron sufficiency (300 μM Fe), while the other half were kept under low-iron conditions (10 μM Fe). The above iron concentrations were selected according to Benke et al. [20]. At the same time, gibberellic acid, in a final concentration of 12 ppm of GA_3_, was added to one group of the plants grown under 300 μM Fe (treatment 300G). Likewise, mepiquat chloride (a gibberellin biosynthesis inhibitor) was applied in a final concentration of 15 ppm to one plant group grown under 10 μM Fe (treatment 10M). The rest of the plants served as controls for iron sufficiency (treatment 300) and iron insufficiency (treatment 10) (Figure 14). All nutrient solutions were constantly aerated and were replaced every 3 days throughout the experiment. Growth chamber conditions were 24/18 °C, relative humidity 40%, 250 μmol photon m^−2^ s^−1^, and a 16 h photoperiod. All chemicals were purchased by Merck KGaA, Darmstadt, Germany.

### 4.2. Plant Samplings

Each experiment was repeated twice, and samples (at least three biological replicates per treatment and sampling day) were taken both on day 0 (prior to treatment initiation), as well as on days 2 and/or 7 after treatment initiation. Samplings were performed 3 h after switching the light sources on.

### 4.3. Shoot and Root Dry Weight

Shoot and root dry weight measurements, as well as the respective shoot/root dry weight ratio calculation, were performed on day 7 after treatment initiation. The plants were harvested, and they were subsequently dissected to separate the shoot from the root system. The two plant parts were dried in an oven at 80 °C until constant weight, and the respective dry weights were then measured and the shoot/root ratio was accordingly calculated.

### 4.4. Determination of Total Chlorophyl Content

Leaf blades of the maize plants were excised on day 7 after the treatments’ initiation. Total chlorophyll content of leaves was estimated as described in [108]. Briefly, tissue samples weighting 0.05 g were taken from the central part of the leaf blade, and they were then cut into small pieces before being placed in tubes containing 5 mL DMSO. The tubes were incubated at 65 °C in a water bath for 15 min. Photometric measurements were carried out at 665 nm and 649 nm. The formula used to determine total chlorophyll content was the following:

Chl_total_ = (5.97 × A_665_) + (21.44 × A_649_)

### 4.5. Crown Root Phenotype

The length of all first-row crown roots (CR1) from a total of three seedlings was measured on day 0, before the plants were transferred into separate containers for treatment initiation. Likewise, all CRs from three plants of each treatment (300, 10, 300G, 10M) were obtained, and their length was measured both on day 2 and on day 7. Subsequently, to estimate the elongation rate (cm/day) for every treatment from day 0 to day 2, the difference in root length (cm) from day 0 to day 2 was estimated, which was then divided by the number of days between samplings. Correspondingly, the same procedure was followed to calculate the CR1 elongation rate from day 2 to day 7.

Moreover, each CR1 was divided into two parts/sectors: the sector carrying the lateral roots (LR sector) and the apex sector of the root, where no lateral root primordia could be seen under a stereoscope (A sector). The length of the A sector was also measured on the same samplings time points.

The crown roots of row 1 (CR1) were selected for two main reasons: (a) crown roots are part of the post-embryonic root system, and (b) these roots had developed after treatment initiation.

### 4.6. In Situ Superoxide Anion (O_2_**^.^**^−^) Detection on the Surface of Root Apices—Nitroblue Tetrazolium (NBT) Staining

For the in situ detection of O_2_**^.^**^−^ formation on the root apex surface of maize seedlings, entire CR1 roots were placed for 20 min in a freshly prepared 0.25 mM Nitroblue Tetrazolium solution (NBT), as described in [82]. The roots were then transferred on grid paper so that the length (cm) of the stained part of the roots could be measured. For each treatment, all CR1 roots of three plants were harvested, both on day 2 and on day 7. Since the pattern of the stained area observed in the root apices was delicate and arranged in zones of varying length and of different stain intensities, the mere summing of the length of stained parts would not be a suitable method to quantify the formation of superoxide anions on the root surface. Hence, an ordinal method [109] was deployed in which the NBT stained regions were categorized into three different groups according to stain intensity (LS: lightly stained, MS: moderately stained, DS: deeply stained). For each group, a coefficient (C) was determined ranging from 1 to 3, with 3 corresponding to zones stained more deeply (C3), while a coefficient value of 1 was related to the areas of lighter coloration (C1). Likewise, parts of the root surface moderately stained were given a coefficient value of 2 (C2). Subsequently, for the score value determination of each of the three zones (LS, MS, DS), in every root apex, the length of each zone was multiplied with the corresponding coefficient individually, as follows:

NBT score = LS × C1 + MS × C2 + DS × C3

where LS corresponds to the sum of lightly stained zones (cm), MS corresponds to the sum of moderately stained zones (cm), and DS corresponds to the sum of deeply stained zones (cm).

### 4.7. In Situ Detection of Ferric Iron (Fe^3+^) Deposition and Ferrous Iron (Fe^2+^) Formation on the Root Surface

While insoluble Fe^3+^ depositions are present on roots, such depositions of Fe^2+^ do not exist, since ferrous iron is highly soluble in the growth medium. In any case, in order to detect and quantify the levels of both iron forms in situ on roots, the formation of insoluble complexes of ferric and ferrous iron is necessary via the use of suitable compounds. Hence, in the present study, Prussian blue and Turnbull blue were chosen to complex ferric iron (Fe^3+^) and ferrous iron (Fe^2+^), respectively, in situ, on the root surface of maize seedlings. All CR1 roots of three seedlings were obtained for each treatment both on day 2 and on day 7. Intact CR1 roots were then placed for 20 min in freshly prepared Prussian blue solution. The same process was repeated for Turnbull blue staining, and therefore intact CR1 roots were incubated for 20 min in freshly prepared Turnbull blue solution [110]. Since staining intensity was highly variable in both cases, the mere summing of the length of stained parts would not be a suitable method to quantify Fe^3+^ depositions and the formation of Fe^2+^ on the root surface. Hence, an ordinal method [109] was deployed in which Prussian blue or Turnbull blue stained regions were categorized into three different groups according to stain intensity. For each group, a coefficient (C) was determined with a value ranging from 1 to 3, with 3 corresponding to zones stained more deeply (C3), while a coefficient value of 1 was used for lightly colored zones (C1). Likewise, parts of the root surface moderately stained were given a coefficient value of 2 (C2). Firstly, for each root sector (LR or A), the total length (cm) of parts stained either lightly (light blue, LB), moderately (blue, B), or deeply (deep blue, DB) was calculated. Subsequently, each of these sums was multiplied by the corresponding coefficient, and the multiplication product was then divided by the total length (cm) of the respective root sector (S: LR or A) to account for variations in root sector length. Finally, the three different scores obtained for every staining intensity (score LB, score B, score DB) were summed to determine the score of either Fe^3+^ (Prussian blue staining) or Fe^2+^ (Turnbull blue staining) for every CR1 root sampled in every treatment, both on days 2 and 7.

The same formulas were utilized to determine Fe^3+^ (Prussian blue) or Fe^2+^ (Turnbull blue) scores, and are provided below:

$${\mathrm{Fe}}^{3+}\;\mathrm{score}\;\mathrm{or}\;{\mathrm{Fe}}^{2+}\;\mathrm{score}\;=\;\frac{LB\times C1\;+\;B\times C2\;+\;DB\times C3}{S}$$

where S corresponds to the length (cm) of the respective root sector (LR or A).

### 4.8. Root and Shoot Iron Concentrations

The root and shoot iron concentrations were determined as described in [39].

### 4.9. In Silico Analysis

#### 4.9.1. Phylogenetic Tree Construction

All sequence alignments were carried out with the T-Coffee database (https://tcoffee.crg.eu/). MEGA11 was leveraged for the evolutionary analyses and the generation of phylogenetic trees [111]. The evolutionary history was inferred by using the maximum likelihood method and JTT matrix-based model [112]. The tree with the highest log likelihood is shown. Initial tree(s) for the heuristic search were obtained automatically by applying Neighbor-Join and BioNJ algorithms to a matrix of pairwise distances estimated using the JTT model, and then by selecting the topology with a superior log likelihood value. The tree is drawn to scale, with branch lengths measured in the number of substitutions per site (below the branches).

#### 4.9.2. *cis*-Element Prediction in Gene Promoters

The PlantCARE database (http://bioinformatics.psb.ugent.be/webtools/plantcare/html/ (accessed on 10 October 2025)) was employed to scan the core promoters of selected genes to identify putative *cis*-regulatory elements based on sequence similarity to curated motifs. This analysis provides a descriptive annotation of predicted *cis*-elements and does not involve *p*-value-based statistical significance testing. Core promoter sequences (proximal 1000 bp, upstream of the TSS site) were obtained from the JBrowse Database (https://jbrowse.maizegdb.org/) by clicking on “Assembly” and picking “Predicted core promoter elements within 5′ 1000 bp promoter regions (MaizeGDB 2022)” [113].

#### 4.9.3. Gene Expression Analysis

Gene expression patterns for the selected genes were studied using RT-PCR as previously described [39]. Root samples were taken on day 0 prior to treatment initiation, as well as on days 2 and 7 after treatment initiation. Samples were taken from either the apex sector (proximal to the root tip) or the lateral root sector of CR1 roots. Plant tissue was immediately submerged in liquid nitrogen and stored at −80 °C. Then, the root samples were homogenized using liquid nitrogen, and nucleic acid extraction was performed using the Phenol–Chloroform protocol [114]. Genomic DNA was subsequently removed with recombinant DNase I (Rnase-free) (Takara Bio Inc., Kusatsu, Shiga, Japan), and reverse transcription was carried out with PrimeScript RT reagent (Perfect Real Time, Takara Bio Inc.). For the determination of nucleic acid concentrations, the Quawell Q5000 UV-Vis Spectrophotometer (Quawell Technology, Inc., San Jose, CA, USA) was used. For the RT-PCR, the KAPA SYBR FAST Master Mix (KAPA Biosystems, Wilmington, MA, USA) was selected, and all reactions were performed using a QuantStudio 3 Real-Time PCR System thermocycler (Applied Biosystems, Foster City, CA, USA). All oligonucleotide primers used are listed in Table S3 (primers were ordered from Eurofins Genomics, Ebersberg, Germany). The gene of ubiquitin was used as an internal control, and the target was detected using the following primer pair: ZmUBQ (MaizeGDB Gene ID: Zm00001eb369510): forward 5′-TGTCTTCATGGCCAACCACT-3′ and reverse 5′-GCTTGATAGGTAGGCGGGTG-3′ [39].

The LinRegPCR software (Version: 2021.2) was used to determine the efficiency for each RT-PCR [115]. For the calculation of the relative expression ratios of the genes studied, the following formula was utilized [116]:

$$\mathrm{Ratio}\;=\;\frac{Etar^{\Delta CP\;tar\left(control-sample\right)}}{Eref^{\Delta CP\;ref\left(control-sample\right)}}$$

where Etar is the real-time PCR efficiency of the target gene transcript, Eref is the real-time PCR efficiency of the reference gene transcript, ∆CPtar is the CP deviation of control–sample of the target gene transcript, and ∆CPref is the CP deviation of control–sample of the reference gene transcript [116].

For day 2, day 0 was used as the control, while day 2 was selected as the sample. Similarly, on day 7, day 2 was leveraged as the control, while day 7 was used as the sample.

Ubiquitin (ZmUBQ) was selected as the reference gene [39]. For every treatment (“300”, “300G”, “10”, “10M”), day 0 served as the control for day 2, whereas day 2 was used as the control for day 7.

### 4.10. Statistical Analysis

All data (means ± SE) were obtained from at least three biological replicate experiments. Data were analyzed using the *t*-test variance analysis with two-tailed distribution and two-sample unequal variance in Microsoft Excel. Where deemed necessary, Tukey’s multiple comparison test was performed.

## 5. Conclusions

Taken together, our results indicate that gibberellins are important contributors to iron homeostasis in maize by controlling the development of iron deficiency-associated phenotypes, as well as by regulating Fe uptake and translocation pathways and root sector-specific responses. The application of exogenous GA_3_ under Fe sufficiency reproduced hallmark –Fe symptoms, including the significant reduction in shoot biomass, the altered shoot/root dry weight ratio, the occurrence of chlorosis primarily in younger leaves, the marked inhibition of root elongation, and the emergence of the BTR phenotype. Conversely, the inhibition of GA biosynthesis by mepiquat chloride under low iron nutrition attenuated these typical –Fe manifestations. These phenotypic outcomes were accompanied by distinct transcriptional signatures in the lateral root zone (LR) and apex (A), supporting a model in which the LR predominantly reflects shoot Fe status via long-distance signaling, while the apex integrates systemic signals with local Fe demand, driven by root growth. At the molecular level, our data indicate that GAs negatively affect phytosiderophore biosynthesis through the suppression of *ZmNAS1* and *ZmDMAS1* and are consistent with GAs impairing root-to-shoot Fe translocation by limiting DMA and ZmNAS3-derived NA production, thereby promoting root Fe accumulation and contributing to an –Fe response in the shoot. On the other hand, GA biosynthesis inhibition sustained *ZmDMAS1* and *ZmNAS3* expression under Fe limitation and seems to have promoted Fe allocation to the shoot. In parallel, the in situ detection of Fe^2+^ on the root surface, combined with the expression patterns of the two maize *IRT* paralogs, *ZmIRT1* and *ZmIRT2*, observed in both root sectors regardless of the Fe status (*ZmIRT1*) or exclusively under low external iron levels (*ZmIRT2*), provides evidence for the existence of an IRT-mediated, FRO-independent Fe^2+^ uptake mechanism operating alongside strategy II in maize. Finally, the concomitant upregulation of known and novel maize *IDEF1*, *IDEF2*, *IRO2*, and *IRO3* homologs under either low Fe or iron sufficiency combined with GA_3_ treatment, together with the earlier transcriptional induction of these regulators in “10M” roots, indicates that GAs may affect the magnitude, spatial organization, and timing of the transcriptional –Fe response in maize. Collectively, these findings identify GA signaling as a key regulator of Fe homeostasis in maize, with potential relevance for improving FeUE in graminaceous crops.

## Data Availability

The data presented in this study are available on request from the corresponding author. The data are not publicly available due to privacy or restrictions.

## Supplementary Materials

The following supporting information can be downloaded at https://www.mdpi.com/article/10.3390/ijms27031323/s1.

## Abbreviations

The following abbreviations are used in this manuscript:

BTR	Branching at the Terminal 5 cm of the Root
FeUE	Iron Use Efficiency
GAs	Gibberellins
PS	Phytosiderophore
