# Supplementary material for: Expanding Iron Acquisition in Maize: Root Sector-Specific Responses and Gibberellin Regulation of Ferric and Ferrous Iron Uptake
Source: Int J Mol Sci. 2026 Jan 28;27(3):1323. doi: 10.3390/ijms27031323 (PMC12897795; doi:10.3390/ijms27031323)
Supplement: Supplementary file 1 [file ijms-27-01323-s001.zip › ijms-4108281-supplementary.pdf]

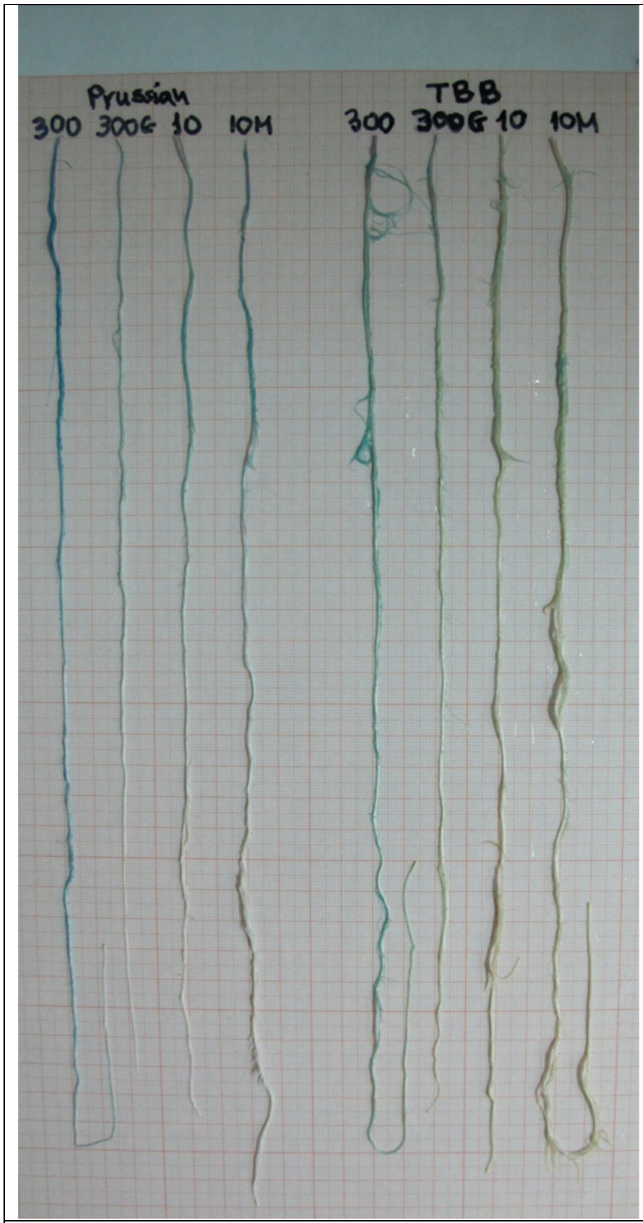

Figure S1. A: Prussian blue staining for Fe^{3+} depositions of first-row crown roots (CR1) on day 7. From left to right: 300, 300G, 10, 10M. B: Turnbull blue staining for Fe^{2+} formation of first-row crown roots (CR1) on day 7. From left to right: 300, 300G, 10, 10M. The precise distinction of the LR and A sectors as well as the determination of zones of differing staining intensity are not possible macroscopically, and were both carried out under a stereoscope. 300: maize seedlings grown in iron sufficient conditions (300μM Fe). 300G: maize seedlings grown under iron sufficiency (300μM Fe) in the presence of exogenous gibberellic acid (G). 10: maize seedlings grown in iron insufficient conditions (10μM Fe). 10M: maize seedlings grown under iron insufficiency (10μM Fe) but with the addition of the gibberellin biosynthesis inhibitor Mepiquat Chloride (M) in the growth medium.

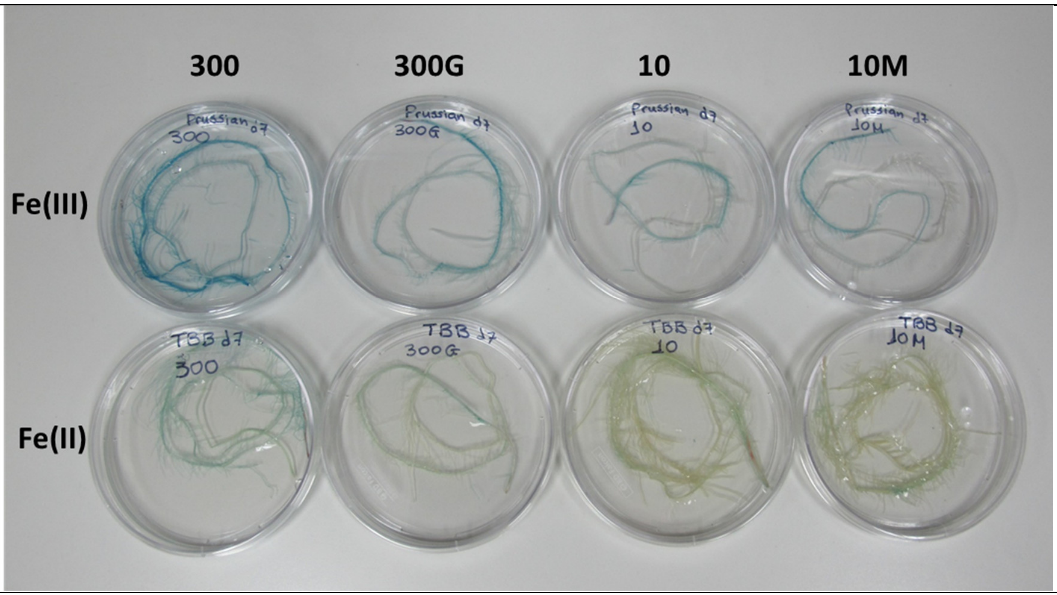

Figure S2. Petri dishes containing first-row crown roots (CR1) stained with either Prussian blue [Fe(III)] or Turnbull blue [Fe(II)] on day 7. 300: maize seedlings grown in iron sufficient conditions (300μM Fe). 300G: maize seedlings grown under iron sufficiency (300μM Fe) in the presence of exogenous gibberellic acid (G). 10: maize seedlings grown in iron insufficient conditions (10μM Fe). 10M: maize seedlings grown under iron insufficiency (10μM Fe) but with the addition of the gibberellin biosynthesis inhibitor Mepiquat Chloride (M) in the growth medium.

Table S1. List of Zea mays genes included in the phylogenetic trees. For each gene the following information is given: MGDB ID, common name, name cited by the MGDB, number of different transcripts, canonical protein number as cataloged in the MGDB, number of amino acids per canonical protein product, and chromosome number.

Annotation/ MGDB ID (Assembly version: Zm-B73-REFERENCE-NAM-5.0)	Name	MGDB name	Nº of transcripts	Canonical transcript/ protein (according to MGDB)	Nº of amino acids (a.a.) (Canonical protein)	Chromosome
Zm00001eb052440	ZmIRT1	IRT1	3	P003	381	1
Zm00001eb065410	ZmIRT2	IRT2	1	P001	361	1
Zm00001eb081810	ZmFRO2	FRO2	1	P001	760	2
Zm00001eb143690	ZmVP1	VP1	2	P001	691	3
Zm00001eb361390	ZmLFL1	ABI19	3	P001	392	8
Zm00001eb198710	ZmIDEF1.1	ABI47	2	P002	369	4
Zm00001eb259870	ZmIDEF1.2	ABI49	4	P002	322	6
Zm00001eb288420	ZmIDEF2.1	NACTF112	1	P001	452	6
Zm00001eb349010	ZmIDEF2.2	NACTF38	2	P001	445	8
Zm00001eb076630	ZmNAC82	RAS18A1	1	P001	218	2
Zm00001eb140680	ZmIRO2.1	bHLH126	1	P001	237	3
Zm00001eb362800	ZmIRO2.2	bHLH54	1	P001	246	8
Zm00001eb018460	ZmIRO3	bHLH185	6	P004	268	1
Zm00001eb328250	ZmIRO3-like	bHLH190	3	P003	266	7
Zm00001eb167310	ZmbHLH47	bHLH187	3	P003	274	4
Zm00001eb159750	ZmbHLH121	bHLH166	1	P001	316	3
Zm00001eb085690	ZmFIT1	bHLH101	2	P001	399	2
Zm00001eb420910	ZmFIT2	bHLH100	2	P001	381	10

Table S2. List of genes from *Arabidopsis thaliana*, *Oryza sativa*, and *Brachypodium distachyon* included in the phylogenetic trees. The name, NCBI annotation ID, length of their products (in amino acids), and the chromosome in which they are found is given for each gene below.

Annotation/ NCBI ID	Name	Nº of amino acids (a.a.)	Chromosome
Arabidopsis thaliana			
NP_567590.3	AtIRT1	347	4
NP_001031670.1	AtIRT2	350	4
NP_171665.1	AtFRO1	704	1
NP_171664.1	AtFRO2	725	1
NP_001319065.1	AtFRO3	716	1
NP_197786.2	AtFRO4	699	5
NP_001330174.1	AtFRO5	699	5
NP_199784.2	AtFRO6	738	5
NP_199785.2	AtFRO7	747	5
NP_199827.2	AtFRO8	728	5
NP_189108.1	AtABI3	720	3
NP_564304.1	AtLEC2	363	1
NP_566799.1	AtFUSCA3	313	3
NP_001078551.1	AtNAC82	489	5
NP_201211.1	AtNAC103	356	5
NP_191256.1	AtbHLH38	253	3
NP_191257.1	AtbHLH39	258	3
NP_181657.1	AtbHLH100	242	2
NP_001330787.1	AtbHLH101	240	5
NP_001190029.1	AtPYE	240	3
NP_001329776.1	AtbHLH11	287	4
NP_001030729.1	AtbHLH121	337	3
NP_850114.1	AtFIT	318	2
Oryza sativa			
NP_001405326.1	OsIRT1	374	3
NP_001405324.1	OsIRT2	370	3
NP_001406286.1	OsFRO1	758	4
XP_025878207.1	OsVP1	729	1
NP_001384109.1	OsLFL1	402	1
NP_001409295.1	OsIDEF1	362	8
XP_025880492.1	OsIDEF1-like1	438	4
NP_001406901.1	OsIDEF1-like2	433	4
XP_015639254.1	OsIDEF2	449	5
XP_066165793.1	OsNAC82	400	4
XP_015612709.1	OsIRO2	248	1
NP_001404728.1	OsIRO3	252	3
XP_015617819.1	OsbHLH47 (OsbHLH062-like)	278	11
XP_015626280.1	OsbHLH121	343	2
XP_015634338.1	OsFIT	352	4

Brachypodium distachyon			
XP_014752016.1	BdIRT1	367	1
XP_014751254.1	BdFRO1	728	5
XP_003579831.1	BdFRO2 (BdFRO7??)	750	5
XP_010232722.1	BdVP1	697	2
XP_014754315.1	BdLFL1	299	2
XP_003573243.1	BdIDEF1	349	3
XP_003579476.4	BdIDEF1-like	458	5
XP_003568407.1	BdIDEF2 (BdNAC74)	464	2
XP_003581627.1	BdNAC82 (BdNAC102)	390	5
XP_003565088.1	BdIRO2.1a	242	2
XP_003565095.1	BdIRO2.1b	242	2
XP_024317684.1	BdIRO2.2	266	3
XP_003557817.1	BdIRO3 (BdbHLH062)	263	1
XP_003562634.1	BdIRO3-like (BdbHLH062)	265	1
XP_010237571.1	BdbHLH47 (BdbHLH062)	272	4
XP_024317977.1	BdbHLH121	333	3
XP_010239803.2	BdFIT	356	5

Table S3. The list of maize genes studied and the respective primer pairs used for qPCR

Gene name	Chromosome	Gene ID (Maize Genome Database)	Forward primer (5’→3’)	Reverse primer (5’→3’)
ZmYS1	5	Zm00001eb249020	GTCTTCCATTCTCGCTCTGG	CAACCAACCACAGTTGATGC
ZmTOM1	3	Zm00001eb133440	TGCAGAACTATGCTGTGCCA	GCATCTTGGCGTTTTTGGGT
ZmDMAS1	1	Zm00001eb010040	AAGTCCAAGGGCAAGACCG	AGTCCACGATGTCCAGGTTC
ZmNAS1	9	Zm00001eb396230	GGAACTTTTGAGCACCTATGCG	CACTTCACAATGCATAGCATCGAAT
ZmNAS3	1	Zm00001eb052890	CGTGTCTACACCACATGCGT	TCGGACTTCGACTTCTACCCT
ZmIDEF1.1	4	Zm00001eb198710	TCCGTGGGGAGAAGTCCATT	TATGGTGTATCCTCGGACGC
ZmIDEF2.1	6	Zm00001eb288420	TGGATTTCGTTTCCACCCAAC	CGAAGAGAGGACTTTTCAGGGAG
ZmIRO2.1	3	Zm00001eb140680	TCCACCTTTGGGAACAAGACA	GAAGGAAACGGCAGGAACCAT
ZmIRO2.2	8	Zm00001eb362800	TCTGCGAGCAACTGGAGAAG	GTAGCACGCAGAGGAATGGT
ZmIRO3	1	Zm00001eb018460	ATGCCGAAGCTATGGTCTGG	ACATTCTGTTTTGATGGCCACTG
ZmIRT1	1	Zm00001eb052440	CCAAGTGGGCGTGATCGTAT	TGCAGGCAGTGTTGGTGTAA
ZmIRT2	1	Zm00001eb065410	CCAAGTGGGCGTAGCTTCAT	AGCGTGAGCAAACACAGAGA
ZmFRO2	2	Zm00001eb081810	GTACGTCTCGTTTGGTCGGT	ACTATCGTCTGTGCGTGTGT
ZmFIT1	2	Zm00001eb085690	TTTGGATGATGGCGGCTCTT	CCCCTCGAAACACTCAGCAA
ZmFIT2	10	Zm00001eb420910	TCTCCGCTTCTACTCCCTGT	CGGATGAACGGTCGAGATCA
ZmFER1	4	Zm00001eb195010	GTTGATCAGGCGGAGGAATA	AATCCCAACGAGCATAGCAC
ZmFER2	10	Zm00001eb404870	TGAGCTCTGGGTGGTGTATTC	CCTCCACGTAACATCCATCA

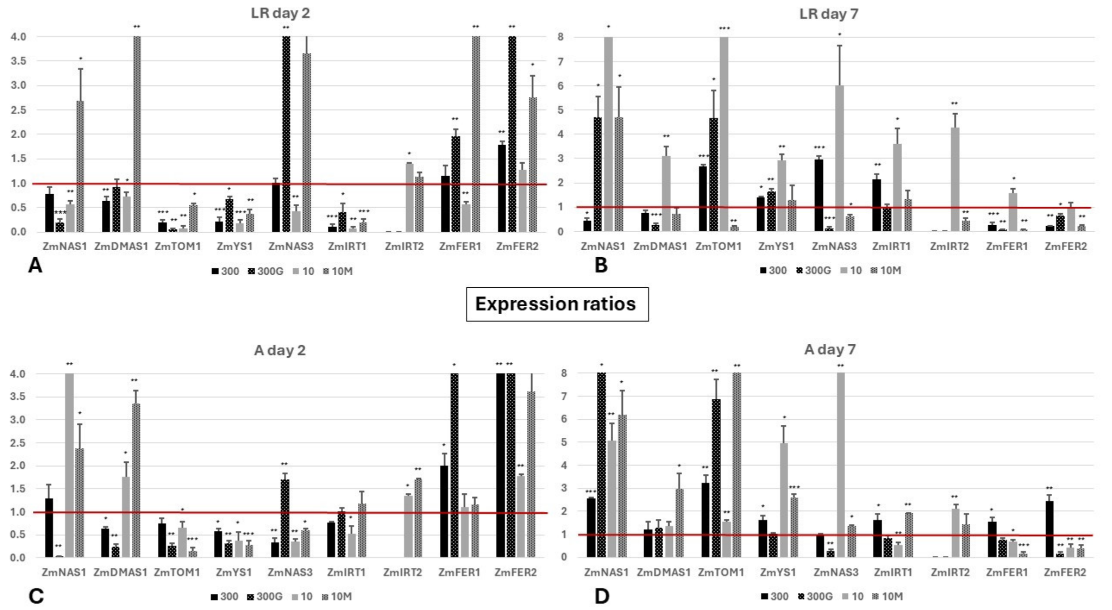

Figure S3. Detailed depiction of gene expression ratios of Figure **5** at low ratio values. Gene expression ratios of strategy II (*ZmNAS1*, *ZmDMAS1*, *ZmTOM1*, *ZmYS1*) and strategy I (*ZmIRT1*, *ZmIRT2*) genes, as well as of the nicotianamine (NA) synthase gene *ZmNAS3* and ferritin genes *ZmFER1* and *ZmFER2* in the LR (A,B) and A (C,D) in the CR1 roots of maize seedlings on day 2 and on day 7. 300: maize seedlings grown in iron-sufficient conditions (300 μM Fe). 300G: maize seedlings grown under iron sufficiency (300 μM Fe) in the presence of exogenous gibberellic acid (G). 10: maize seedlings grown in iron-insufficient conditions (10 μM Fe). 10M: maize seedlings grown under iron insufficiency (10 μM Fe) but with the addition of the gibberellin biosynthesis inhibitor mepiquat chloride (M) in the growth medium. LR: lateral root sector, A: root apex, CR1: first row of crown roots. Mean ± SE. Asterisks indicate statistically significant overexpression or downregulation. $p < 0.05$ (*), $p < 0.01$ (**), $p < 0.001$ (***).

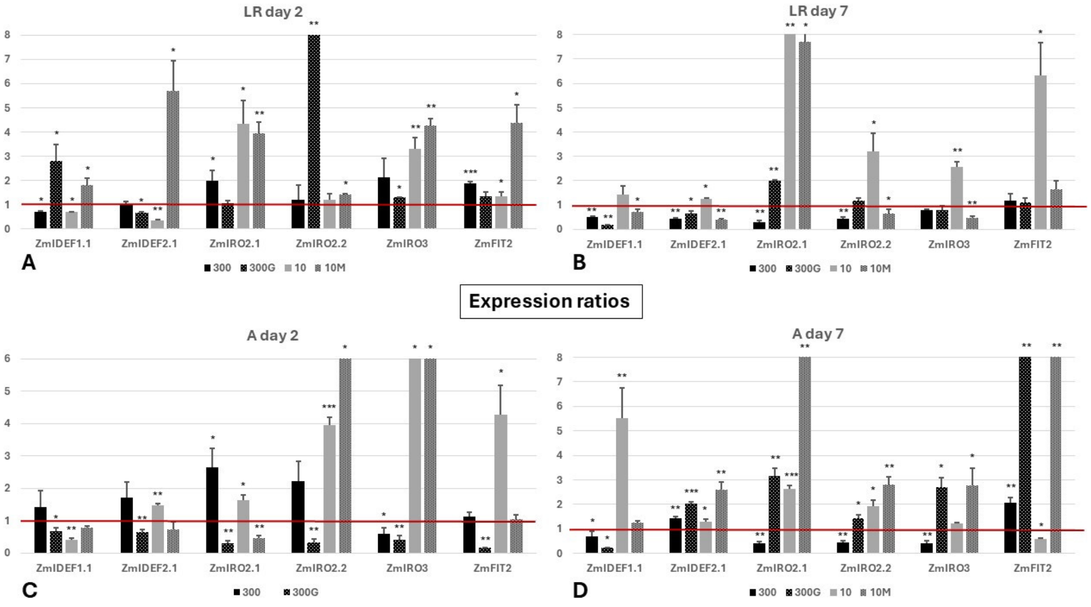

Figure S4. Detailed depiction of gene expression ratios of Figure 11 at low ratio values. Gene expression ratios of Fe homeostasis-regulating transcription factors in the LR (A,B) and A (C,D) in the CR1 roots of maize seedlings on day 2 and day 7. Genes included the ABI member TF *ZmIDEF1.1*, the NAC member TF *ZmIDEF2.1*, and the bHLH TFs *ZmIRO2.1*, *ZmIRO2.2*, *ZmIRO3*, and *ZmFIT2*. 300: maize seedlings grown in iron-sufficient conditions (300 µM Fe). 300G: maize seedlings grown under iron sufficiency (300 µM Fe) in the presence of exogenous gibberellic acid (G). 10: maize seedlings grown in iron-insufficient conditions (10 µM Fe). 10M: maize seedlings grown under iron insufficiency (10 µM Fe) but with the addition of the gibberellin biosynthesis inhibitor mepiquat chloride (M) in the growth medium. LR: lateral root sector, A: root apex, CR1: first row of crown roots. Mean ± SE. Asterisks indicate statistically significant overexpression or downregulation. $p < 0.05$ (*), $p < 0.01$ (**), $p < 0.001$ (***).

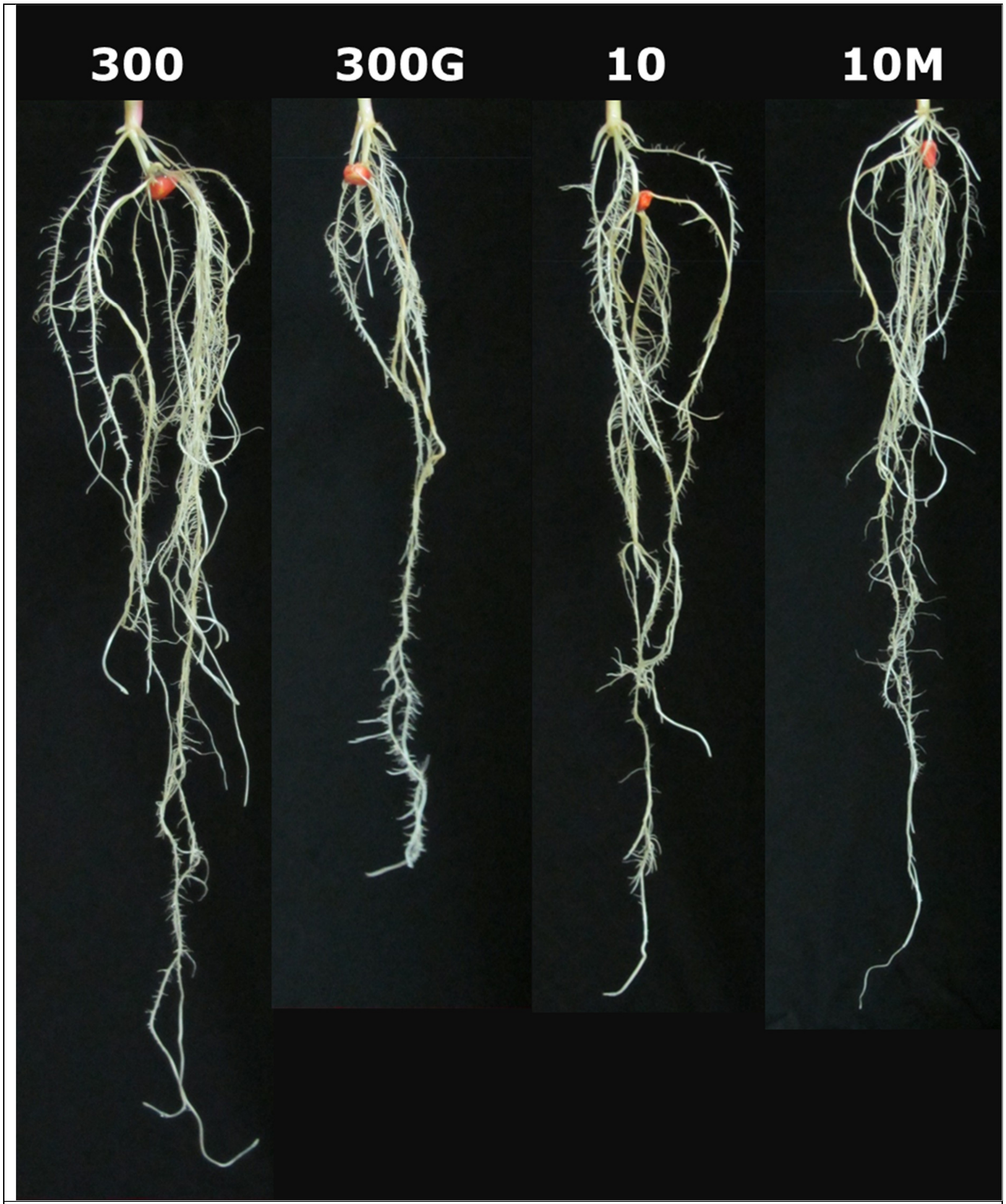

Figure S5. Comparison between whole root systems of 300, 300G, 10, and 10M plants on day 7. 300: maize seedlings grown in iron sufficient conditions (300μM Fe). 300G: maize seedlings grown under iron sufficiency (300μM Fe) in the presence of exogenous gibberellic acid (G). 10: maize seedlings grown in iron insufficient conditions (10μM Fe). 10M: maize seedlings grown under iron insufficiency (10μM Fe) but with the addition of the gibberellin biosynthesis inhibitor Mepiquat Chloride (M) in the growth medium.

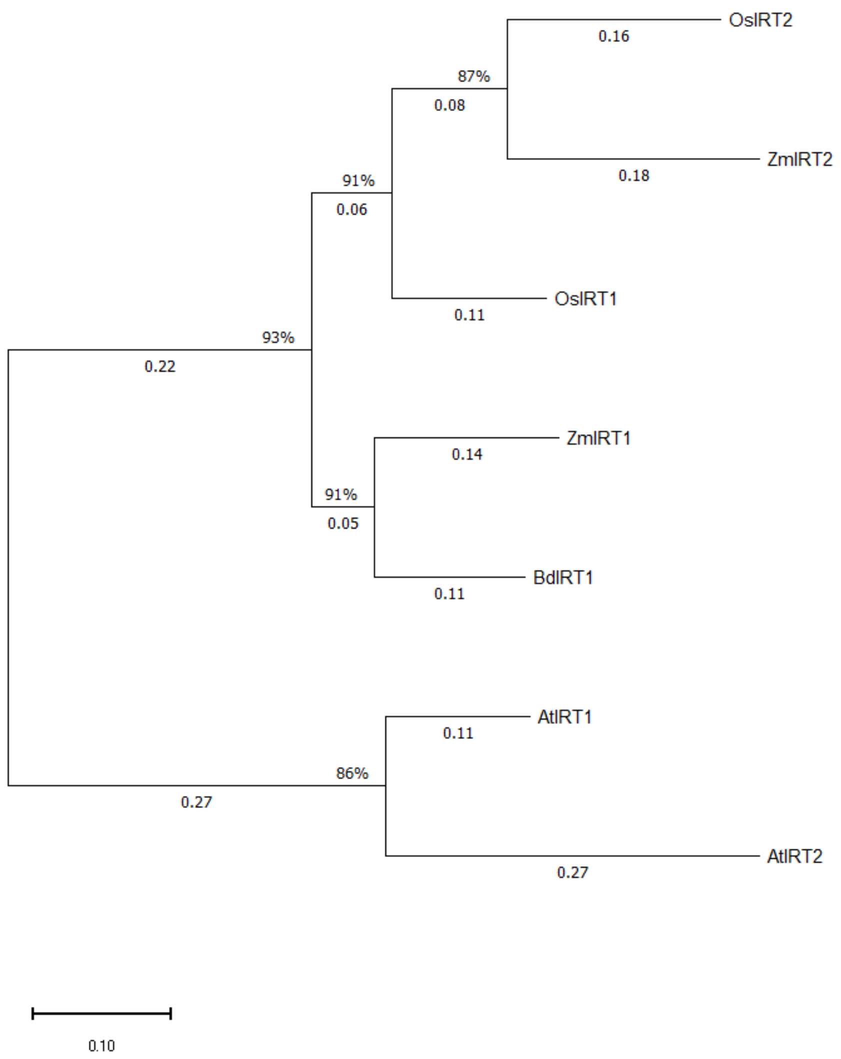

Figure S6. Evolutionary analysis by the maximum likelihood method among the Iron-Regulated Transporter (IRT) protein sequences of *Arabidopsis thaliana, Oryza sativa, Brachypodium distachyon,* and *Zea mays*. The evolutionary history was deduced using the maximum likelihood method and the Jones-Taylor-Thornton (JTT) matrix-based model (Jones et al., 1992). Evolutionary analyses were carried out by MEGA X (Kumar et al., 2018). List of the genes included in the phylogenetic tree are listed in Tables S1 and S2.

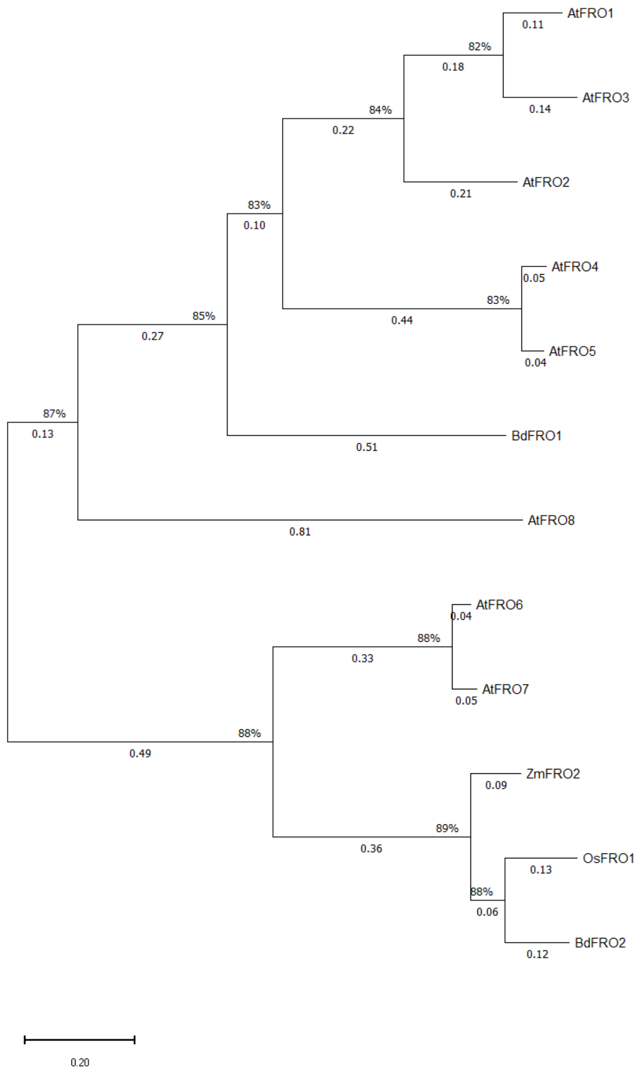

Figure S7. Evolutionary analysis by the maximum likelihood method among the Fe(III)-chelate Reductase (FRO) protein sequences from *Arabidopsis thaliana*, as well as ZmFRO2 from *Zea mays*, OsFRO1 from *Oryza sativa*, and BdFRO1 and BdFRO2 from *Brachypodium distachyon*. List of the genes included in the phylogenetic tree are listed in Tables S1 and S2.

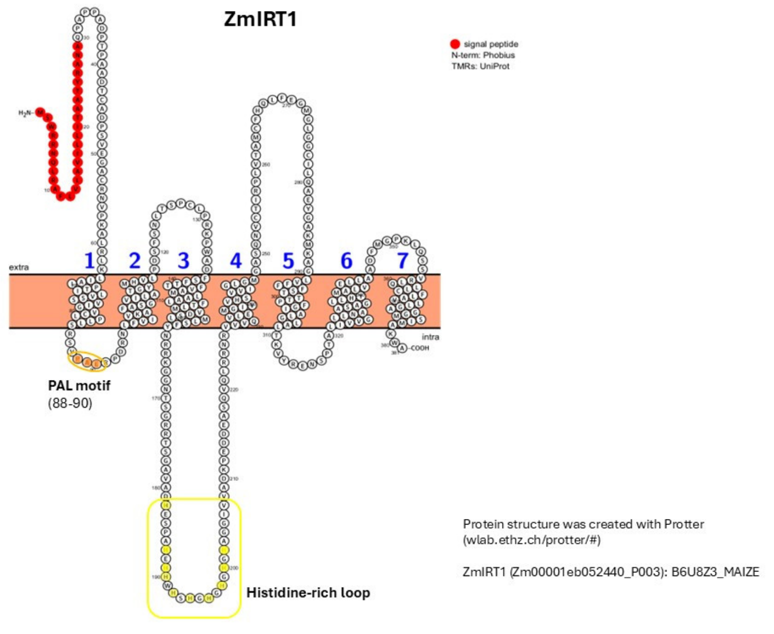

Figure S8. Protein structure of ZmIRT1 created with Protter.

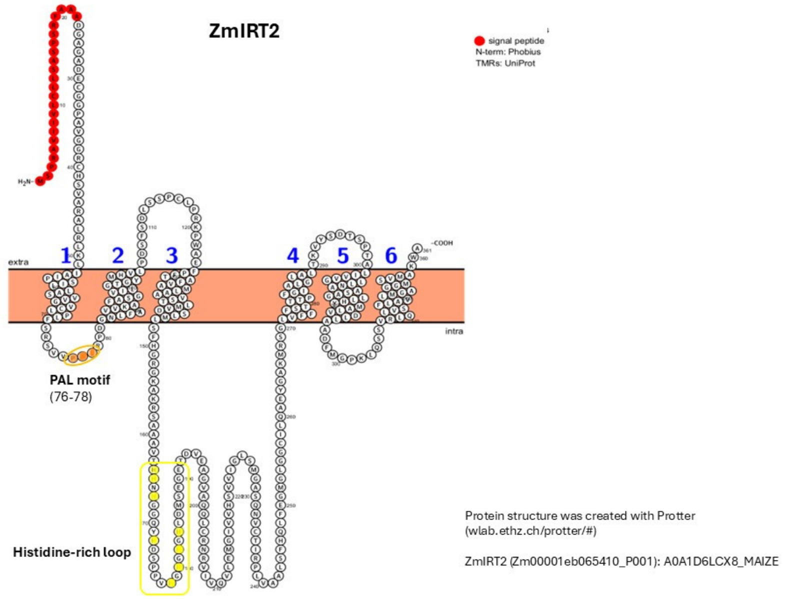

Figure S9. Protein structure of ZmIRT2 created with Protter.

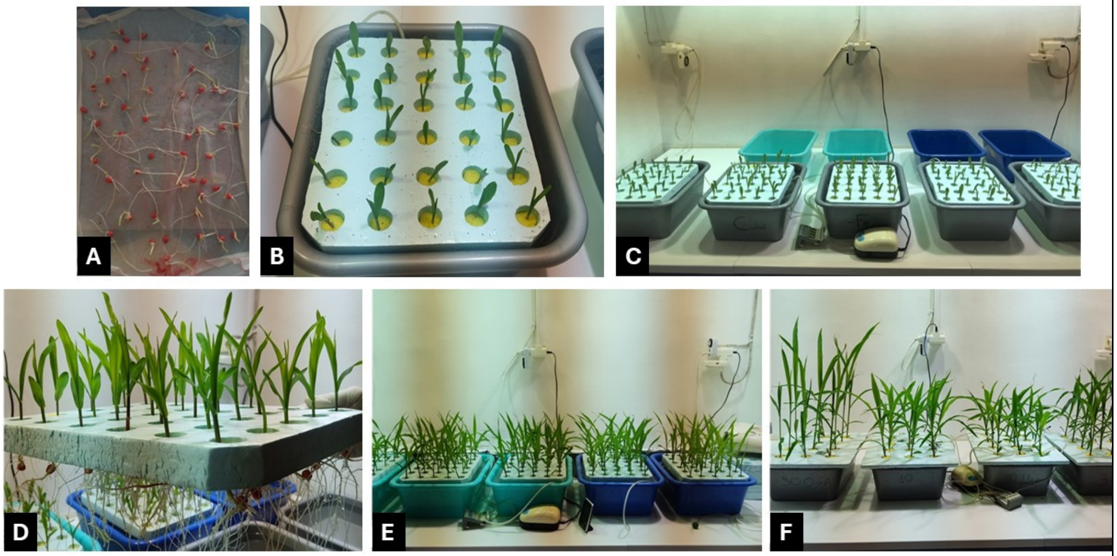

Figure S10. Plant growth timeline. Maize seeds were placed on wet filter paper for germination (A) and after four days the seedlings were positioned in containers filled with distilled water under sufficient aeration, for a total of three days (B). Subsequently, the plants were transferred in a second hydroponic batch culture containing a complete nutrient solution with 100μM Fe (C,D). A week later, the seedlings were divided into four treatment groups (300, 300G, 10, 10M), marking treatment initiation (day 0) (E). The plants were kept under these conditions for a total of seven days (day 7) (F, from left to right: 300G, 10, 10M, 300). All nutrient solutions were constantly aerated and were replaced every three days. Growth chamber conditions were 24/18 °C, relative humidity 40 %, 250 μmol photon m^{-2} s^{-1} and a 16-h photoperiod. 300: maize seedlings grown in iron sufficient conditions (300μM Fe). 300G: maize seedlings grown under iron sufficiency (300μM Fe) in the presence of exogenous gibberellic acid (G). 10: maize seedlings grown in iron insufficient conditions (10μM Fe). 10M: maize seedlings grown under iron insufficiency (10μM Fe) but with the addition of the gibberellin biosynthesis inhibitor Mepiquat Chloride (M) in the growth medium.

Dataset S1

Bioinformatic analysis was performed using the data from the Maize Genetics and Genomic Database (MGDB): https://www.maizegdb.org/

The study of Benke et al. [20] revealed only one quantitative trait locus associated with reduced root length (RL) under Fe deficiency. This QTL is located in chromosome 3, between the flanking markers "*jpsb79-umc60*".

The genes found in the locus "*jpsb79-umc60*" are listed in Table S4. Among these 4 genes, *Zm00001eb148290* codes for GRAS23, a GRAS family transcription factor. GRAS family TFs are major players in gibberellin (GA) signaling (response to GAs).

Table S4. List of the genes in the QTL located in chromosome 1, between the flanking markers "*chrom7-glb1*" (V4 annotation of MGDB).

MGDB Gene ID	MGDB Gene/Protein Information
Zm00001eb148290	gras23 - GRAS-transcription factor 23
Zm00001eb148300	Ribonuclease II/R domain-containing protein
Zm00001eb148310	Elicitor-responsive protein 1
Zm00001eb148320	myb15 - MYB-transcription factor 15

Dataset S2

***In silico* 1000bp core promoter analysis**

Tool: PlantCARE

(http://bioinformatics.psb.ugent.be/webtools/plantcare/html/)

Key:

Gibberellin response: GARE-motif, TATC-box

Abscisic acid response: ABRE, ABRE3a, ABRE4, AT~ABRE

Anaerobic response: GC-motif

1. **ZmNAS1**

```
+ GAGAATGATA GTCCAATGAA ATTTGTTAGG GTGAGTGGAG TTCGTTCGTT GCATATTCAA ACATACATTC
- CTCTTACTAT CAGGTTACTT TAAACAATCC CACTCACCTC AAGCAAGCAA CGTATAAGTT TGTATGTAAG

+ TAGTTACATA ATAAAAAATT GACGTTTTGT TCTCTTATAT GTCTTATATG TAGGATACAA AGATTCTTCG
- ATCAATGTAT TATTTTTTAA CTGCAAAACA AGAGAATATA CAGAATATAC ATCCTATGTT TCTAAGAAGC

+ GATAAACTTT ATTATCGAAC TGTTAAGTTA CGAGGAAAAT TCGTGCAGAC ATGCCATCCC AGCAAATATA
- CTATTTGAAA TAATAGCTTG ACAATTCAAT GCTCCTTTTA AGCACGTCTG TACGGTAGGG TCGTTTATAT

+ CAACAGAGAG TTAACAATAT TGTTAAAAAA TATTAGATTA GTGTACAAGG TGTCAACATA TTTACTTATG
- GTTGTCTCTC AATTGTTATA ACAATTTTTT ATAATCTAAT CACATGTTCC ACAGTTGTAT AAATGAATAC

+ ATTAAGCATT ATATATTTAC TCTTTAGAAA TTATTTATGT GATTCTATTT CAATCTATTT TGGTAGACAT
- TAATTCGTAA TATATAAATG AGAAATCTTT AATAAATACA CTAAGATAAA GTTAGATAAA ACCATCTGTA

+ ATTTATATCG TTCTGTATCT ATGTTTAATA ATCACATATT ATACCGTCAC TATTGTACTC ACGCCGCACG
- TAAATATAGC AAGACATAGA TACAAATTAT TAGTGTATAA TATGGCAGTG ATAACATGAG TGCGGCGTGC

+ TCGTCAGGGC CCTAAATCCA TTGGAGGGTC GCAATTTGTG TTCCGTGGCA TCGCATGGTA ACGTTCCTAG
- AGCAGTCCCG GGATTTAGGT AACCTCCCAG CGTTAAACAC AAGGCACCGT AGCGTACCAT TGCAAGGATC

+ TTATATATTG ATAGCTGCAA GACGTACATC ATGTCAACTT TAGAGAGAGA AAGACAAGAG ATTGGTTTGC
- AATATATAAC TATCGACGTT CTGCATGTAG TACAGTTGAA ATCTCTCTCT TTCTGTTCTC TAACCAAACG

+ TAGCTCTTCC CTTTATTGTG ATGATTGAGG CCGGGAGCAG TTTTCTTAAT TAAGATTTTT GAAAGAACTT
- ATCGAGAAGG GAAATAACAC TACTAACTCC GGCCCTCGTC AAAAGAATTA ATTCTAAAAA CTTTCTTGAA

+ GTATTTTGTT TTTAATCTTT TTATCAGTGC TCGACCAATG TCATGATGTG GACGTGGCAA ACCCACTTCG
- CATAAAACAA AAATTAGAAA AATAGTCACG AGCTGGTTAC AGTACTACAC CTGCACCGTT TGGGTGAAGC

+ GGAGGGGACA AAAAAAACAG GGCTGCCACT TTCAGGGCGT GACGTGGACG TGTTGAAGAT CCGGAACATT
- CCTCCCCTGT TTTTTTTGTC CCGACGGTGA AAGTCCCGCA CTGCACCTGC ACAACTTCTA GGCCTTGTAA

+ CTTCGTGAAA CGTACACCGT CAAAATAGCG AGGCATGAAA CTGGCCTTGG CCATGGACGC GTGAAGCGCG
- GAAGCACTTT GCATGTGGCA GTTTTATCGC TCCGTACTTT GACCGGAACC GGTACCTGCG CACTTCGCGC

+ CCATGCGTTG GATATGTGGT CAATAAGTAT ATACAATACA ATGTTTAACA GAGCTGGTAG TACTGCTTCG
- GGTACGCAAC CTATACACCA GTTATTCATA TATGTTATGT TACAAATTGT CTCGACCATC ATGACGAAGC

+ GCACATTTTT GTCCACGCTT CATGAGAGAC AAAAACACCT GCACTTAAAT TCACATGCTG CACTGAAGGC
- CGTGTAAAAA CAGGTGCGAA GTACTCTCTG TTTTTGTGGA CGTGAATTTA AGTGTACGAC GTGACTTCCG

+ CCGATCACTG AGGAGCGAA
- GGCTAGTGAC TCCTCGCTT
```

GARE-motif

ABRE

2. ZmDMAS1

```
+ GAGTGTTGGA ATTTATACAT ATCCCCAGAC TTTTGGTACT GTTGTATTTG CATTACTTCC AGCGATGCTT
- CTCACAACCT TAAATATGTA TAGGGGTCTG AAAACCATGA CAACATAAAC GTAATGAAGG TCGCTACGAA

+ TCTTAGGATT GTGGACCTAT TGGTTCTTTA AACGTGGTGG CTGATTCGCT TCCTTCGACT GAGCTCGCAT
- AGAATCCTAA CACCTGGATA ACCAAGAAAT TTGCACCACC GACTAAGCGA AGGAAGCTGA CTCGAGCGTA

+ CTTCGGTACG TTGTTTTCTT CTGACCTCGA TGGTTCCCAA TTTGGTTTCA GCTTACACGC GTCGAAGACC
- GAAGCCATGC AACAAAAGAA GACTGGAGCT ACCAAGGGTT AAACCAAAGT CGAATGTGCG CAGCTTCTGG

+ CGGGTAACTG TCAGGAAAAC GAGTAGGATG AAAGCGAAGC TCGCGGTGCT TGGGCGCGAG CCGCCCGGCG
- GCCCATTGAC AGTCCTTTTG CTCATCCTAC TTTCGCTTCG AGCGCCACGA ACCCGCGCTC GGCGGGCCGC

+ TTACAAGTTG CAATGCAACT CTGGTGGAGG CTATCGCAAG TCACTACCCA TATGAGGCCA CGTTCGTTTG
- AATGTTCAAC GTTACGTTGA GACCACCTCC GATAGCGTTC AGTGATGGGT ATACTCCGGT GCAAGCAAAC

+ ATCCCAAAAC CATAGGGATT AAGGGGGATT GGAAAGGATT AGAGAGGATT TTGACTCGTA GAGAATTTGA
- TAGGGTTTTG GTATCCCTAA TTCCCCCTAA CCTTTCCTAA TCTCTCCTAA AACTGAGCAT CTCTTAAACT

+ TCCCCCTTCA ATCCCTATGG ATCAGCACAA AACGAACGGG GCCTGACGGA GGACGGGAGG AGGCTTGTTT
- AGGGGGAAGT TAGGGATACC TAGTCGTGTT TTGCTTGCCC CGGACTGCCT CCTGCCCTCC TCCGAACAAA

+ GTCCTATAAC GGCGTCGCGG AGCCTCAGCC AGCATATGTT CTTGTTGGAT GCCGGAGTAG CGGAGTGGGC
- CAGGATATTG CCGCAGCGCC TCGGAGTCGG TCGTATACAA GAACAACCTA CGGCCTCATC GCCTCACCCG

+ GAATGGACTG CACGCTCTCA TGCCATGCGG CACGCCTACG ACTACGAGAT GCACGGCGAC GCTGACGCAG
- CTTACCTGAC GTGCGAGAGT ACGGTACGCC GTGCGGATGC TGATGCTCTA CGTGCCGCTG CGACTGCGTC

+ CGCGGGGCCC GCGGGGACGG ACGGCATGAA CCCGTCCAGT GTCTCGGGTC TCAGAGCTAC AAACAAAGCC
- GCGCCCCGGG CGCCCCTGCC TGCCGTACTT GGGCAGGTCA CAGAGCCCAG AGTCTCGATG TTTGTTTCGG

+ GCATGGTGGC ATGATCTGTG CCTGTCCCGG TGGTGAGTAG TGACGATCGC TGCCGCTTTT CCCCCATCGG
- CGTACCACCG TACTAGACAC GGACAGGGCC ACCACTCATC ACTGCTAGCG ACGGCGAAAA GGGGGTAGCC

+ CCGCCGCAGC ATGACATCAC ATATCAGTGG ACTGGAGCAG GCAACAGAAG ATCTCGAACT CGAAGCGTGA
- GGCGGCGTCG TACTGTAGTG TATAGTCACC TGACCTCGTC CGTTGTCTTC TAGAGCTTGA GCTTCGCACT

+ AAACCACTGC TAGAGCATTT CATGTTACAG CCCGCGAAAA CGTAAAAAAA ATCGAAGGAA TCCGAGGAGA
- TTTGGTGACG ATCTCGTAAA GTACAATGTC GGGCGCTTTT GCATTTTTTT TAGCTTCCTT AGGCTCCTCT

+ AAATCAGGCA AGTCATGGCT ACCGGACCCC GAACGCTCCT CAGCTCCTTG GACGTCCAAA AAACAAACGC
- TTTAGTCCGT TCAGTACCGA TGGCCTGGGG CTTGCGAGGA GTCGAGGAAC CTGCAGGTTT TTTGTTTGCG

+ AAACTTTTGT TGTTTGTAG
- TTTGAAAACA ACAAACATC
```

ABRE

GARE-motif

3. ZmTOM1

```
+ TGGATGGAGT AAACAACAAC ATCAGTTAGT GAAATCAAAA AAATATTATG CAGAGAGCAG AGACAATTAA
- ACCTACCTCA TTTGTTGTTG TAGTCAATCA CTTTAGTTTT TTTATAATAC GTCTCTCGTC TCTGTTAATT

+ TAAAAATCTT GAGATCTTTT TGATGGATAG TTTACGTATA TATTGTTGTG AGCCGTCGCA AAACGTACTG
- ATTTTTAGAA CTCTAGAAAA ACTACCTATC AAATGCATAT ATAACAACAC TCGGCAGCGT TTTGCATGAC

+ ACATTATATA TTGTCCAATC GTCGAACCGA CGACTGAAGG TTAATTGATA GTGATACTGG TGGCGCCCAG
- TGTAATATAT AACAGGTTAG CAGCTTGGCT GCTGACTTCC AATTAACTAT CACTATGACC ACCGCGGGTC

+ GCCGGCCGGC CGTAGACTAG CATGCATGCA TGCAGAGCTT GGTTAGTTTC CTGTGGTGTG ACTTGGTCAT
- CGGCCGGCCG GCATCTGATC GTACGTACGT ACGTCTCGAA CCAATCAAAG GACACCACAC TGAACCAGTA

+ CAATTAAGCG AGAGGCAGCT ATGCATATGT CATGTGTACA TGCATGTGTG GGTGGAGCGA TTGGCACATT
- GTTAATTCGC TCTCCGTCGA TACGTATACA GTACACATGT ACGTACACAC CCACCTCGCT AACCGTGTAA

+ TCACGTTGTC TCCCTTGCTT CTTGCACTGT GCGTGCTCAT TGTGCCAGGG TTTGACTCCG CATATATAAA
- AGTGCAACAG AGGGAACGAA GAACGTGACA CGCACGAGTA ACACGGTCCC AAACTGAGGC GTATATATTT

+ TAGCTTCCTC TCCGTCAGCG CTGACACAGT ACGTCTTACT AACCATATTA TGATACTCAA ATTACCTGCC
- ATCGAAGGAG AGGCAGTCGC GACTGTGTCA TGCAGAATGA TTGGTATAAT ACTATGAGTT TAATGGACGG

+ CACCATCCCC TGCAAAACTA GCCACACATA TATGTAAGTA TGTGTATTTG TTTGCTGCAG CGCTTCTCCT
- GTGGTAGGGG ACGTTTTGAT CGGTGTGTAT ATACATTCAT ACACATAAAC AAACGACGTC GCGAAGAGGA

+ TCAAGTACGT ACGTGTCGCC TGTGCCAATG GATAATTTGA TTTGCCTCGG CATAAATATA TACAAACTGT
- AGTTCATGCA TGCACAGCGG ACACGGTTAC CTATTAAACT AAACGGAGCC GTATTTATAT ATGTTTGACA

+ GTGGCCTAGC TAGCTTACCA ACCGTCTTTT GTCCTTGTAT ACTATGACAA CAAGTTGGTT ATACTCCGCG
- CACCGGATCG ATCGAATGGT TGGCAGAAAA CAGGAACATA TGATACTGTT GTTCAACCAA TATGAGGCGC

+ CGCCTTGCAT CAGGAAATCG ACGTAGTGCA AAAGCGGGCG GGAGGGCGGC GGCATGTGCA TGCATGCCGC
- GCGGAACGTA GTCCTTTAGC TGCATCACGT TTTCGCCCGC CCTCCCGCCG CCGTACACGT ACGTACGGCG

+ CCATCGGCGC GTACGCATAT GCCACGCACG CTACACAGAG ACGTGCGTAC GTACGTAGAG CAGTCTCGCT
- GGTAGCCGCG CATGCGTATA CGGTGCGTGC GATGTGTCTC TGCACGCATG CATGCATCTC GTCAGAGCGA

+ CGTCCAGCTC GATCTACACA ACATCTGGCT GACTAGCTAC ATGCAGTGTG CATGTCGTCA TTATTACTCA
- GCAGGTCGAG CTAGATGTGT TGTAGACCGA CTGATCGATG TACGTCACAC GTACAGCAGT AATAATGAGT

+ CCTCGGTAGT AGCATTACAA TGTATGTATG TATATATAAC CCATGCAGGC AGCTACCACC CGGCCAAGAG
- GGAGCCATCA TCGTAATGTT ACATACATAC ATATATATTG GGTACGTCCG TCGATGGTGG GCCGGTTCTC

+ CAGTATTCCA GGCCCAGGG
- GTCATAAGGT CCGGGTCCC
```

ABRE

RY-element

4. ZmYS1

```
+ CTTGCGTATT TGCTGTTGGC GGCTGCCGAC CCGCTGCACG CCTGCACCTC CCTGGCTCCC TCCCCGGCTC
- GAACGCATAA ACGACAACCG CCGACGGCTG GGCGACGTGC GGACGTGGAG GGACCGAGGG AGGGGCCGAG

+ CCGCGCCGGT GCCTCTGCCT TACTGCGTGG GCCGCCTCCG TCGACCTCGT GCGCTCGCAG CTCGGCCTCG
- GGCGCGGCCA CGGAGACGGA ATGACGCACC CGGCGGAGGC AGCTGGAGCA CGCGAGCGTC GAGCCGGAGC

+ TGCCATCGTC ATCCGCGTGC GCGCCACCGC GTGGACCGCC TCCGTCGGCC TCGTTGTAAT ACTTGGCGGT
- ACGGTAGCAG TAGGCGCACG CGCGGTGGCG CACCTGGCGG AGGCAGCCGG AGCAACATTA TGAACCGCCA

+ GTACACGTGG GACTAAAAGG AAAACCAGCA ATAGTGACAC ATAGGGGTCA GGGGAACTAA AAGCACAACC
- CATGTGCACC CTGATTTTCC TTTTGGTCGT TATCACTGTG TATCCCCAGT CCCCTTGATT TTCGTGTTGG

+ CAATGCCGAC GAAGACAACG CATAATTTTC AGTGGCACCG TTAAGGAAAA TTTATATATT TTAACGGCAT
- GTTACGGCTG CTTCTGTTGC GTATTAAAAG TCACCGTGGC AATTCCTTTT AAATATATAA AATTGCCGTA

+ TCTAAAAGAT CTCTTAAATT TATATGTCTA AAACAAATTA GAATGAAATT TATATGTCTA GACAAATTAG
- AGATTTTCTA GAGAATTTAA ATATACAGAT TTTGTTTAAT CTTACTTTAA ATATACAGAT CTGTTTAATC

+ AATGACTTAT AATCTAGAAC AGGTACTACA AAAGTCGGTC CAGTGACTCC AGTAGGCTCT TCTGGACTAA
- TTACTGAATA TTAGATCTTG TCCATGATGT TTTCAGCCAG GTCACTGAGG TCATCCGAGA AGACCTGATT

+ CGGTAATAGG ACATGTTTTT TTATAGAATA TACACAAGCA TATTTGTTAA CGAGCACTTA GCAGATATGA
- GCCATTATCC TGTACAAAAA AATATCTTAT ATGTGTTCGT ATAAACAATT GCTCGTGAAT CGTCTATACT

+ GTACTGGACT AACGATAATA TGTCACGATT CCCCGATAAA CTTCGTTCCA AAGAAGTCAT AACCGTTTGG
- CATGACCTGA TTGCTATTAT ACAGTGCTAA GGGGCTATTT GAAGCAAGGT TTCTTCAGTA TTGGCAAACC

+ TTCACTAAAT GTAACGTAAA TGATAATGAT AACGGTTCAC ACTCGAATAC CGGCGGTAAC AAATTTGAAT
- AAGTGATTTA CATTGCATTT ACTATTACTA TTGCCAAGTG TGAGCTTATG GCCGCCATTG TTTAAACTTA

+ GAGACGATAT CCATTTGTAG TATGATATCG ATTACGATTG GACTTAAACA AACATGATTT AACGTTATCG
- CTCTGCTATA GGTAAACATC ATACTATAGC TAATGCTAAC CTGAATTTGT TTGTACTAAA TTGCAATAGC

+ GTTACCGATT ACGTTACCAA TACGTGAACC AAACGGCACC TAACTTCCAT CAATTTGTCC GTGAGATGAA
- CAATGGCTAA TGCAATGGTT ATGCACTTGG TTTGCCGTGG ATTGAAGGTA GTTAAACAGG CACTCTACTT

+ CTATATACAG GTATACAGAC ATGAAGGCAA GAAAAATGCT GCACCGATCA AGTCGCTTTC CTTCACCATT
- GATATATGTC CATATGTCTG TACTTCCGTT CTTTTTACGA CGTGGCTAGT TCAGCGAAAG GAAGTGGTAA

+ CTCCCCCCTA ACAACTCTCT CGACAGCTAA TAATGCGACG ACTTGGAAAT GTCTTAGTAT TTTTCTGCCA
- GAGGGGGGAT TGTTGAGAGA GCTGTCGATT ATTACGCTGC TGAACCTTTA CAGAATCATA AAAAGACGGT

+ CGGAAATAGT TAAGGGTTT
- GCCTTTATCA ATTCCCAAA
```

ABRE

ABRE3a

5. ZmNAS3

+ ATCTCTGCTG TAGCGTACAC TAGCGTGTAT GAACTCTAGT GCAGGACGTA TATGTTCCTT GGGAATAGCG
- TAGAGACGAC ATCGCATGTG ATCGCACATA CTTGAGATCA CGTCCTGCAT ATACAAGGAA CCCTTATCGC

+ TAAAGTAGAG AAAGGTAGAT AGATAGATAG ATATTCTTGG GGATCGGAAT AAGTATATTT GAAGTAAAGG
- ATTTCATCTC TTTCCATCTA TCTATCTATC TATAAGAACC CCTAGCCTTA TTCATATAAA CTTCATTTCC

+ AAATATGAGG CAACGACGAC ATGCATGGAA CTTGAATTAT TGTTATATGG GGCATCGTAG CTATGCTATA
- TTTATACTCC GTTGCTGCTG TACGTACCTT GAACTTAATA ACAATATACC CCGTAGCATC GATACGATAT

+ TTATCGTGAC AGAAATGCAC GGCCGTTCCT GACTTTTGAG GGGCCCAGGA CAAAATTATA AACAGAGGCC
- AATAGCACTG TCTTTACGTG CCGGCAAGGA CTGAAAACTC CCCGGGTCCT GTTTTAATAT TTGTCTCCGG

+ CCAACACCAT AAAAACTATT TTTTTACTAT TATATATATC AACTAATTAT ACTTGATGTA CCACAGAAAG
- GGTTGTGGTA TTTTTGATAA AAAAATGATA ATATATATAG TTGATTAATA TGAACTACAT GGTGTCTTTC

+ ACTTGAGCAG TAAAAAATAG AAGTAAGATT ATTACCTCAA ATATATTGTA GAGATCAACT TAAAAATGTC
- TGAACTCGTC ATTTTTTATC TTCATTCTAA TAATGGAGTT TATATAACAT CTCTAGTTGA ATTTTTACAG

+ TTCTAACGTT TCTAAATGCA AAATCATCAA TGATGGCTTC AATATTGACA TCATCTAATA ATTTCTTCTC
- AAGATTGCAA AGATTTACGT TTTAGTAGTT ACTACCGAAG TTATAACTGT AGTAGATTAT TAAAGAAGAG

+ AATACATAAA GTTGCTAACC CGTCTAACCT TTCTTGAGAA ATTATAGACC TTAAATAATT CTTCAATAAC
- TTATGTATTT CAACGATTGG GCAGATTGGA AAGAACTCTT TAATATCTGG AATTTATTAA GAAGTTATTG

+ TTAAGCTTTG AGAAGCTCCT TTAAGCTCAT GCCACAGTTA TTGATATAGT AAATAATATC TGATAAGCAA
- AATTCGAAAC TCTTCGAGGA AATTCGAGTA CGGTGTCAAT AACTATATCA TTTATTATAG ACTATTCGTT

+ CAAATATATT GGGATATCAA TCCATTCTCC TCAGAAACTC AAAAATATAT TAGTATAGTA AATAATATCT
- GTTTATATAA CCCTATAGTT AGGTAAGAGG AGTCTTTGAG TTTTTATATA ATCATATCAT TTATTATAGA

+ GTTTCTATCC TATATTCCTA TTAGACTTTG TGTGCGGTGG GGCCCCTGGG AGATGGGGGC CCAGATCGGC
- CAAAGATAGG ATATAAGGAT AATCTGAAAC ACACGCCACC CCGGGGACCC TCTACCCCCG GGTCTAGCCG

+ CGCCCCTCCC GCCCTGCCTC AGGCACGGCC CTGCAGAAAT GAAATGGAAC TTCACTCGCC AGTGCAAGTT
- GCGGGGAGGG CGGGACGGAG TCCGTGCCGG GACGTCTTTA CTTTACCTTG AAGTGAGCGG TCACGTTCAA

+ GTACGCGTGC TGTGGCGACT GGCGACGACG ACGACTGCGA CAAGGACGCC GTTGCGTGTG TGGAGAAAAC
- CATGCGCACG ACACCGCTGA CCGCTGCTGC TGCTGACGCT GTTCCTGCGG CAACGCACAC ACCTCTTTTG

+ AACGCAGAGG GCTTGTGGCG GGCACGCGTC TACAAGACAG GGACGCCATT ATTCCCCCAC CACCATATAT
- TTGCGTCTCC CGAACACCGC CCGTGCGCAG ATGTTCTGTC CCTGCGGTAA TAAGGGGGTG GTGGTATATA

+ ATATAGTGTA CTATGCCTC
- TATATCACAT GATACGGAG

RY-element

TATC-box

6. ZmIRT1

```
+ CCTTCGTGCT GTTCTTCCTC TCCAACCTGC TGCCCCGCCC ACGGTCGCAG CCGCGAGCCG ACCGATGCTC
- GGAAGCACGA CAAGAAGGAG AGGTTGGACG ACGGGGCGGG TGCCAGCGTC GGCGCTCGGC TGGCTACGAG

+ GTCGACGAGG GTACGGGGCG AGTCGGTCAT GCTCTTGGTC TTTCTCCGTG CGTGCTATCG AGGAGGACAT
- CAGCTGCTCC CATGCCCCGC TCAGCCAGTA CGAGAACCAG AAAGAGGCAC GCACGATAGC TCCTCCTGTA

+ GGTGGCACCT GCCACGCTTA GGTTATCTTG GCGATTAGGA GTCCAGGCCT GAGATATTGG ACAATCAAGA
- CCACCGTGGA CGGTGCGAAT CCAATAGAAC CGCTAATCCT CAGGTCCGGA CTCTATAACC TGTTAGTTCT

+ CCCGTAGCGT GGCAGCAGTC GGCATATGCT ACGGAGTTGG TGGTGGTGTT GTGTTGCTTC GGCGGGTCCA
- GGGCATCGCA CCGTCGTCAG CCGTATACGA TGCCTCAACC ACCACCACAA CACAACGAAG CCGCCCAGGT

+ GGGCTGGGAA GACATTCGCA TTTTCTCGCC CTTCTCGGAT TGGCGCCTGG TGCGGGTGCC TCTCGGTTTG
- CCCGACCCTT CTGTAAGCGT AAAAGAGCGG GAAGAGCCTA ACCGCGGACC ACGCCCACGG AGAGCCAAAC

+ GAGCTGCTCC GGCTGGGCTT CTTTCTCCAC TTGCATGCTC TCTCCCTATA TATCTAGCGG TATACTACCT
- CTCGACGAGG CCGACCCGAA GAAAGAGGTG AACGTACGAG AGAGGGATAT ATAGATCGCC ATATGATGGA

+ ATGTCATCTC CAGATGACCA GGTCTTCGTC GTGTCCTTCT CCGGCAACGA ACAGGTATGT CCGCATCTTT
- TACAGTAGAG GTCTACTGGT CCAGAAGCAG CACAGGAAGA GGCCGTTGCT TGTCCATACA GGCGTAGAAA

+ CCTTCCCCTC CACTCCTGCT CTACGAAACC TTGGAAGTGG GAGGGCAAGG GATTGGGGTC TCTGATTTGC
- GGAAGGGGAG GTGAGGACGA GATGCTTTGG AACCTTCACC CTCCCGTTCC CTAACCCCAG AGACTAAACG

+ TGTTTATGTT AACCGTGCGT TCAAAAAAAA TTACGTGAAA AGTAGAGACA ATCAATAAAA AAACTTGAGA
- ACAAATACAA TTGGCACGCA AGTTTTTTTT AATGCACTTT TCATCTCTGT TAGTTATTTT TTTGAACTCT

+ TCTTTTTGTG GATAATTTAT GTGGTTATTG TTGTGAGCCG TCGCAACGCA CAGGCTAGTA TGGTATTAAA
- AGAAAAACAC CTATTAAATA CACCAATAAC AACACTCGGC AGCGTTGCGT GTCCGATCAT ACCATAATTT

+ GACACAGATC GGCCTTCACA TGAAAAATAG TGTGGAAATA TAAGTGAGTT CTTCGTCTCT TATAAGTGAA
- CTGTGTCTAG CCGGAAGTGT ACTTTTTATC ACACCTTTAT ATTCACTCAA GAAGCAGAGA ATATTCACTT

+ TACCAGTTAA CGCTACTGAA TAATGTTAGG ATGTGTCCTC TACACTAGCC GATTAACAGT AACCGGGGTA
- ATGGTCAATT GCGATGACTT ATTACAATCC TACACAGGAG ATGTGATCGG CTAATTGTCA TTGGCCCCAT

+ GGGTTAATCC TAACCGTCTA ACCGCGCCCC TGATCGGGGG GCTATCCTAA CCGTTGGTTG CAGCCCCCGG
- CCCAATTAGG ATTGGCAGAT TGGCGCGGGG ACTAGCCCCC CGATAGGATT GGCAACCAAC GTCGGGGGCC

+ TCACACGACG CCATAACGTG TGCTGGAAGT GAAAGACCAT CCAAGTTGCT CCAGGCCCTA TATATATATG
- AGTGTGCTGC GGTATTGCAC ACGACCTTCA CTTTCTGGTA GGTTCAACGA GGTCCGGGAT ATATATATAC

+ TGCAACCCCT ACAACAGCC
- ACGTTGGGGA TGTTGTCGG
```

ABRE

ABRE3a

ABRE4

GC-motif

7. ZmIRT2

```
+ CATTAAACAC TCGTTCATTC CTTAGACGTT GATCAATGCA CATGGACTCC TACACTTGTG ATAATTGCAT
- GTAATTTGTG AGCAAGTAAG GAATCTGCAA CTAGTTACGT GTACCTGAGG ATGTGAACAC TATTAACGTA

+ ACTGCAGCAC GAAGAGGCGA TACTCCATCT ATTCCTTAGA TGCAATTTTG CTAGAAGATG TTGGGTTATA
- TGACGTCGTG CTTCTCCGCT ATGAGGTAGA TAAGGAATCT ACGTTAAAAC GATCTTCTAC AACCCAATAT

+ ATATGTATCA CCCCGTCAAG CACAGCTGAT CTAGTACATG CTTTATTGAG GATCAGAGTG AGATCGAAAG
- TATACATAGT GGGGCAGTTC GTGTCGACTA GATCATGTAC GAAATAACTC CTAGTCTCAC TCTAGCTTTC

+ TACCATGGAG AATGGAAATT ATCATAATCA TGTCCTGGTG TATTTGGAGG AGTAGGAATA ACTGGATATT
- ATGGTACCTC TTACCTTTAA TAGTATTAGT ACAGGACCAC ATAAACCTCC TCATCCTTAT TGACCTATAA

+ CAACGAGATC CCGACGTAGG TGGAAACGTG CAGGGAGATG TTCAAGAGTG AAATGAGACT CATTTGCCAC
- GTTGCTCTAG GGCTGCATCC ACCTTTGCAC GTCCCTCTAC AAGTTCTCAC TTTACTCTGA GTAAACGGTG

+ AGGATTAAGT CAGAGGTAGA CGATAGAATT AGAAGTTGGA TACAACACTC TATAACATAG TGGTATGCTA
- TCCTAATTCA GTCTCCATCT GCTATCTTAA TCTTCAACCT ATGTTGTGAG ATATTGTATC ACCATACGAT

+ CTCCATCTTG TAAATTTTAC ATACTTATCC CAGAAACAAT AAAAATATTG TAGGCACATC CTACAGTAAC
- GAGGTAGAAC ATTTAAAATG TATGAATAGG GTCTTTGTTA TTTTTATAAC ATCCGTGTAG GATGTCATTG

+ TTACTATGGA AAAAACACGC TCTAACGTAT TGGATCCAAA CTTAGCTAAG GGCATCCTTG TCAGAAATAT
- AATGATACCT TTTTTGTGCG AGATTGCATA ACCTAGGTTT GAATCGATTC CCGTAGGAAC AGTCTTTATA

+ ACAGGTAACA CGTCATATGC TTTTTAGAAA AGAAAAAAAA GGCACTGATT CTACGGACGA TCGAATCCAA
- TGTCCATTGT GCAGTATACG AAAAATCTTT TCTTTTTTTT CCGTGACTAA GATGCCTGCT AGCTTAGGTT

+ TGGCGCATCT GTTTTATGTT AGCTAAAGTT GTTGGAAACG GCAGAAAATA GTAAAACAAA ATGAAAAACA
- ACCGCGTAGA CAAAATACAA TCGATTTCAA CAACCTTTGC CGTCTTTTAT CATTTTGTTT TACTTTTTGT

+ AGCCGTATGT AATCGGCAAT AAAATGAGCG GCATGTCTGT GTCGGGCGCC CACCGTATCC CTCTGCACCA
- TCGGCATACA TTAGCCGTTA TTTTACTCGC CGTACAGACA CAGCCCGCGG GTGGCATAGG GAGACGTGGT

+ GCTCCGCGCG TGCCCCGCTC GATCCCAGGA AGTACGGAGC TGTACGGCAC GCGACAGGGT ACGGAGGCGC
- CGAGGCGCGC ACGGGGCGAG CTAGGGTCCT TCATGCCTCG ACATGCCGTG CGCTGTCCCA TGCCTCCGCG

+ GCGTGGCCTC CCCGTTCCCG CCTCGTGGCC TGCAAGGTCA ATGGCGCCCG GATTGATCGA CGGCCGAGCT
- CGCACCGGAG GGGCAAGGGC GGAGCACCGG ACGTTCCAGT TACCGCGGGC CTAACTAGCT GCCGGCTCGA

+ AGTCTTGCAT CATGATGTGC GCTCCACAAT GGACATGCAA CTGCTCCTCC TATAAAAAGG CCTGCAGAAG
- TCAGAACGTA GTACTACACG CGAGGTGTTA CCTGTACGTT GACGAGGAGG ATATTTTTCC GGACGTCTTC

+ CTCATCGCAA GTCCACATC
- GAGTAGCGTT CAGGTGTAG
```

ABRE

TATC-box

8. ZmIDEF1.1

(none identified)

9. ZmIDEF2.1

```
+ TAAATTACAA TAACATACAA GAAATTTCCC CGTATCTAAT CCATTCACTC CCATGAAAAT ATTAGGTCAT
- ATTTAATGTT ATTGTATGTT CTTTAAAGGG GCATAGATTA GGTAAGTGAG GGTACTTTTA TAATCCAGTA

+ TTTCAATAGA GAGTTTTATC GCACTGTTTT TAAAACTGCC ATGTCAATAT TTGTCTAGGA AATAATGTAT
- AAAGTTATCT CTCAAAATAG CGTGACAAAA ATTTTGACGG TACAGTTATA AACAGATCCT TTATTACATA

+ CTAGAATTTC ATCCCATGAA ATTATTTGAT CTCTCATGAA ACTCTCTCAC ATCTCTTTTT ATTAAATTGC
- GATCTTAAAG TAGGGTACTT TAATAAACTA GAGAGTACTT TGAGAGAGTG TAGAGAAAAA TAATTTAACG

+ ATGCCATGCC ATCCTATTTA CTTATATGGT ATGCCATTTA ATGAGAGTGA AACTCCAACT GACAATGATT
- TACGGTACGG TAGGATAAAT GAATATACCA TACGGTAAAT TACTCTCACT TTGAGGTTGA CTGTTACTAA

+ TTACCATACT TCTTGTCAAC CAATCAGAGC AGCTCATTCA TGGTTATCAC ATGACCAAGC TTGAATGCTA
- AATGGTATGA AGAACAGTTG GTTAGTCTCG TCGAGTAAGT ACCAATAGTG TACTGGTTCG AACTTACGAT

+ TTTCTCCACT AATTTTTTGA GCTCTTGGAC TTTACACCAA ATCAACTTTG TCGAGTTCAC TATGATTTTT
- AAAGAGGTGA TTAAAAAACT CGAGAACCTG AAATGTGGTT TAGTTGAAAC AGCTCAAGTG ATACTAAAAA

+ TTCGGCTCTA AATATTTAGA TATCATTCAA CGCTATTCAT TGATGAAAAA TATATGAACA TGCACATATA
- AAGCCGAGAT TTATAAATCT ATAGTAAGTT GCGATAAGTA ACTACTTTTT ATATACTTGT ACGTGTATAT

+ CTATGTGATT TCTAAAAAAG TAGACTCCAT AGATAGTATC ATAGTATGCT CCCAAGTCCC AACCATTAGA
- GATACACTAA AGATTTTTTC ATCTGAGGTA TCTATCATAG TATCATACGA GGGTTCAGGG TTGGTAATCT

+ CTTTAACCAC CATTATTCTC ATACCTTAAA TGTAGGCTTG GATTGAGATC CAAGCAATTA CCAAACCAAT
- GAAATTGGTG GTAATAAGAG TATGGAATTT ACATCCGAAC CTAACTCTAG GTTCGTTAAT GGTTTGGTTA

+ TTTTTTATCC TCGACTACCC TACCATCACA TTGCAAGTGG AAATTTAATG TTGTCTTCAA TGGTATCTTT
- AAAAAATAGG AGCTGATGGG ATGGTAGTGT AACGTTCACC TTTAAATTAC AACAGAAGTT ACCATAGAAA

+ TATATTTGTC GTTTAGAGAT TTTCTAAAAG TTTTTTCTCT ATATTTTATT TCTCTTTAAC GATATTCTAT
- ATATAAACAG CAAATCTCTA AAAGATTTTC AAAAAAGAGA TATAAAATAA AGAGAAATTG CTATAAGATA

+ ATTCACATCC TTTGGACATT AAAGATCTAT ATTAAAACAT ATTTTACTTG AAAACTAGAG AAACCTTTAT
- TAAGTGTAGG AAACCTGTAA TTTCTAGATA TAATTTTGTA TAAAATGAAC TTTTGATCTC TTTGGAAATA

+ CGTGTCTGGT TAGAGTCACG GCCGACTCCG TGGAAATTTG GAAACCACGG TCACGTACTG CTGGGACTGG
- GCACAGACCA ATCTCAGTGC CGGCTGAGGC ACCTTTAAAC CTTTGGTGCC AGTGCATGAC GACCCTGACC

+ GAAGCTTCCT GTCCAGAATG CTGCGGCCTG CGGGAGTTGG AAGCAAAGAA ATAGAAAAAC CCATTGGTCC
- CTTCGAAGGA CAGGTCTTAC GACGCCGGAC GCCCTCAACC TTCGTTTCTT TATCTTTTTG GGTAACCAGG

+ CGCTCGCCAG TGTCCCGGT
- GCGAGCGGTC ACAGGGCCA
```

ABRE3a

ABRE4

10. ZmIRO2.1

```
+ GTGGATGCGG CGCGGCCGCG GCAGAACGTG CGGCGCGGCG CGTGCGCGGC TGGGGCGGCT ACGGGATGTG
- CACCTACGCC GCGCCGGCGC CGTCTTGCAC GCCGCGCCGC GCACGCGCCG ACCCCGCCGA TGCCCTACAC

+ GATGCGATGC GGCTGCGGGC GCGGCGCGTG CGCGGATGGG GCGGCTGCGG GCGTATGCGG GATGCGGAGC
- CTACGCTACG CCGACGCCCG CGCCGCGCAC GCGCCTACCC CGCCGACGCC CGCATACGCC CTACGCCTCG

+ GTCGCGGCTG CGGGCGCGGT AGAACATGCG CCAAGCGTTA GTTGGACGCC TACATTACTA ACATAATTAG
- CAGCGCCGAC GCCCGCGCCA TCTTGTACGC GGTTCGCAAT CAACCTGCGG ATGTAATGAT TGTATTAATC

+ TAGTAGAGAT TCACTACCCC TCTGTGTCAC CGTTAACCGT TTCCTACAAC TCCAGTGAAG CAGTGACGAC
- ATCATCTCTA AGTGATGGGG AGACACAGTG GCAATTGGCA AAGGATGTTG AGGTCACTTC GTCACTGCTG

+ CTAGCAGAGC GGCGCTGGTA ACATGTGGGC TGTGGCCCCC GTGGCTGGAC AGCACGGGGA GAAGGGCTGT
- GATCGTCTCG CCGCGACCAT TGTACACCCG ACACCGGGGG CACCGACCTG TCGTGCCCCT CTTCCCGACA

+ CGGGCACACG CCGGCACGAG GCGCTCCCGT TCCTCCACGC TCGCCTAGGG TGTCGACACC CTCACGCGAC
- GCCCGTGTGC GGCCGTGCTC CGCGAGGGCA AGGAGGTGCG AGCGGATCCC ACAGCTGTGG GAGTGCGCTG

+ GTACTAGTAC TCTACGTGTC TCGATTAGTA TTAAATAATG CCTCCGGGTT TTAGTATATT AGACAATAAT
- CATGATCATG AGATGCACAG AGCTAATCAT AATTTATTAC GGAGGCCCAA AATCATATAA TCTGTTATTA

+ AGTATAGTTA CAGGCGCCCT GATCACACAC TTCAATTCTC ACAGCTAGCC AGACGCTGAG CTAGGCTGCA
- TCATATCAAT GTCCGCGGGA CTAGTGTGTG AAGTTAAGAG TGTCGATCGG TCTGCGACTC GATCCGACGT

+ TGGATTAATA TGCATCTTCC CTCAACTCTT CTCATTAGCC CTGACGTCAT ATCACCGTTT TATTTAGCTT
- ACCTAATTAT ACGTAGAAGG GAGTTGAGAA GAGTAATCGG GACTGCAGTA TAGTGGCAAA ATAAATCGAA

+ AATGGGGTTT TATGGACATT AAATAATCTA ACACGACATA GGATTTTATG AGATGGAATG AGTCCTTGTC
- TTACCCCAAA ATACCTGTAA TTTATTAGAT TGTGCTGTAT CCTAAAATAC TCTACCTTAC TCAGGAACAG

+ CATTCAGTAC GGTTTTGTAG ACGACTCGTA TTTTCTTTCC TTTAAAAAAA CATTGCTGCA CCATCAAAGG
- GTAAGTCATG CCAAAACATC TGCTGAGCAT AAAAGAAAGG AAATTTTTTT GTAACGACGT GGTAGTTTCC

+ ACGGTTATGT AGCAGCTTAT TATTAAGTGT AAATAAAACT TGTCCTCCTA CATTGGGGAG TTTAGAAAAG
- TGCCAATACA TCGTCGAATA ATAATTCACA TTTATTTTGA ACAGGAGGAT GTAACCCCTC AAATCTTTTC

+ CTAGGAACAT GAGATGACAT CCTATACTGT AGGCTTGTTG CTTTCGTACA TACTGAAACT AGACCAACCT
- GATCCTTGTA CTCTACTGTA GGATATGACA TCCGAACAAC GAAAGCATGT ATGACTTTGA TCTGGTTGGA

+ TTCGTATAGC TCTAGGTACT CGACCACCAG AGATTTTTAT TATATATAGT TAACAAACAT TGCCTGCTGC
- AAGCATATCG AGATCCATGA GCTGGTGGTC TCTAAAAATA ATATATATCA ATTGTTTGTA ACGGACGACG

+ TAATCATCTA TCCATAAAA
- ATTAGTAGAT AGGTATTTT
```

ABRE

ABRE4

AT~ABRE

GC-motif

11. ZmIRO2.2

```
+ TATAGCATGC ACACACTAAA AAATATGTAT GTTGGCAGAA TTATTAGTCA AGAATTTAAA GAGTTGACTT
- ATATCGTACG TGTGTGATTT TTTATACATA CAACCGTCTT AATAATCAGT TCTTAAATTT CTCAACTGAA

+ TTTTAAAAAA AATTGGGATT GAGGGAGTAA TATTTTATAT ACAAAGTGTA TGTATGGTTA TTTTCGGTAA
- AAAATTTTTT TTAACCCTAA CTCCCTCATT ATAAAATATA TGTTTCACAT ACATACCAAT AAAAGCCATT

+ TCTTTTTTTT TAATCATATG AGCTAGCGCT TTAAAAGGAC CATTACAAAC ACCCCAAGTA TCTTTGAATG
- AGAAAAAAAA ATTAGTATAC TCGATCGCGA AATTTTCCTG GTAATGTTTG TGGGGTTCAT AGAAACTTAC

+ AATAATAAAA ATATATTTTG ACATATAAAT ATTATGTACT GTTTTTTATA ATCTTAGTTA AACTTTAAAA
- TTATTATTTT TATATAAAAC TGTATATTTA TAATACATGA CAAAAAATAT TAGAATCAAT TTGAAATTTT

+ ACTTCACGTT ACTTTAAACC TAAAATGTCA TGTTTTTTGA ACAGACGGAG TATGGCTCTA GGCCGGCCTG
- TGAAGTGCAA TGAAATTTGG ATTTTACAGT ACAAAAAACT TGTCTGCCTC ATACCGAGAT CCGGCCGGAC

+ ATTATCAGTT CTTGCTTTAG GGCATGTTTG GATGAGCTAA AGCAAAAGTG AGTAAAGTTT AGTCATTTAG
- TAATAGTCAA GAACGAAATC CCGTACAAAC CTACTCGATT TCGTTTTCAC TCATTTCAAA TCAGTAAATC

+ AAAATAAAGA TCCAAACAGA AAGAGTTGAA AAAGTTAAAA GTGACTAACT TGCCCTTAAT TAGTCACCTC
- TTTTATTTCT AGGTTTGTCT TTCTCAACTT TTTCAATTTT CACTGATTGA ACGGGAATTA ATCAGTGGAG

+ AATCTCAATC CAAACATGCT CCTTTAGGGC TTGTTCGGTA CTAGCCCAAT CTATATGAAT TAAGGACTAG
- TTAGAGTTAG GTTTGTACGA GGAAATCCCG AACAAGCCAT GATCGGGTTA GATATACTTA ATTCCTGATC

+ TTTTGATACT TTGGGATTAG AGTGTTTTGG AGGGATTGGA GAGGGTATAA ATCCCTAATA GATCAAATAC
- AAAACTATGA AACCCTAATC TCACAAAACC TCCCTAACCT CTCCCATATT TAGGGATTAT CTAGTTTATG

+ TCTTTCAATA CATCTCAATC CACTCTAATC TCACTCATTA CTAGAGTCCC CAAACTAGGC CTAAGGGGGA
- AGAAAGTTAT GTAGAGTTAG GTGAGATTAG AGTGAGTAAT GATCTCAGGG GTTTGATCCG GATTCCCCCT

+ TTGAGGGAGT TTCAATCCCT AGTAAGTCAA AACCTCTCCG GTCCATATCA GTCCACTTCA ATTGATATGG
- AACTCCCTCA AAGTTAGGGA TCATTCAGTT TTGGAGAGGC CAGGTATAGT CAGGTGAAGT TAACTATACC

+ ATTGAAAATA ACCCAGCATG CTCTTACCGA AAATTCTTGG CCGGCCAACT CTAGACCATG CATCCTGGCC
- TAACTTTTAT TGGGTCGTAC GAGAATGGCT TTTAAGAACC GGCCGGTTGA GATCTGGTAC GTAGGACCGG

+ TGTCGTATTG TAAAAGCTAG CAGGTACAGT ACTTTTTACT GCATGTGATA ATGTAGGCAG TTCACAGTCT
- ACAGCATAAC ATTTTCGATC GTCCATGTCA TGAAAAATGA CGTACACTAT TACATCCGTC AAGTGTCAGA

+ CTGTGGGACA TAGTGACGAC ACCTACCCGT GTCCACCATC TCAACCCTAG CTATATAAAT AGTAGGGAAC
- GACACCCTGT ATCACTGCTG TGGATGGGCA CAGGTGGTAG AGTTGGGATC GATATATTTA TCATCCCTTG

+ AAGTCGCCTC TTTGAAACT
- TTCAGCGGAG AAACTTTGA
```

ABRE

12. ZmIRO3

```
+ AAGAGATGAC AAAAGATATG AGGAAACAAG TGTACCAAGC GTTGTTGGCT AGAAGCAATA ACGGGAGACT
- TTCTCTACTG TTTTCTATAC TCCTTTGTTC ACATGGTTCG CAACAACCGA TCTTCGTTAT TGCCCTCTGA

+ CCACAAGAAA GATACCCAAA TTGTTGCCAA CCAATTTGAT CTCCACCTTC GGTCAGTGCA GCGCGTATGG
- GGTGTTCTTT CTATGGGTTT AACAACGGTT GGTTAAACTA GAGGTGGAAG CCAGTCACGT CGCGCATACC

+ AACAGAGGTA AAATGCAACT TGCAAACTCG GTGCCGGTTG TGGTTGCTAG TCTAAAAAAG GGAAGAGTTG
- TTGTCTCCAT TTTACGTTGA ACGTTTGAGC CACGGCCAAC ACCAACGATC AGATTTTTTC CCTTCTCAAC

+ GTCGTAAAGC AATTCCTCTG CTGCTGAGTC CGGCACGAGC TCGAAGGAGT CCAGCGTGAG CTCGAAGGAG
- CAGCATTTCG TTAAGGAGAC GACGACTCAG GCCGTGCTCG AGCTTCCTCA GGTCGCACTC GAGCTTCCTC

+ TCCGGCGCGA CCTCGAAGGA GTCCAGCACG AGCTCGAAGG AGTCCAACGT GGATTCTGAG TCAGACGCGA
- AGGCCGCGCT GGAGCTTCCT CAGGTCGTGC TCGAGCTTCC TCAGGTTGCA CCTAAGACTC AGTCTGCGCT

+ CGGAGTCCAG TGACATCTCG GAGTCCAGCG ACATCTCGGA GTCCAGCGAG GACGAGACGT CATCCTTGGC
- GCCTCAGGTC ACTGTAGAGC CTCAGGTCGC TGTAGAGCCT CAGGTCGCTC CTGCTCTGCA GTAGGAACCG

+ CACGGCGAGC GCAGAGTCGG AGTCCATGGC GAGGCGCGAG CAAGGAAAGC GAATGCGAGA CGCGAGCAGA
- GTGCCGCTCG CGTCTCAGCC TCAGGTACCG CTCCGCGCTC GTTCCTTTCG CTTACGCTCT GCGCTCGTCT

+ GTCGTGCGGT GTTTGCATGT CAGGCGCGCG GTGTTTGCAT GTTTGGCGTG GGCGTTAATG CATCACGTTG
- CAGCACGCCA CAAACGTACA GTCCGCGCGC CACAAACGTA CAAACCGCAC CCGCAATTAC GTAGTGCAAC

+ GTTTCCTTGC ATCGTGCGGT GTTTGCATGC GAGACGGAGT AATAATGCAT GGCGTGCGGT GTTAATGCAT
- CAAAGGAACG TAGCACGCCA CAAACGTACG CTCTGCCTCA TTATTACGTA CCGCACGCCA CAATTACGTA

+ GGCAACGGGC GACGCAGAAA ACGAAGCAAC GGGCGACGCA ATTACTCAGG GGCAGTGACG TCCAAATTCT
- CCGTTGCCCG CTGCGTCTTT TGCTTCGTTG CCCGCTGCGT TAATGAGTCC CCGTCACTGC AGGTTTAAGA

+ AGACCCATGC GTGCTACGAT TTCTTTTGTG CACGTTCGGT CACACGCAGA ATGACTTCCT TTACTGTACC
- TCTGGGTACG CACGATGCTA AAGAAAACAC GTGCAAGCCA GTGTGCGTCT TACTGAAGGA AATGACATGG

+ GGAGGGAGTA CATGTGATCT CCGGGCATGT GGTTTATTTT ACTCTGGTCT CTGGGACTCT CATGATGATG
- CCTCCCTCAT GTACACTAGA GGCCCGTACA CCAAATAAAA TGAGACCAGA GACCCTGAGA GTACTACTAC

+ ATCATGTCAG CGGTGCCAAC GATTGTGGAT CGCGATGACA CGACAGCGTA GGAGATGTTG ATGGGCTGAG
- TAGTACAGTC GCCACGGTTG CTAACACCTA GCGCTACTGT GCTGTCGCAT CCTCTACAAC TACCCGACTC

+ TCGGACGAGA CGCCAGCAGG ATGGCCCTTG GCGCGAGCCA GATGCCAACA GCCCTGTGGA TGTGGGTGGC
- AGCCTGCTCT GCGGTCGTCC TACCGGGAAC CGCGCTCGGT CTACGGTTGT CGGGACACCT ACACCCACCG

+ AGCGTGGAGG CTCGTGGCC
- TCGCACCTCC GAGCACCGG
```

ABRE

13. ZmFIT2

```
+ AGAAAGATAG TCGGTCGAGT GAGCTATAAA AAGAGGTGTG TTTTATTTTC AACTCATAAT TTTCTGCAGT
- TCTTTCTATC AGCCAGCTCA CTCGATATTT TTCTCCACAC AAAATAAAAG TTGAGTATTA AAAGACGTCA

+ TTAATATCTC GTACGTGGGT CATATATCTA CTTTAATTTT AAATTTATTT TTTATGGGGA TGGTCCACTT
- AATTATAGAG CATGCACCCA GTATATAGAT GAAATTAAAA TTTAAATAAA AAATACCCCT ACCAGGTGAA

+ TAATTTTAAA TTTATATTTT TTTGTATGAG GGTCCATCAT ATTGGTTTGC CATTAGGCCC TCACGTGACG
- ATTAAAATTT AAATATAAAA AAACATACTC CCAGGTAGTA TAACCAAACG GTAATCCGGG AGTGCACTGC

+ TACGGTTGTT GCACTATATC ATTAAATATG ATTGATGTGT CGTCGCAACG CATATGTATT GTATTAATTC
- ATGCCAACAA CGTGATATAG TAATTTATAC TAACTACACA GCAGCGTTGC GTATACATAA CATAATTAAG

+ TACAACATAA AGCACCGGTG TTTCTTATTT CCACCGTTCC ACAGTCGCTC TGTGTCACCC TTTCTACATA
- ATGTTGTATT TCGTGGCCAC AAAGAATAAA GGTGGCAAGG TGTCAGCGAG ACACAGTGGG AAAGATGTAT

+ AGGTAAAATT GTACATTAGT GTGGGTTTGA GCATTGATTG TTGGATGTAC ATTCGCACCC ACTTAACCTG
- TCCATTTTAA CATGTAATCA CACCCAAACT CGTAACTAAC AACCTACATG TAAGCGTGGG TGAATTGGAC

+ TAGACCACAT ATTTTTGTTA CCCTATAGAT ATATTCCCTT CCCCTACCAC TGAGTTGTTA CTTACCCTAT
- ATCTGGTGTA TAAAAACAAT GGGATATCTA TATAAGGGAA GGGGATGGTG ACTCAACAAT GAATGGGATA

+ AGATAGATCC TGAAAACTAC TCAAACTCTT ATTAGTGGAT CGTTACCTCA CAGATAAGCC GTAGAAAACC
- TCTATCTAGG ACTTTTGATG AGTTTGAGAA TAATCACCTA GCAATGGAGT GTCTATTCGG CATCTTTTGG

+ TTCCAAGCCC AACACGACAG GAAATTAGAT TTAACGTATT GCTCTCACAT AGTATCATCT CGCTAGGTAA
- AAGGTTCGGG TTGTGCTGTC CTTTAATCTA AATTGCATAA CGAGAGTGTA TCATAGTAGA GCGATCCATT

+ AGTAAGACAA ACAATCGAAA TAGTTATAAA AAAAGGTGTC GAACTCATTT TTAATCCATA ACTTACTAGC
- TCATTCTGTT TGTTAGCTTT ATCAATATTT TTTTCCACAG CTTGAGTAAA AATTAGGTAT TGAATGATCG

+ GCGTATGTGT ACAAATTAGC TCAAGGAAAA CGGAACTATG TAATTCGCCA AGGATTTATT TTTGGATATA
- CGCATACACA TGTTTAATCG AGTTCCTTTT GCCTTGATAC ATTAAGCGGT TCCTAAATAA AAACCTATAT

+ TAATATAAAT AGGCTGACGG CTTGTTTCGT GACAACCTGT TTCCGGTACA GAGTTAGCAG CCCACACCTG
- ATTATATTTA TCCGACTGCC GAACAAAGCA CTGTTGGACA AAGGCCATGT CTCAATCGTC GGGTGTGGAC

+ GTTTGTTCCA TCCCACTACT ATTCCAATCG TGTCGTCCAG TGGTTGAGAG GACGGCACAA ATGGAACCCT
- CAAACAAGGT AGGGTGATGA TAAGGTTAGC ACAGCAGGTC ACCAACTCTC CTGCCGTGTT TACCTTGGGA

+ AGCTAATACG GAGTAGTATC TTATTAGTCC GTTTCAGACT GGAGCTGCTA TATATATAAC GAGCTCGCCA
- TCGATTATGC CTCATCATAG AATAATCAGG CAAAGTCTGA CCTCGACGAT ATATATATTG CTCGAGCGGT

+ CTTGCGGTCT CGGCCTCGA
- GAACGCCAGA GCCGGAGCT
```

ABRE4

ABRE3a

ABRE

14. ZmFER1

```
+ GGCCAATCTG GTTCAGAGCG CGCGTGAGGT CGCCACGCGC TAGTGAGCTG AGTCATCAGT TGCTAGCGAG
- CCGGTTAGAC CAAGTCTCGC GCGCACTCCA GCGGTGCGCG ATCACTCGAC TCAGTAGTCA ACGATCGCTC

+ ATCTGAAGGT ACAGCCAACA ACCATTCAGA CGGGTGCGGT GGTCCTTCAC TCCAACAAAC AGCCTTGGAA
- TAGACTTCCA TGTCGGTTGT TGGTAAGTCT GCCCACGCCA CCAGGAAGTG AGGTTGTTTG TCGGAACCTT

+ TTCTATGTGT GTCCTGTCGC AACCACGAAT CTTCATCTTA TCTCCTCCAT CCTTGCTCGG GTCATCATCA
- AAGATACACA CAGGACAGCG TTGGTGCTTA GAAGTAGAAT AGAGGAGGTA GGAACGAGCC CAGTAGTAGT

+ GAGGCGTGTG CCGGCCGCAC GACCACTTTT TTTATTTTAT TTTATTTTAA ATATACGTCA GGTTTGTTGA
- CTCCGCACAC GGCCGGCGTG CTGGTGAAAA AAATAAAATA AAATAAAATT TATATGCAGT CCAAACAACT

+ AATAATAACG TGCTGCCGTT AATTAGATGA ACTGGACGAT ATAAATTCCA ACACCTACAA GTCTCGTGGT
- TTATTATTGC ACGACGGCAA TTAATCTACT TGACCTGCTA TATTTAAGGT TGTGGATGTT CAGAGCACCA

+ TATCCTTTGC TGCTCTGCAT TTACATTGAA TACTAGGGGT CTGTTTAGTT TAGCTTCCTA GACCGGATTC
- ATAGGAAACG ACGAGACGTA AATGTAACTT ATGATCCCCA GACAAATCAA ATCGAAGGAT CTGGCCTAAG

+ GTTGTAAAAA ATCTGAATTA ACTATACAAC AGAATAGATT TTTCATATTC ATCTCTTCCA TCTTACAAAA
- CAACATTTTT TAGACTTAAT TGATATGTTG TCTTATCTAA AAAGTATAAG TAGAGAAGGT AGAATGTTTT

+ ATGTCACGTT CTTATAAAAT CTATAGTTGA CAGTTATTTC AATCAAACAA AATAAAGATT TTCCACAATT
- TACAGTGCAA GAATATTTTA GATATCAACT GTCAATAAAG TTAGTTTGTT TTATTTCTAA AAGGTGTTAA

+ GTTAATGTTC GAGGAGATTT TCAACATTCT CAACCAAAGG GGTGGTGTCT GTATGCACCA CTTCTCCACC
- CAATTACAAG CTCCTCTAAA AGTTGTAAGA GTTGGTTTCC CCACCACAGA CATACGTGGT GAAGAGGTGG

+ ACCAAATTTA AAGCCATTCA AAAAAAATTA TGTCTGTGAC TGCACAAAGT TTGGTGAAAT TGATGAAACG
- TGGTTTAAAT TTCGGTAAGT TTTTTTTAAT ACAGACACTG ACGTGTTTCA AACCACTTTA ACTACTTTGC

+ GGCCATCCGA AGCTTCGGAA ACTATGAAAC TGAAGTTGCA AACTTTAAAA CATTTTGATA AGTCATTTTT
- CCGGTAGGCT TCGAAGCCTT TGATACTTTG ACTTCAACGT TTGAAATTTT GTAAAACTAT TCAGTAAAAA

+ ATTTAAATTA TTTTTAAAAA CTATTTAAAT TTATATTATA CACCACGGCT TCACGTCAAA ACTAGAACCT
- TAAATTTAAT AAAAATTTTT GATAAATTTA AATATAATAT GTGGTGCCGA AGTGCAGTTT TGATCTTGGA

+ AGAACAATGC CAGACTCCTT ATGGTATCCC TGCCTCATGG GAAAATAAGT TCTAGGACAA GGGGAAATTT
- TCTTGTTACG GTCTGAGGAA TACCATAGGG ACGGAGTACC CTTTTATTCA AGATCCTGTT CCCCTTTAAA

+ GAGTTTATTT AGGGCTAGTT TGGGAACACT AATGTTTCAT GAGATTTTCA TTTTTCCAAG GGAAATTAAT
- CTCAAATAAA TCCCGATCAA ACCCTTGTGA TTACAAAGTA CTCTAAAAGT AAAAAGGTTC CCTTTAATTA

+ TTATTTTCCA TTGGGAAAA
- AATAAAAGGT AACCCTTTT
```

ABRE

GARE-motif

15. ZmFER2

A. T001

B. T002

```
+ CAGGTCCTAC TTCCGGCGGA CGCTGGAGCC GAGAGGCATA CGCGTCCAGA TACTCAACAT CACGGAGCTG
- GTCCAGGATG AAGGCCGCCT GCGACCTCGG CTCTCCGTAT GCGCAGGTCT ATGAGTTGTA GTGCCTCGAC

+ TCCGACTACC GCAAGGACGG GCATCCCACG GTGTTCAGGA GGCAGTTCGT TCCCCTGACC AAGGAGCAGA
- AGGCTGATGG CGTTCCTGCC CGTAGGGTGC CACAAGTCCT CCGTCAAGCA AGGGGACTGG TTCCTCGTCT

+ TCGCGGACCC GGCCAGCTAC GCGGACTGCA CGCACTGGTG CCTCCCAGGC GTCCCCGACG TCTGGAACGA
- AGCGCCTGGG CCGGTCGATG CGCCTGACGT GCGTGACCAC GGAGGGTCCG CAGGGGCTGC AGACCTTGCT

+ GTTCCTGTAT GGCTACCTCA CGCAGCAGAG CAAATGACGA TGCATTATTA CAAGAATGGC CTTGTTATAT
- CAAGGACATA CCGATGGAGT GCGTCGTCTC GTTTACTGCT ACGTAATAAT GTTCTTACCG GAACAATATA

+ GTAGTAGTGT ACAGTGCAGT ACAGATTGCC AGCTGCTCCG TTTAGATTAT TAGGGATGGA CCTGCTGTTA
- CATCATCACA TGTCACGTCA TGTCTAACGG TCGACGAGGC AAATCTAATA ATCCCTACCT GGACGACAAT

+ CATGTAGAGA GAGACAGACA GGAGATCTGT CTTGCCCAAC ATTAATTCTA GTATCTGTAA ATCTTGTTCT
- GTACATCTCT CTCTGTCTGT CCTCTAGACA GAACGGGTTG TAATTAAGAT CATAGACATT TAGAACAAGA

+ TCTTGTCCAA CATTAATTCT AGGATCTGCT GCTAACCATT TTTTTTGGAT TTATGTAAAC TGAGTGATTA
- AGAACAGGTT GTAATTAAGA TCCTAGACGA CGATTGGTAA AAAAAACCTA AATACATTTG ACTCACTAAT

+ TCTGAAATAG AATGATCTGA GTTTGCTGTT CCAGTACTGA GCAAGTAAAA AAACCAAAAA AACAAATCGC
- AGACTTTATC TTACTAGACT CAAACGACAA GGTCATGACT CGTTCATTTT TTTGGTTTTT TTGTTTAGCG

+ CCACCGTGGG GCTCGAACCC ACGACCACAA GGTTAAGAGC CTTGCGCTCT ACCGACTGAG CTAGACGGGC
- GGTGGCACCC CGAGCTTGGG TGCTGGTGTT CCAATTCTCG GAACGCGAGA TGGCTGACTC GATCTGCCCG

+ TTGTTTTCCC TTTTTTTTTC TTTGGCTATA AATTGGATCC GCATCAGTCA GCTCAACACT TGTTACGGAG
- AACAAAAGGG AAAAAAAAAG AAACCGATAT TTAACCTAGG CGTAGTCAGT CGAGTTGTGA ACAATGCCTC

+ CAACTAAACA TAGAGAGAGA GAGAGAGGGT GGATGACGAG GACGGCCTAC TGCTTTCTTT GAGACAGCAT
- GTTGATTTGT ATCTCTCTCT CTCTCTCCCA CCTACTGCTC CTGCCGGATG ACGAAAGAAA CTCTGTCGTA

+ GCATGCATGG CCTGAGCTTG CTTTGAGCCC TCCTCGCTCG CTCGCTCACC TTCTTATCTC CTCCATCCAT
- CGTACGTACC GGACTCGAAC GAAACTCGGG AGGAGCGAGC GAGCGAGTGG AAGAATAGAG GAGGTAGGTA

+ CCAAAAATAT CTCCATGCGC TCAGACGGGG ACGAGACGAC GGGAGAAGGA GGAGGAGGGC GCCCGCCCGC
- GGTTTTTATA GAGGTACGCG AGTCTGCCCC TGCTCTGCTG CCCTCTTCCT CCTCCTCCCG CGGGCGGGCG

+ CACGCGCCTC CTCCAAATCC CAGCCGTCCA CGTGTCCCAG TCCGCGACCC CCACGCGCCT ATATCCATCC
- GTGCGCGGAG GAGGTTTAGG GTCGGCAGGT GCACAGGGTC AGGCGCTGGG GGTGCGCGGA TATAGGTAGG

+ ATCATCCTAT TCCTATCCC
- TAGTAGGATA AGGATAGGG
```

ABRE

RY-element

Manual search of *cis*-acting elements not provided by PlantCARE

Key

IDE1 element: 5’-CATGC-3' (Kobayashi et al., 2009)

IDE2 element: 5’-CA(A/C)G(T/C)(T/C/A)(T/C/A)-3’ (Kobayashi and Nishizawa, 2012)

E-box: 5′-CANNTG-3′ (Carey-Fung et al., 2022)

RY element: 5’-CATGCA-3' (Kobayashi et al., 2009)

1. ZmNAS1

GGAGAATGATAGTCCAATGAAATTTGTTAGGGTGAGTGGAGTTCGTTCGTTGCATATTCAAACATACATTCTA
GTTACATAATAAAAAATTGACGTTTTGTTCTCTTATATGTCTTATATGTAGGATACAAAGATTCTTCGGATAAAC
TTTATTATCGAACTGTTAAGTTACGAGGAAAATTCGTGCAGACATGCCATCCCAGCAAATATACAACAGAGAG
TTAACAATATTGTTAAAAAATATTAGATTAGTGTACAAGGTGTCAACATATTTACTTATGATTAAGCATTATATA
TTTACTCTTTAGAAATTATTTATGTGATTCTATTTCAATCTATTTTGGTAGACATATTTATATCGTTCTGTATCTA
TGTTTAATAATCACATATTATACCGTCACTATTGTACTCACGCCGCACGTCGTCAGGGCCCTAAATCCATTGG
AGGGTCGCAATTTGTGTTCCGTGGCATCGCATGGTAACGTTCCTAGTTATATATTGATAGCTGCAAGACGTA
CATCATGTCAACTTTAGAGAGAGAAAGACAAGAGATTGGTTTGCTAGCTCTTCCCTTTATTGTGATGATTGAG
GCCGGGAGCAGTTTTCTTAATTAAGATTTTTGAAAGAACTTGTATTTTGTTTTTAATCTTTTTATCAGTGCTCG
ACCAATGTCATGATGTGGACGTGGCAAACCCACTTCGGGAGGGGACAAAAAAAACAGGGCTGCCACTTTCA
GGGCGTGACGTGGACGTGTTGAAGATCCGGAACATTCTTCGTGAAACGTACACCGTCAAAATAGCGAGGCA
TGAAACTGGCCTTGGCCATGGACGCGTGAAGCGCGCCATGCGTTGGATATGTGGTCAATAAGTATATACAAT
ACAATGTTTAACAGAGCTGGTAGTACTGCTTCGGCACATTTTTGTCCACGCTTCATGAGAGACAAAAACACCT
GCACTTAAATTCACATGCTGCACTGAAGGCCCGATCACTGAGGAGCGAAC

IDE1 element (+ or – strand)

IDE2 element (+ or – strand)

E-box (+ or – strand)

2. ZmDMAS1

GGAGTGTTGGAATTTATACATATCCCCAGACTTTTGGTACTGTTGTATTTGCATTACTTCCAGCGATGCTTTCT
TAGGATTGTGGACCTATTGGTTCTTTAAACGTGGTGGCTGATTCGCTTCCTTCGACTGAGCTCGCATCTTCG
GTACGTTGTTTTCTTCTGACCTCGATGGTTCCCAATTTGGTTTCAGCTTACACGCGTCGAAGACCCGGGTAA
CTGTCAGGAAAACGAGTAGGATGAAAGCGAAGCTCGCGGTGCTTGGGCGCGAGCCGCCCGGCGTTACAAG
TTGCAATGCAACTCTGGTGGAGGCTATCGCAAGTCACTACCCATATGAGGCCACGTTCGTTTGATCCCAAAA
CCATAGGGATTAAGGGGGATTGGAAAGGATTAGAGAGGATTTTGACTCGTAGAGAATTTGATCCCCCTTCAA
TCCCTATGGATCAGCACAAAACGAACGGGGCCTGACGGAGGACGGGAGGAGGCTTGTTTGTCCTATAACG
GCGTCGCGGAGCCTCAGCCAGCATATGTTCTTGTTGGATGCCGGAGTAGCGGAGTGGGCGAATGGACTGC
ACGCTCTCATGCCATGCGGCACGCCTACGACTACGAGATGCACGGCGACGCTGACGCAGCGCGGGGCCC
GCGGGGACGGACGGCATGAACCCGTCCAGTGTCTCGGGTCTCAGAGCTACAAACAAAGCCGCATGGTGGC

ATGATCTGTGCCTGTCCCGGTGGTGAGTAGTGACGATCGCTGCCGCTTTTCCCCCATCGGCCGCCGCAGCA
TGACATCACATATCAGTGGACTGGAGCAGGCAACAGAAGATCTCGAACTCGAAGCGTGAAAACCACTGCTA
GAGCATTTCATGTTACAGCCCGCGAAAACGTAAAAAAAATCGAAGGAATCCGAGGAGAAAATCAGGCAAGTC
ATGGCTACCGGACCCCGAACGCTCCTCAGCTCCTTGGACGTCCAAAAAACAAACGCAAACTTTTGTTGTTTG
TAGG

IDE1 element (+ or – strand)

IDE2 element (+ or – strand)

E-box (+ or – strand)

3. **ZmTOM1**

TTGGATGGAGTAAACAACAACATCAGTTAGTGAAATCAAAAAAATATTATGCAGAGAGCAGAGACAATTAATA
AAAATCTTGAGATCTTTTTGATGGATAGTTTACGTATATATTGTTGTGAGCCGTCGCAAAACGTACTGACATTA
TATATTGTCCAATCGTCGAACCGACGACTGAAGGTTAATTGATAGTGATACTGGTGGCGCCCAGGCCGGCC
GGCCGTAGACTAGCATGCATGCATGCAGAGCTTGGTTAGTTTCCTGTGGTGTGACTTGGTCATCAATTAAGC
GAGAGGCAGCTATGCATATGTCATGTGTACATGCATGTGTGGGTGGAGCGATTGGCACATTTCACGTTGTCT
CCCTTGCTTCTTGCACTGTGCGTGCTCATTGTGCCAGGGTTTGACTCCGCATATATAAATAGCTTCCTCTCC
GTCAGCGCTGACACAGTACGTCTTACTAACCATATTATGATACTCAAATTACCTGCCCACCATCCCCTGCAAA
ACTAGCCACACATATATGTAAGTATGTGTATTTGTTTGCTGCAGCGCTTCTCCTTCAAGTACGTACGTGTCGC
CTGTGCCAATGGATAATTTGATTTGCCTCGGCATAAATATATACAAACTGTGTGGCCTAGCTAGCTTACCAAC
CGTCTTTTGTCCTTGTATACTATGACAACAAGTTGGTTATACTCCGCGCGCCTTGCATCAGGAAATCGACGTA
GTGCAAAAGCGGGCGGGAGGGCGGCGGCATGTGCATGCATGCCGCCCATCGGCGCGTACGCATATGCCA
CGCACGCTACACAGAGACGTGCGTACGTACGTAGAGCAGTCTCGCTCGTCCAGCTCGATCTACACAACATC
TGGCTGACTAGCTACATGCAGTGTGCATGTCGTCATTATTACTCACCTCGGTAGTAGCATTACAATGTATGTA
TGTATATATAACCCATGCAGGCAGCTACCACCCGGCCAAGAGCAGTATTCCAGGCCCAGGGT

IDE1 element (+ or – strand)

RY element (+ or – strand): additional sites to these predicted by PlantCARE, manually found

IDE2 element (+ or – strand)

E-box (+ or – strand)

4. **ZmYS1**

ACTTGCGTATTTGCTGTTGGCGGCTGCCGACCCGCTGCACGCCTGCACCTCCCTGGCTCCCTCCCCGGCTC
CCGCGCCGGTGCCTCTGCCTTACTGCGTGGGCCGCCTCCGTCGACCTCGTGCGCTCGCAGCTCGGCCTCG
TGCCATCGTCATCCGCGTGCGCGCCACCGCGTGGACCGCCTCCGTCGGCCTCGTTGTAATACTTGGCGGT
GTACACGTGGGACTAAAAGGAAAACCAGCAATAGTGACACATAGGGGTCAGGGGAACTAAAAGCACAACCC
AATGCCGACGAAGACAACGCATAATTTTCAGTGGCACCGTTAAGGAAAATTTATATATTTTAACGGCATTCTA
AAAGATCTCTTAAATTTATATGTCTAAAACAAATTAGAATGAAATTTATATGTCTAGACAAATTAGAATGACTTA
TAATCTAGAACAGGTACTACAAAAGTCGGTCCAGTGACTCCAGTAGGCTCTTCTGGACTAACGGTAATAGGA
CATGTTTTTTTATAGAATATACACAAGCATATTTGTTAACGAGCACTTAGCAGATATGAGTACTGGACTAACGA

TAATATGTCACGATTCCCCGATAAACTTCGTTCCAAAGAAGTCATAACCGTTTGGTTCACTAAATGTAACGTA
AATGATAATGATAACGGTTCACACTCGAATACCGGCGGTAACAAATTTGAATGAGACGATATCCATTTGTAGT
ATGATATCGATTACGATTGGACTTAAACAAACATGATTTAACGTTATCGGTTACCGATTACGTTACCAATACGT
GAACCAAACGGCACCTAACTTCCATCAATTTGTCCGTGAGATGAACTATATACAGGTATACAGACATGAAGG
CAAGAAAAATGCTGCACCGATCAAGTCGCTTTCCTTCACCATTCTCCCCCCTAACAACTCTCTCGACAGCTAA
TAATGCGACGACTTGGAAATGTCTTAGTATTTTTCTGCCACGGAAATAGTTAAGGGTTTG

IDE2 element (+ or – strand)

E-box (+ or – strand)

5. ZmNAS3

GATCTCTGCTGTAGCGTACACTAGCGTGTATGAACTCTAGTGCAGGACGTATATGTTCCTTGGGAATAGCGT
AAAGTAGAGAAAGGTAGATAGATAGATAGATATTCTTGGGGATCGGAATAAGTATATTTGAAGTAAAGGAAAT
ATGAGGCAACGACGACATGCATGGAACTTGAATTATTGTTATATGGGGCATCGTAGCTATGCTATATTATCGT
GACAGAAATGCACGGCCGTTCCTGACTTTTGAGGGGCCCAGGACAAAATTATAAACAGAGGCCCCAACACC
ATAAAAACTATTTTTTTACTATTATATATATCAACTAATTATACTTGATGTACCACAGAAAGACTTGAGCAGTAA
AAAATAGAAGTAAGATTATTACCTCAAATATATTGTAGAGATCAACTTAAAAATGTCTTCTAACGTTTCTAAATG
CAAAATCATCAATGATGGCTTCAATATTGACATCATCTAATAATTTCTTCTCAATACATAAAGTTGCTAACCCG
TCTAACCTTTCTTGAGAAATTATAGACCTTAAATAATTCTTCAATAACTTAAGCTTTGAGAAGCTCCTTTAAGCT
CATGCCACAGTTATTGATATAGTAAATAATATCTGATAAGCAACAAATATATTGGGATATCAATCCATTCTCCT
CAGAAACTCAAAAATATATTAGTATAGTAAATAATATCTGTTTCTATCCTATATTCCTATTAGACTTTGTGTGCG
GTGGGGCCCCTGGGAGATGGGGGCCCAGATCGGCCGCCCCTCCCGCCCTGCCTCAGGCACGGCCCTGCA
GAAATGAAATGGAACTTCACTCGCCAGTGCAAGTTGTACGCGTGCTGTGGCGACTGGCGACGACGACGACT
GCGACAAGGACGCCGTTGCGTGTGTGGAGAAAACAACGCAGAGGGCTTGTGGCGGGCACGCGTCTACAAG
ACAGGGACGCCATTATTCCCCCACCACCATATATATATAGTGTACTATGCCTCG

IDE1 element (+ or – strand)

IDE2 element (+ or – strand)

6. ZmIRT1

GCCTTCGTGCTGTTCTTCCTCTCCAACCTGCTGCCCCGCCCACGGTCGCAGCCGCGAGCCGACCGATGCT
CGTCGACGAGGGTACGGGGCGAGTCGGTCATGCTCTTGGTCTTTCTCCGTGCGTGCTATCGAGGAGGACA
TGGTGGCACCTGCCACGCTTAGGTTATCTTGGCGATTAGGAGTCCAGGCCTGAGATATTGGACAATCAAGA
CCCGTAGCGTGGCAGCAGTCGGCATATGCTACGGAGTTGGTGGTGGTGTTGTGTTGCTTCGGCGGGTCCA
GGGCTGGGAAGACATTCGCATTTTCTCGCCCTTCTCGGATTGGCGCCTGGTGCGGGTGCCTCTCGGTTTGG
AGCTGCTCCGGCTGGGCTTCTTTCTCCACTTGCATGCTCTCTCCCTATATATCTAGCGGTATACTACCTATGT
CATCTCCAGATGACCAGGTCTTCGTCGTGTCCTTCTCCGGCAACGAACAGGTATGTCCGCATCTTTCCTTCC
CCTCCACTCCTGCTCTACGAAACCTTGGAAGTGGGAGGGCAAGGGATTGGGGTCTCTGATTTGCTGTTTAT
GTTAACCGTGCGTTCAAAAAAAATTACGTGAAAAGTAGAGACAATCAATAAAAAAACTTGAGATCTTTTTGTG
GATAATTTATGTGGTTATTGTTGTGAGCCGTCGCAACGCACAGGCTAGTATGGTATTAAAGACACAGATCGG
CCTTCACATGAAAAATAGTGTGGAAATATAAGTGAGTTCTTCGTCTCTTATAAGTGAATACCAGTTAACGCTA
CTGAATAATGTTAGGATGTGTCCTCTACACTAGCCGATTAACAGTAACCGGGGTAGGGTTAATCCTAACCGT
CTAACCGCGCCCCTGATCGGGGGGCTATCCTAACCGTTGGTTGCAGCCCCCGGTCACACGACGCCATAAC

GTGTGCTGGAAGTGAAAGACCATCCAAGTTGCTCCAGGCCCTATATATATATGTGCAACCCCTACAACAGCC
G

IDE1 element (+ or – strand)

RY element (+ or – strand): additional sites to these predicted by PlantCARE, manually found

IDE2 element (+ or – strand)

E-box (+ or – strand)

7. ZmIRT2

ACATTAAACACTCGTTCATTCCTTAGACGTTGATCAATGCACATGGACTCCTACACTTGTGATAATTGCATACT
GCAGCACGAAGAGGCGATACTCCATCTATTCCTTAGATGCAATTTTGCTAGAAGATGTTGGGTTATAATATGT
ATCACCCCGTCAAGCACAGCTGATCTAGTACATGCTTTATTGAGGATCAGAGTGAGATCGAAAGTACCATGG
AGAATGGAAATTATCATAATCATGTCCTGGTGTATTTGGAGGAGTAGGAATAACTGGATATTCAACGAGATCC
CGACGTAGGTGGAAACGTGCAGGGAGATGTTCAAGAGTGAAATGAGACTCATTTGCCACAGGATTAAGTCA
GAGGTAGACGATAGAATTAGAAGTTGGATACAACACTCTATAACATAGTGGTATGCTACTCCATCTTGTAAAT
TTTACATACTTATCCCAGAAACAATAAAAATATTGTAGGCACATCCTACAGTAACTTACTATGGAAAAAACACG
CTCTAACGTATTGGATCCAAACTTAGCTAAGGGCATCCTTGTCAGAAATATACAGGTAACACGTCATATGCTT
TTTAGAAAAGAAAAAAAAGGCACTGATTCTACGGACGATCGAATCCAATGGCGCATCTGTTTTATGTTAGCTA
AAGTTGTTGGAAACGGCAGAAAATAGTAAAACAAAATGAAAAACAAGCCGTATGTAATCGGCAATAAAATGA
GCGGCATGTCTGTGTCGGGCGCCCACCGTATCCCTCTGCACCAGCTCCGCGCGTGCCCCGCTCGATCCCA
GGAAGTACGGAGCTGTACGGCACGCGACAGGGTACGGAGGCGCGCGTGGCCTCCCCGTTCCCGCCTCGT
GGCCTGCAAGGTCAATGGCGCCCGGATTGATCGACGGCCGAGCTAGTCTTGCATCATGATGTGCGCTCCAC
AATGGACATGCAACTGCTCCTCCTATAAAAAGGCCTGCAGAAGCTCATCGCAAGTCCACATCC

IDE1 element (+ or – strand)

RY element (+ or – strand): additional sites to these predicted by PlantCARE, manually found

IDE2 element (+ or – strand)

E-box (+ or – strand)

8. ZmIDEF1.1

TCCATTGCTCCTGCCCCTAGAATTGAAGGTTGGTTCATGTTTGGTGGAAGACTAAAAAGGACAAAGGAAGGA
GGCGTTGTATCAGTAGCACCGTTGCCTAGTTACATTAGCATCACCATCAGCATCAACCAAACAGTACAATAG
CTGCATTTCACAAACATATTATCGGAATACTTAATGGTCCTAGTTTTACAATACTTGCCTAGATTATTAGAATC
CTCGGATCTGTACTTTACCGGACGGTCACCAAAATACTTAGCTTAACATACTAGTAAAATATGAAATTCTTCTA
ACGATTTTTAGAATGCATACTACCCTCCACTTTAGTTTACGTATCATTGGTAGAGAACATTGCAGCAATTCTGA
CGATGAGTTTTTTTTCTTTCTAGAAAAAAAAGATTAAATTTGTTGAGACTTATGTTTGAGTAGGTGTATATAATA
CACTTCATTAGCATTGTATACTTTTAGATTAAAAAATGGGAGTGTACGCAACTTCCTAATGACAAATACAATAC

TTCATTTATTATGAATAAAAAATTGGATCCGAATGACTAGTCACTACATTTTTAATGGGGTATTTGCCGACTAA
ATTCTAATGTTTAAATGATAATTATTGGTGCTAAAAATAGTTTAATTATTATTAAATAATATGATGTAATTGAAG
GCGTGCGCGGCAGAATGGATAGTGATAATGGGTGCAAATCCTGGGAGGATATTTTTGTTCCTTGCCTTTACC
CTTTTTTTCCCCTCTCACGGTCGAATCGAAGATACGAAGAAGTCGGAGGAGGAAAAAAAAGCAAGGAATTCA
CCATCAGTACGTTCTTGCGGCGTGTAGAGAGAGAAGAGAACGGGTGACAGTAGCAAGCAGCCATAGCATAG
CAGGACCACTCCTGCGCTGCACTGCACCAGTCTGCAGGTGGGTGCGGTGGGGGTGGCCGGGTGGGGTGA
AGTGGAGAGAGAAAAGAGGAGGAGGTCGTCTGGCCCTTTCCCCTTCTTTGCCC

IDE2 element (+ or – strand)

E-box (+ or – strand)

9. **ZmIDEF2.1**

GTAAATTACAATAACATACAAGAAATTTCCCCGTATCTAATCCATTCACTCCCATGAAAATATTAGGTCATTTT
CAATAGAGAGTTTTATCGCACTGTTTTTAAAACTGCCATGTCAATATTTGTCTAGGAAATAATGTATCTAGAAT
TTCATCCCATGAAATTATTTGATCTCTCATGAAACTCTCTCACATCTCTTTTTATTAAATTGCATGCCATGCCAT
CCTATTTACTTATATGGTATGCCATTTAATGAGAGTGAAACTCCAACTGACAATGATTTTACCATACTTCTTGT
CAACCAATCAGAGCAGCTCATTCATGGTTATCACATGACCAAGCTTGAATGCTATTTCTCCACTAATTTTTTGA
GCTCTTGGACTTTACACCAAATCAACTTTGTCGAGTTCACTATGATTTTTTTCGGCTCTAAATATTTAGATATC
ATTCAACGCTATTCATTGATGAAAAATATATGAACATGCACATATACTATGTGATTTCTAAAAAAGTAGACTCC
ATAGATAGTATCATAGTATGCTCCCAAGTCCCAACCATTAGACTTTAACCACCATTATTCTCATACCTTAAATG
TAGGCTTGGATTGAGATCCAAGCAATTACCAAACCAATTTTTTTATCCTCGACTACCCTACCATCACATTGCA
AGTGGAAATTTAATGTTGTCTTCAATGGTATCTTTTATATTTGTCGTTTAGAGATTTTCTAAAAGTTTTTTCTCT
ATATTTTATTTCTCTTTAACGATATTCTATATTCACATCCTTTGGACATTAAAGATCTATATTAAAACATATTTTA
CTTGAAAACTAGAGAAACCTTTATCGTGTCTGGTTAGAGTCACGGCCGACTCCGTGGAAATTTGGAAACCAC
GGTCACGTACTGCTGGGACTGGGAAGCTTCCTGTCCAGAATGCTGCGGCCTGCGGGAGTTGGAAGCAAAG
AAATAGAAAAACCCATTGGTCCCGCTCGCCAGTGTCCCGGTA

IDE1 element (+ or – strand)

RY element (+ or – strand): additional sites to these predicted by PlantCARE, manually found

IDE2 element (+ or – strand)

E-box (+ or – strand)

10. ZmIRO2.1

TGTGGATGCGGCGCGGCCGCGGCAGAACGTGCGGCGCGGCGCGTGCGCGGCTGGGGCGGCTACGGGAT
GTGGATGCGATGCGGCTGCGGGCGCGGCGCGTGCGCGGATGGGGCGGCTGCGGGCGTATGCGGGATGC
GGAGCGTCGCGGCTGCGGGCGCGGTAGAACATGCGCCAAGCGTTAGTTGGACGCCTACATTACTAACATA
ATTAGTAGTAGAGATTCACTACCCCTCTGTGTCACCGTTAACCGTTTCCTACAACTCCAGTGAAGCAGTGAC
GACCTAGCAGAGCGGCGCTGGTAACATGTGGGCTGTGGCCCCCGTGGCTGGACAGCACGGGGAGAAGGG
CTGTCGGGCACACGCCGGCACGAGGCGCTCCCGTTCCTCCACGCTCGCCTAGGGTGTCGACACCCTCACG
CGACGTACTAGTACTCTACGTGTCTCGATTAGTATTAAATAATGCCTCCGGGTTTTAGTATATTAGACAATAAT

AGTATAGTTACAGGCGCCCTGATCACACACTTCAATTCTCACAGCTAGCCAGACGCTGAGCTAGGCTGCATG
GATTAATATGCATCTTCCCTCAACTCTTCTCATTAGCCCTGACGTCATATCACCGTTTTATTTAGCTTAATGGG
GTTTTATGGACATTAAATAATCTAACACGACATAGGATTTTATGAGATGGAATGAGTCCTTGTCCATTCAGTAC
GGTTTTGTAGACGACTCGTATTTTCTTTCCTTTAAAAAAACATTGCTGCACCATCAAAGGACGGTTATGTAGC
AGCTTATTATTAAGTGTAAATAAAACTTGTCCTCCTACATTGGGGAGTTTAGAAAAGCTAGGAACATGAGATG
ACATCCTATACTGTAGGCTTGTTGCTTTCGTACATACTGAAACTAGACCAACCTTTCGTATAGCTCTAGGTAC
TCGACCACCAGAGATTTTTATTATATATAGTTAACAAACATTGCCTGCTGCTAATCATCTATCCATAAAAT

IDE1 element (+ or – strand)

RY element (+ or – strand): additional sites to these predicted by PlantCARE, manually found

IDE2 element (+ or – strand)

E-box (+ or – strand)

11. ZmIRO2.2

ATATAGCATGCACACACTAAAAAATATGTATGTTGGCAGAATTATTAGTCAAGAATTTAAAGAGTTGACTTTTT
TAAAAAAAATTGGGATTGAGGGAGTAATATTTTATATACAAAGTGTATGTATGGTTATTTTCGGTAATCTTTTTT
TTTAATCATATGAGCTAGCGCTTTAAAAGGACCATTACAAACACCCCAAGTATCTTTGAATGAATAATAAAAAT
ATATTTTGACATATAAATATTATGTACTGTTTTTTATAATCTTAGTTAAACTTTAAAAACTTCACGTTACTTTAAA
CCTAAAATGTCATGTTTTTTGAACAGACGGAGTATGGCTCTAGGCCGGCCTGATTATCAGTTCTTGCTTTAGG
GCATGTTTGGATGAGCTAAAGCAAAAGTGAGTAAAGTTTAGTCATTTAGAAAATAAAGATCCAAACAGAAAGA
GTTGAAAAAGTTAAAAGTGACTAACTTGCCCTTAATTAGTCACCTCAATCTCAATCCAAACATGCTCCTTTAG
GGCTTGTTCGGTACTAGCCCAATCTATATGAATTAAGGACTAGTTTTGATACTTTGGGATTAGAGTGTTTTGG
AGGGATTGGAGAGGGTATAAATCCCTAATAGATCAAATACTCTTTCAATACATCTCAATCCACTCTAATCTCA
CTCATTACTAGAGTCCCCAAACTAGGCCTAAGGGGGATTGAGGGAGTTTCAATCCCTAGTAAGTCAAAACCT
CTCCGGTCCATATCAGTCCACTTCAATTGATATGGATTGAAAATAACCCAGCATGCTCTTACCGAAAATTCTT
GGCCGGCCAACTCTAGACCATGCATCCTGGCCTGTCGTATTGTAAAAGCTAGCAGGTACAGTACTTTTTACT
GCATGTGATAATGTAGGCAGTTCACAGTCTCTGTGGGACATAGTGACGACACCTACCCGTGTCCACCATCTC
AACCCTAGCTATATAAATAGTAGGGAACAAGTCGCCTCTTTGAAACTG

IDE1 element (+ or – strand)

RY element (+ or – strand): additional sites to these predicted by PlantCARE, manually found

IDE2 element (+ or – strand)

E-box (+ or – strand)

12. ZmIRO3

AAAGAGATGACAAAAGATATGAGGAAACAAGTGTACCAAGCGTTGTTGGCTAGAAGCAATAACGGGAGACTC
CACAAGAAAGATACCCAAATTGTTGCCAACCAATTTGATCTCCACCTTCGGTCAGTGCAGCGTATGGAAC

AGAGGTAAAATGCAACTTGCAAACTCGGTGCCGGTTGTGGTTGCTAGTCTAAAAAAGGGAAGAGTTGGTCGT
AAAGCAATTCCTCTGCTGCTGAGTCCGGCACGAGCTCGAAGGAGTCCAGCGTGAGCTCGAAGGAGTCCGG
CGCGACCTCGAAGGAGTCCAGCACGAGCTCGAAGGAGTCCAACGTGGATTCTGAGTCAGACGCGACGGAG
TCCAGTGACATCTCGGAGTCCAGCGACATCTCGGAGTCCAGCGAGGACGAGACGTCATCCTTGGCCACGG
CGAGCGCAGAGTCGGAGTCCATGGCGAGGCGCGAGCAAGGAAAGCGAATGCGAGACGCGAGCAGAGTCG
TGCGGTGTTTGCATGTCAGGCGCGCGGTGTTTGCATGTTTGGCGTGGGCGTTAATGCATCACGTTGGTTTC
CTTGCATCGTGCGGTGTTTGCATGCGAGACGGAGTAATAATGCATGGCGTGCGGTGTTAATGCATGGCAAC
GGGCGACGCAGAAAACGAAGCAACGGGCGACGCAATTACTCAGGGGCAGTGACGTCCAAATTCTAGACCC
ATGCGTGCTACGATTTCTTTTGTGCACGTTCGGTCACACGCAGAATGACTTCCTTTACTGTACCGGAGGGAG
TACATGTGATCTCCGGGCATGTGGTTTATTTTACTCTGGTCTCTGGGACTCTCATGATGATGATCATGTCAGC
GGTGCCAACGATTGTGGATCGCGATGACACGACAGCGTAGGAGATGTTGATGGGCTGAGTCGGACGAGAC
GCCAGCAGGATGGCCCTTGGCGCGAGCCAGATGCCAACAGCCCTGTGGATGTGGGTGGCAGCGTGGAGG
CTCGTGGCCG

IDE1 element (+ or – strand)

RY element (+ or – strand): additional sites to these predicted by PlantCARE, manually found

IDE2 element (+ or – strand)

E-box (+ or – strand)

13. ZmFIT2

AAGAAAGATAGTCGGTCGAGTGAGCTATAAAAAGAGGTGTGTTTTATTTTCAACTCATAATTTTCTGCAGTTTA
ATATCTCGTACGTGGGTCATATATCTACTTTAATTTTAAATTTATTTTTTATGGGGATGGTCCACTTTAATTTTA
AATTTATATTTTTTTGTATGAGGGTCCATCATATTGGTTTGCCATTAGGCCCTCACGTGACGTACGGTTGTTG
CACTATATCATTAAATATGATTGATGTGTCGTCGCAACGCATATGTATTGTATTAATTCTACAACATAAAGCAC
CGGTGTTTCTTATTTCCACCGTTCCACAGTCGCTCTGTGTCACCCTTTCTACATAAGGTAAAATTGTACATTA
GTGTGGGTTTGAGCATTGATTGTTGGATGTACATTCGCACCCACTTAACCTGTAGACCACATATTTTTGTTAC
CCTATAGATATATTCCCTTCCCCTACCACTGAGTTGTTACTTACCCTATAGATAGATCCTGAAAACTACTCAAA
CTCTTATTAGTGGATCGTTACCTCACAGATAAGCCGTAGAAAACCTTCCAAGCCCAACACGACAGGAAATTA
GATTTAACGTATTGCTCTCACATAGTATCATCTCGCTAGGTAAAGTAAGACAAACAATCGAAATAGTTATAAAA
AAAGGTGTCGAACTCATTTTTAATCCATAACTTACTAGCGCGTATGTGTACAAATTAGCTCAAGGAAAACGGA
ACTATGTAATTCGCCAAGGATTTATTTTTGGATATATAATATAAATAGGCTGACGGCTTGTTTCGTGACAACCT
GTTTCCGGTACAGAGTTAGCAGCCCACACCTGGTTTGTTCCATCCCACTACTATTCCAATCGTGTCGTCCAG
TGGTTGAGAGGACGGCACAAATGGAACCCTAGCTAATACGGAGTAGTATCTTATTAGTCCGTTTCAGACTGG
AGCTGCTATATATATAACGAGCTCGCCACTTGCGGTCTCGGCCTCGAA

IDE2 element (+ or – strand)

E-box (+ or – strand)

14. ZmFER1

GGGCCAATCTGGTTCAGAGCGCGCGTGAGGTCGCCACGCGCTAGTGAGCTGAGTCATCAGTTGCTAGCGA
GATCTGAAGGTACAGCCAACAACCATTCAGACGGGTGCGGTGGTCCTTCACTCCAACAAACAGCCTTGGAA
TTCTATGTGTGTCCTGTCGCAACCACGAATCTTCATCTTATCTCCTCCATCCTTGCTCGGGTCATCATCAGAG
GCGTGTGCCGGCCGCACGACCACTTTTTTTATTTTATTTTATTTTAAATATACGTCAGGTTTGTTGAAATAATA
ACGTGCTGCCGTTAATTAGATGAACTGGACGATATAAATTCCAACACCTACAAGTCTCGTGGTTATCCTTTGC
TGCTCTGCATTTACATTGAATACTAGGGGTCTGTTTAGTTTAGCTTCCTAGACCGGATTCGTTGTAAAAAATC
TGAATTAACTATACAACAGAATAGATTTTTCATATTCATCTCTTCCATCTTACAAAAATGTCACGTTCTTATAAA
ATCTATAGTTGACAGTTATTTCAATCAAACAAAATAAAGATTTTCCACAATTGTTAATGTTCGAGGAGATTTTC
AACATTCTCAACCAAAGGGGTGGTGTCTGTATGCACCACTTCTCCACCACCAAATTTAAAGCCATTCAAAAAA
AATTATGTCTGTGACTGCACAAAGTTTGGTGAAATTGATGAAACGGGCCATCCGAAGCTTCGGAAACTATGA
AACTGAAGTTGCAAACTTTAAAACATTTTGATAAGTCATTTTTATTTAAATTATTTTTAAAAACTATTTAAATTTA
TATTATACACCACGGCTTCACGTCAAAACTAGAACCTAGAACAATGCCAGACTCCTTATGGTATCCCTGCCTC
ATGGGAAAATAAGTTCTAGGACAAGGGGAAATTTGAGTTTATTTAGGGCTAGTTTGGGAACACTAATGTTTCA
TGAGATTTTCATTTTTCCAAGGGAAATTAATTTATTTTCCATTGGGAAAAT

IDE2 element (+ or – strand)

E-box (+ or – strand)

15. **ZmFER2**

A. **T001**

AATGGCGGATGGTTTCGAGATAGCTATCAAGAAACTGACGGAATGGCTTGCAAAGAACATTGACAAGAACAA
GACTAGGATATTTTTCGCAGGATCATCACCAACACATTCCTGGTAATATATATAAGATCGAGATGTGGTGTTG
TTGTTGTTGCTTTAATTAATTAATTAATTAATTTCAGGGCTAGCAACTGGGGCGGACAAGACAAGAACAAGTG
CCTGAACGAAACGGAGCCGATCAGCTACAGACCCGGCGGCGGGTACAAGGCTGCAACCACGGACTACAGC
CTGATGGCCATGGCCAGGTCCTACTTCCGGCGGACGCTGGAGCCGAGAGGCATACGCGTCCAGATACTCA
ACATCACGGAGCTGTCCGACTACCGCAAGGACGGGCATCCCACGGTGTTCAGGAGGCAGTTCGTTCCCCT
GACCAAGGAGCAGATCGCGGACCCGGCCAGCTACGCGGACTGCACGCACTGGTGCCTCCCAGGCGTCCC
CGACGTCTGGAACGAGTTCCTGTATGGCTACCTCACGCAGCAGAGCAAATGACGATGCATTATTACAAGAAT
GGCCTTGTTATATGTAGTAGTGTACAGTGCAGTACAGATTGCCAGCTGCTCCGTTTAGATTATTAGGGATGG
ACCTGCTGTTACATGTAGAGAGAGACAGACAGGAGATCTGTCTTGCCCAACATTAATTCTAGTATCTGTAAAT
CTTGTTCTTCTTGTCCAACATTAATTCTAGGATCTGCTGCTAACCATTTTTTTTGGATTTATGTAAACTGAGTG
ATTATCTGAAATAGAATGATCTGAGTTTGCTGTTCCAGTACTGAGCAAGTAAAAAAACCAAAAAAACAAATCG
CCCACCGTGGGGCTCGAACCCACGACCACAAGGTTAAGAGCCTTGCGCTCTACCGACTGAGCTAGACGGG
CTTGTTTTCCCTTTTTTTTTCTTTGGCTATAAATTGGATCCGCATCAGTCAGCTCAACACTTGTTACGGA

IDE2 element (+ or – strand)

E-box (+ or – strand)

B. **T002**

CCAGGTCCTACTTCCGGCGGACGCTGGAGCCGAGAGGCATACGCGTCCAGATACTCAACATCACGGAGCT
GTCCGACTACCGCAAGGACGGGCATCCCACGGTGTTCAGGAGGCAGTTCGTTCCCCTGACCAAGGAGCAG
ATCGCGGACCCGGCCAGCTACGCGGACTGCACGCACTGGTGCCTCCCAGGCGTCCCCGACGTCTGGAAC
GAGTTCCTGTATGGCTACCTCACGCAGCAGAGCAAATGACGATGCATTATTACAAGAATGGCCTTGTTATAT
GTAGTAGTGTACAGTGCAGTACAGATTGCCAGCTGCTCCGTTTAGATTATTAGGGATGGACCTGCTGTTACA
TGTAGAGAGAGACAGACAGGAGATCTGTCTTGCCCAACATTAATTCTAGTATCTGTAAATCTTGTTCTTCTTG

TCCAACATTAATTCTAGGATCTGCTGCTAACCATTTTTTTTGGATTTATGTAAACTGAGTGATTATCTGAAATA
GAATGATCTGAGTTTGCTGTTCCAGTACTGAGCAAGTAAAAAAACCAAAAAAACAAATCGCCCACCGTGGGG
CTCGAACCCACGACCACAAGGTTAAGAGCCTTGCGCTCTACCGACTGAGCTAGACGGGCTTGTTTTCCCTTT
TTTTTTCTTTGGCTATAAATTGGATCCGCATCAGTCAGCTCAACACTTGTTACGGAGCAACTAAACATAGAGA
GAGAGAGAGAGGGTGGATGACGAGGACGGCCTACTGCTTTCTTTGAGACAGCATGCATGCATGGCCTGAG
CTTGCTTTGAGCCCTCCTCGCTCGCTCGCTCACCTTCTTATCTCCTCCATCCATCCAAAAATATCTCCATGCG
CTCAGACGGGGACGAGACGACGGGAGAAGGAGGAGGAGGGCGCCCGCCCGCCACGCGCCTCCTCCAAAT
CCCAGCCGTCCACGTGTCCCAGTCCGCGACCCCCACGCGCCTATATCCATCCATCATCCTATTCCTATCCC
G

IDE1 element (+ or – strand)

IDE2 element (+ or – strand)

E-box (+ or – strand)

Core promoter sequences used (Maize Genome Database)

ZmNAS1

GGAGAATGATAGTCCAATGAAATTTGTTAGGGTGAGTGGAGTTCGTTCGTTGCATATTCAAACATACATTCTA
GTTACATAATAAAAAATTGACGTTTTGTTCTCTTATATGTCTTATATGTAGGATACAAAGATTCTTCGGATAAAC
TTTATTATCGAACTGTTAAGTTACGAGGAAAATTCGTGCAGACATGCCATCCCAGCAAATATACAACAGAGAG
TTAACAATATTGTTAAAAAATATTAGATTAGTGTACAAGGTGTCAACATATTTACTTATGATTAAGCATTATATA
TTTACTCTTTAGAAATTATTTATGTGATTCTATTTCAATCTATTTTGGTAGACATATTTATATCGTTCTGTATCTA
TGTTTAATAATCACATATTATACCGTCACTATTGTACTCACGCCGCACGTCGTCAGGGCCCTAAATCCATTGG
AGGGTCGCAATTTGTGTTCCGTGGCATCGCATGGTAACGTTCCTAGTTATATATTGATAGCTGCAAGACGTA
CATCATGTCAACTTTAGAGAGAGAAAGACAAGAGATTGGTTTGCTAGCTCTTCCCTTTATTGTGATGATTGAG
GCCGGGAGCAGTTTTCTTAATTAAGATTTTTGAAAGAACTTGTATTTTGTTTTTAATCTTTTTATCAGTGCTCG
ACCAATGTCATGATGTGGACGTGGCAAACCCACTTCGGGAGGGGACAAAAAAAACAGGGCTGCCACTTTCA
GGGCGTGACGTGGACGTGTTGAAGATCCGGAACATTCTTCGTGAAACGTACACCGTCAAAATAGCGAGGCA
TGAAACTGGCCTTGGCCATGGACGCGTGAAGCGCGCCATGCGTTGGATATGTGGTCAATAAGTATATACAAT
ACAATGTTTAACAGAGCTGGTAGTACTGCTTCGGCACATTTTTGTCCACGCTTCATGAGAGACAAAAACACCT
GCACTTAAATTCACATGCTGCACTGAAGGCCCGATCACTGAGGAGCGAAC

ZmDMAS1

GGAGTGTTGGAATTTATACATATCCCCAGACTTTTGGTACTGTTGTATTTGCATTACTTCCAGCGATGCTTTCT
TAGGATTGTGGACCTATTGGTTCTTTAAACGTGGTGGCTGATTCGCTTCCTTCGACTGAGCTCGCATCTTCG
GTACGTTGTTTTCTTCTGACCTCGATGGTTCCCAATTTGGTTTCAGCTTACACGCGTCGAAGACCCGGGTAA
CTGTCAGGAAAACGAGTAGGATGAAAGCGAAGCTCGCGGTGCTTGGGCGCGAGCCGCCCGGCGTTACAAG
TTGCAATGCAACTCTGGTGGAGGCTATCGCAAGTCACTACCCATATGAGGCCACGTTCGTTTGATCCCAAAA
CCATAGGGATTAAGGGGGATTGGAAAGGATTAGAGAGGATTTTGACTCGTAGAGAATTTGATCCCCCTTCAA
TCCCTATGGATCAGCACAAAACGAACGGGGCCTGACGGAGGACGGGAGGAGGCTTGTTTGTCCTATAACG
GCGTCGCGGAGCCTCAGCCAGCATATGTTCTTGTTGGATGCCGGAGTAGCGGAGTGGGCGAATGGACTGC
ACGCTCTCATGCCATGCGGCACGCCTACGACTACGAGATGCACGGCGACGCTGACGCAGCGCGGGGCCC
GCGGGGACGGACGGCATGAACCCGTCCAGTGTCTCGGGTCTCAGAGCTACAAACAAAGCCGCATGGTGGC
ATGATCTGTGCCTGTCCCGGTGGTGAGTAGTGACGATCGCTGCCGCTTTTCCCCCATCGGCCGCCGCAGCA
TGACATCACATATCAGTGGACTGGAGCAGGCAACAGAAGATCTCGAACTCGAAGCGTGAAAACCACTGCTA
GAGCATTTCATGTTACAGCCCGCGAAAACGTAAAAAAAATCGAAGGAATCCGAGGAGAAAATCAGGCAAGTC

ATGGCTACCGGACCCCGAACGCTCCTCAGCTCCTTGGACGTCCAAAAAACAAACGCAAACTTTTGTTGTTTG
TAGG

ZmTOM1

TTGGATGGAGTAAACAACAACATCAGTTAGTGAAATCAAAAAAATATTATGCAGAGAGCAGAGACAATTAATA
AAAATCTTGAGATCTTTTTGATGGATAGTTTACGTATATATTGTTGTGAGCCGTCGCAAAACGTACTGACATTA
TATATTGTCCAATCGTCGAACCGACGACTGAAGGTTAATTGATAGTGATACTGGTGGCGCCCAGGCCGGCC
GGCCGTAGACTAGCATGCATGCATGCAGAGCTTGGTTAGTTTCCTGTGGTGTGACTTGGTCATCAATTAAGC
GAGAGGCAGCTATGCATATGTCATGTGTACATGCATGTGTGGGTGGAGCGATTGGCACATTTCACGTTGTCT
CCCTTGCTTCTTGCACTGTGCGTGCTCATTGTGCCAGGGTTTGACTCCGCATATATAAATAGCTTCCTCTCC
GTCAGCGCTGACACAGTACGTCTTACTAACCATATTATGATACTCAAATTACCTGCCCACCATCCCCTGCAAA
ACTAGCCACACATATATGTAAGTATGTGTATTTGTTTGCTGCAGCGCTTCTCCTTCAAGTACGTACGTGTCGC
CTGTGCCAATGGATAATTTGATTTGCCTCGGCATAAATATATACAAACTGTGTGGCCTAGCTAGCTTACCAAC
CGTCTTTTGTCCTTGTATACTATGACAACAAGTTGGTTATACTCCGCGCGCCTTGCATCAGGAAATCGACGTA
GTGCAAAAGCGGGCGGGAGGGCGGCGGCATGTGCATGCATGCCGCCCATCGGCGCGTACGCATATGCCA
CGCACGCTACACAGAGACGTGCGTACGTACGTAGAGCAGTCTCGCTCGTCCAGCTCGATCTACACAACATC
TGGCTGACTAGCTACATGCAGTGTGCATGTCGTCATTATTACTCACCTCGGTAGTAGCATTACAATGTATGTA
TGTATATATAACCCATGCAGGCAGCTACCACCCGGCCAAGAGCAGTATTCCAGGCCCAGGGT

ZmYS1

ACTTGCGTATTTGCTGTTGGCGGCTGCCGACCCGCTGCACGCCTGCACCTCCCTGGCTCCCTCCCCGGCTC
CCGCGCCGGTGCCTCTGCCTTACTGCGTGGGCCGCCTCCGTCGACCTCGTGCGCTCGCAGCTCGGCCTCG
TGCCATCGTCATCCGCGTGCGCGCCACCGCGTGGACCGCCTCCGTCGGCCTCGTTGTAATACTTGGCGGT
GTACACGTGGGACTAAAAGGAAAACCAGCAATAGTGACACATAGGGGTCAGGGGAACTAAAAGCACAACCC
AATGCCGACGAAGACAACGCATAATTTTCAGTGGCACCGTTAAGGAAAATTTATATATTTTAACGGCATTCTA
AAAGATCTCTTAAATTTATATGTCTAAAACAAATTAGAATGAAATTTATATGTCTAGACAAATTAGAATGACTTA
TAATCTAGAACAGGTACTACAAAAGTCGGTCCAGTGACTCCAGTAGGCTCTTCTGGACTAACGGTAATAGGA
CATGTTTTTTTATAGAATATACACAAGCATATTTGTTAACGAGCACTTAGCAGATATGAGTACTGGACTAACGA
TAATATGTCACGATTCCCCGATAAACTTCGTTCCAAAGAAGTCATAACCGTTTGGTTCACTAAATGTAACGTA
AATGATAATGATAACGGTTCACACTCGAATACCGGCGGTAACAAATTTGAATGAGACGATATCCATTTGTAGT
ATGATATCGATTACGATTGGACTTAAACAAACATGATTTAACGTTATCGGTTACCGATTACGTTACCAATACGT
GAACCAAACGGCACCTAACTTCCATCAATTTGTCCGTGAGATGAACTATATACAGGTATACAGACATGAAGG
CAAGAAAAATGCTGCACCGATCAAGTCGCTTTCCTTCACCATTCTCCCCCCTAACAACTCTCTCGACAGCTAA
TAATGCGACGACTTGGAAATGTCTTAGTATTTTTCTGCCACGGAAATAGTTAAGGGTTTG

ZmNAS3

GATCTCTGCTGTAGCGTACACTAGCGTGTATGAACTCTAGTGCAGGACGTATATGTTCCTTGGGAATAGCGT
AAAGTAGAGAAAGGTAGATAGATAGATAGATATTCTTGGGGATCGGAATAAGTATATTTGAAGTAAAGGAAAT
ATGAGGCAACGACGACATGCATGGAACTTGAATTATTGTTATATGGGGCATCGTAGCTATGCTATATTATCGT
GACAGAAATGCACGGCCGTTCCTGACTTTTGAGGGGCCCAGGACAAAATTATAAACAGAGGCCCCAACACC
ATAAAAACTATTTTTTTACTATTATATATATCAACTAATTATACTTGATGTACCACAGAAAGACTTGAGCAGTAA
AAAATAGAAGTAAGATTATTACCTCAAATATATTGTAGAGATCAACTTAAAAATGTCTTCTAACGTTTCTAAATG
CAAAATCATCAATGATGGCTTCAATATTGACATCATCTAATAATTTCTTCTCAATACATAAAGTTGCTAACCCG
TCTAACCTTTCTTGAGAAATTATAGACCTTAAATAATTCTTCAATAACTTAAGCTTTGAGAAGCTCCTTTAAGCT
CATGCCACAGTTATTGATATAGTAAATAATATCTGATAAGCAACAAATATATTGGGATATCAATCCATTCTCCT
CAGAAACTCAAAAATATATTAGTATAGTAAATAATATCTGTTTCTATCCTATATTCCTATTAGACTTTGTGTGCG
GTGGGGCCCCTGGGAGATGGGGGCCCAGATCGGCCGCCCCTCCCGCCCTGCCTCAGGCACGGCCCTGCA
GAAATGAAATGGAACTTCACTCGCCAGTGCAAGTTGTACGCGTGCTGTGGCGACTGGCGACGACGACGACT
GCGACAAGGACGCCGTTGCGTGTGTGGAGAAAACAACGCAGAGGGCTTGTGGCGGGCACGCGTCTACAAG
ACAGGGACGCCATTATTCCCCCACCACCATATATATATAGTGTACTATGCCTCG

ZmIRT1

GCCTTCGTGCTGTTCTTCCTCTCCAACCTGCTGCCCCGCCCACGGTCGCAGCCGCGAGCCGACCGATGCT
CGTCGACGAGGGTACGGGGCGAGTCGGTCATGCTCTTGGTCTTTCTCCGTGCGTGCTATCGAGGAGGACA
TGGTGGCACCTGCCACGCTTAGGTTATCTTGGCGATTAGGAGTCCAGGCCTGAGATATTGGACAATCAAGA
CCCGTAGCGTGGCAGCAGTCGGCATATGCTACGGAGTTGGTGGTGGTGTTGTGTTGCTTCGGCGGGTCCA
GGGCTGGGAAGACATTCGCATTTTCTCGCCCTTCTCGGATTGGCGCCTGGTGCGGGTGCCTCTCGGTTTGG
AGCTGCTCCGGCTGGGCTTCTTTCTCCACTTGCATGCTCTCTCCCTATATATCTAGCGGTATACTACCTATGT
CATCTCCAGATGACCAGGTCTTCGTCGTGTCCTTCTCCGGCAACGAACAGGTATGTCCGCATCTTTCCTTCC
CCTCCACTCCTGCTCTACGAAACCTTGGAAGTGGGAGGGCAAGGGATTGGGGTCTCTGATTTGCTGTTTAT
GTTAACCGTGCGTTCAAAAAAAATTACGTGAAAAGTAGAGACAATCAATAAAAAAACTTGAGATCTTTTTGTG
GATAATTTATGTGGTTATTGTTGTGAGCCGTCGCAACGCACAGGCTAGTATGGTATTAAAGACACAGATCGG
CCTTCACATGAAAAATAGTGTGGAAATATAAGTGAGTTCTTCGTCTCTTATAAGTGAATACCAGTTAACGCTA
CTGAATAATGTTAGGATGTGTCCTCTACACTAGCCGATTAACAGTAACCGGGGTAGGGTTAATCCTAACCGT
CTAACCGCGCCCCTGATCGGGGGGCTATCCTAACCGTTGGTTGCAGCCCCCGGTCACACGACGCCATAAC
GTGTGCTGGAAGTGAAAGACCATCCAAGTTGCTCCAGGCCCTATATATATATGTGCAACCCCTACAACAGCC
G

ZmIRT2

ACATTAAACACTCGTTCATTCCTTAGACGTTGATCAATGCACATGGACTCCTACACTTGTGATAATTGCATACT
GCAGCACGAAGAGGCGATACTCCATCTATTCCTTAGATGCAATTTTGCTAGAAGATGTTGGGTTATAATATGT
ATCACCCCGTCAAGCACAGCTGATCTAGTACATGCTTTATTGAGGATCAGAGTGAGATCGAAAGTACCATGG
AGAATGGAAATTATCATAATCATGTCCTGGTGTATTTGGAGGAGTAGGAATAACTGGATATTCAACGAGATCC
CGACGTAGGTGGAAACGTGCAGGGAGATGTTCAAGAGTGAAATGAGACTCATTTGCCACAGGATTAAGTCA
GAGGTAGACGATAGAATTAGAAGTTGGATACAACACTCTATAACATAGTGGTATGCTACTCCATCTTGTAAAT
TTTACATACTTATCCCAGAAACAATAAAAATATTGTAGGCACATCCTACAGTAACTTACTATGGAAAAAACACG
CTCTAACGTATTGGATCCAAACTTAGCTAAGGGCATCCTTGTCAGAAATATACAGGTAACACGTCATATGCTT
TTTAGAAAAGAAAAAAAAGGCACTGATTCTACGGACGATCGAATCCAATGGCGCATCTGTTTTATGTTAGCTA
AAGTTGTTGGAAACGGCAGAAAATAGTAAAACAAAATGAAAAACAAGCCGTATGTAATCGGCAATAAAATGA
GCGGCATGTCTGTGTCGGGCGCCCACCGTATCCCTCTGCACCAGCTCCGCGCGTGCCCCGCTCGATCCCA
GGAAGTACGGAGCTGTACGGCACGCGACAGGGTACGGAGGCGCGCGTGGCCTCCCCGTTCCCGCCTCGT
GGCCTGCAAGGTCAATGGCGCCCGGATTGATCGACGGCCGAGCTAGTCTTGCATCATGATGTGCGCTCCAC
AATGGACATGCAACTGCTCCTCCTATAAAAAGGCCTGCAGAAGCTCATCGCAAGTCCACATCC

ZmIDEF1.1

TCCATTGCTCCTGCCCCTAGAATTGAAGGTTGGTTCATGTTTGGTGGAAGACTAAAAAGGACAAAGGAAGGA
GGCGTTGTATCAGTAGCACCGTTGCCTAGTTACATTAGCATCACCATCAGCATCAACCAAACAGTACAATAG
CTGCATTTCACAAACATATTATCGGAATACTTAATGGTCCTAGTTTTACAATACTTGCCTAGATTATTAGAATC
CTCGGATCTGTACTTTACCGGACGGTCACCAAAATACTTAGCTTAACATACTAGTAAAATATGAAATTCTTCTA
ACGATTTTTAGAATGCATACTACCCTCCACTTTAGTTTACGTATCATTGGTAGAGAACATTGCAGCAATTCTGA
CGATGAGTTTTTTTTCTTTCTAGAAAAAAAAGATTAAATTTGTTGAGACTTATGTTTGAGTAGGTGTATATAATA
CACTTCATTAGCATTGTATACTTTTAGATTAAAAAATGGGAGTGTACGCAACTTCCTAATGACAAATACAATAC
TTCATTTATTATGAATAAAAAATTGGATCCGAATGACTAGTCACTACATTTTTAATGGGGTATTTGCCGACTAA
ATTCTAATGTTTAAATGATAATTATTGGTGCTAAAAATAGTTTAATTATTATTAAATAATATGATGTAATTGAAG
GCGTGCGCGGCAGAATGGATAGTGATAATGGGTGCAAATCCTGGGAGGATATTTTTGTTCCTTGCCTTTACC
CTTTTTTTCCCCTCTCACGGTCGAATCGAAGATACGAAGAAGTCGGAGGAGGAAAAAAAAGCAAGGAATTCA
CCATCAGTACGTTCTTGCGGCGTGTAGAGAGAGAAGAGAACGGGTGACAGTAGCAAGCAGCCATAGCATAG
CAGGACCACTCCTGCGCTGCACTGCACCAGTCTGCAGGTGGGTGCGGTGGGGGTGGCCGGGTGGGGTGA
AGTGGAGAGAGAAAAGAGGAGGAGGTCGTCTGGCCCTTTCCCCTTCTTTGCCC

ZmIDEF2.1

GTAAATTACAATAACATACAAGAAATTTCCCCGTATCTAATCCATTCACTCCCATGAAAATATTAGGTCATTTT
CAATAGAGAGTTTTATCGCACTGTTTTTAAAACTGCCATGTCAATATTTGTCTAGGAAATAATGTATCTAGAAT
TTCATCCCATGAAATTATTTGATCTCTCATGAAACTCTCTCACATCTCTTTTTATTAAATTGCATGCCATGCCAT
CCTATTTACTTATATGGTATGCCATTTAATGAGAGTGAAACTCCAACTGACAATGATTTTACCATACTTCTTGT
CAACCAATCAGAGCAGCTCATTCATGGTTATCACATGACCAAGCTTGAATGCTATTTCTCCACTAATTTTTTGA
GCTCTTGGACTTTACACCAAATCAACTTTGTCGAGTTCACTATGATTTTTTTCGGCTCTAAATATTTAGATATC
ATTCAACGCTATTCATTGATGAAAAATATATGAACATGCACATATACTATGTGATTTCTAAAAAAGTAGACTCC
ATAGATAGTATCATAGTATGCTCCCAAGTCCCAACCATTAGACTTTAACCACCATTATTCTCATACCTTAAATG
TAGGCTTGGATTGAGATCCAAGCAATTACCAAACCAATTTTTTTATCCTCGACTACCCTACCATCACATTGCA
AGTGGAAATTTAATGTTGTCTTCAATGGTATCTTTTATATTTGTCGTTTAGAGATTTTCTAAAAGTTTTTTCTCT
ATATTTTATTTCTCTTTAACGATATTCTATATTCACATCCTTTGGACATTAAAGATCTATATTAAAACATATTTTA
CTTGAAAACTAGAGAAACCTTTATCGTGTCTGGTTAGAGTCACGGCCGACTCCGTGGAAATTTGGAAACCAC
GGTCACGTACTGCTGGGACTGGGAAGCTTCCTGTCCAGAATGCTGCGGCCTGCGGGAGTTGGAAGCAAAG
AAATAGAAAAACCCATTGGTCCCGCTCGCCAGTGTCCCGGTA

ZmIRO2.1

TGTGGATGCGGCGCGGCCGCGGCAGAACGTGCGGCGCGGCGCGTGCGCGGCTGGGGCGGCTACGGGAT
GTGGATGCGATGCGGCTGCGGGCGCGGCGCGTGCGCGGATGGGGCGGCTGCGGGCGTATGCGGGATGC
GGAGCGTCGCGGCTGCGGGCGCGGTAGAACATGCGCCAAGCGTTAGTTGGACGCCTACATTACTAACATA
ATTAGTAGTAGAGATTCACTACCCCTCTGTGTCACCGTTAACCGTTTCCTACAACTCCAGTGAAGCAGTGAC
GACCTAGCAGAGCGGCGCTGGTAACATGTGGGCTGTGGCCCCCGTGGCTGGACAGCACGGGGAGAAGGG
CTGTCGGGCACACGCCGGCACGAGGCGCTCCCGTTCCTCCACGCTCGCCTAGGGTGTCGACACCCTCACG
CGACGTACTAGTACTCTACGTGTCTCGATTAGTATTAAATAATGCCTCCGGGTTTTAGTATATTAGACAATAAT
AGTATAGTTACAGGCGCCCTGATCACACACTTCAATTCTCACAGCTAGCCAGACGCTGAGCTAGGCTGCATG
GATTAATATGCATCTTCCCTCAACTCTTCTCATTAGCCCTGACGTCATATCACCGTTTTATTTAGCTTAATGGG
GTTTTATGGACATTAAATAATCTAACACGACATAGGATTTTATGAGATGGAATGAGTCCTTGTCCATTCAGTAC
GGTTTTGTAGACGACTCGTATTTTCTTTCCTTTAAAAAAACATTGCTGCACCATCAAAGGACGGTTATGTAGC
AGCTTATTATTAAGTGTAAATAAAACTTGTCCTCCTACATTGGGGAGTTTAGAAAAGCTAGGAACATGAGATG
ACATCCTATACTGTAGGCTTGTTGCTTTCGTACATACTGAAACTAGACCAACCTTTCGTATAGCTCTAGGTAC
TCGACCACCAGAGATTTTTATTATATATAGTTAACAAACATTGCCTGCTGCTAATCATCTATCCATAAAAT

ZmIRO2.2

ATATAGCATGCACACACTAAAAAATATGTATGTTGGCAGAATTATTAGTCAAGAATTTAAAGAGTTGACTTTTT
TAAAAAAAATTGGGATTGAGGGAGTAATATTTTATATACAAAGTGTATGTATGGTTATTTTCGGTAATCTTTTTT
TTTAATCATATGAGCTAGCGCTTTAAAAGGACCATTACAAACACCCCAAGTATCTTTGAATGAATAATAAAAAT
ATATTTTGACATATAAATATTATGTACTGTTTTTTATAATCTTAGTTAAACTTTAAAAACTTCACGTTACTTTAAA
CCTAAAATGTCATGTTTTTTGAACAGACGGAGTATGGCTCTAGGCCGGCCTGATTATCAGTTCTTGCTTTAGG
GCATGTTTGGATGAGCTAAAGCAAAAGTGAGTAAAGTTTAGTCATTTAGAAAATAAAGATCCAAACAGAAAGA
GTTGAAAAAGTTAAAAGTGACTAACTTGCCCTTAATTAGTCACCTCAATCTCAATCCAAACATGCTCCTTTAG
GGCTTGTTCGGTACTAGCCCAATCTATATGAATTAAGGACTAGTTTTGATACTTTGGGATTAGAGTGTTTTGG
AGGGATTGGAGAGGGTATAAATCCCTAATAGATCAAATACTCTTTCAATACATCTCAATCCACTCTAATCTCA
CTCATTACTAGAGTCCCCAAACTAGGCCTAAGGGGGATTGAGGGAGTTTCAATCCCTAGTAAGTCAAAACCT
CTCCGGTCCATATCAGTCCACTTCAATTGATATGGATTGAAAATAACCCAGCATGCTCTTACCGAAAATTCTT
GGCCGGCCAACTCTAGACCATGCATCCTGGCCTGTCGTATTGTAAAAGCTAGCAGGTACAGTACTTTTTACT
GCATGTGATAATGTAGGCAGTTCACAGTCTCTGTGGGACATAGTGACGACACCTACCCGTGTCCACCATCTC
AACCCTAGCTATATAAATAGTAGGGAACAAGTCGCCTCTTTGAAACTG

ZmIRO3

AAAGAGATGACAAAAGATATGAGGAAACAAGTGTACCAAGCGTTGTTGGCTAGAAGCAATAACGGGAGACTC
CACAAGAAAGATACCCAAATTGTTGCCAACCAATTTGATCTCCACCTTCGGTCAGTGCAGCGCGTATGGAAC
AGAGGTAAAATGCAACTTGCAAACTCGGTGCCGGTTGTGGTTGCTAGTCTAAAAAAGGGAAGAGTTGGTCGT
AAAGCAATTCCTCTGCTGCTGAGTCCGGCACGAGCTCGAAGGAGTCCAGCGTGAGCTCGAAGGAGTCCGG

CGCGACCTCGAAGGAGTCCAGCACGAGCTCGAAGGAGTCCAACGTGGATTCTGAGTCAGACGCGACGGAG
TCCAGTGACATCTCGGAGTCCAGCGACATCTCGGAGTCCAGCGAGGACGAGACGTCATCCTTGGCCACGG
CGAGCGCAGAGTCGGAGTCCATGGCGAGGCGCGAGCAAGGAAAGCGAATGCGAGACGCGAGCAGAGTCG
TGCGGTGTTTGCATGTCAGGCGCGCGGTGTTTGCATGTTTGGCGTGGGCGTTAATGCATCACGTTGGTTTC
CTTGCATCGTGCGGTGTTTGCATGCGAGACGGAGTAATAATGCATGGCGTGCGGTGTTAATGCATGGCAAC
GGGCGACGCAGAAAACGAAGCAACGGGCGACGCAATTACTCAGGGGCAGTGACGTCCAAATTCTAGACCC
ATGCGTGCTACGATTTCTTTTGTGCACGTTCGGTCACACGCAGAATGACTTCCTTTACTGTACCGGAGGGAG
TACATGTGATCTCCGGGCATGTGGTTTATTTTACTCTGGTCTCTGGGACTCTCATGATGATGATCATGTCAGC
GGTGCCAACGATTGTGGATCGCGATGACACGACAGCGTAGGAGATGTTGATGGGCTGAGTCGGACGAGAC
GCCAGCAGGATGGCCCTTGGCGCGAGCCAGATGCCAACAGCCCTGTGGATGTGGGTGGCAGCGTGGAGG
CTCGTGGCCG

ZmFIT2

AAGAAAGATAGTCGGTCGAGTGAGCTATAAAAAGAGGTGTGTTTTATTTTCAACTCATAATTTTCTGCAGTTTA
ATATCTCGTACGTGGGTCATATATCTACTTTAATTTTAAATTTATTTTTTATGGGGATGGTCCACTTTAATTTTA
AATTTATATTTTTTTGTATGAGGGTCCATCATATTGGTTTGCCATTAGGCCCTCACGTGACGTACGGTTGTTG
CACTATATCATTAAATATGATTGATGTGTCGTCGCAACGCATATGTATTGTATTAATTCTACAACATAAAGCAC
CGGTGTTTCTTATTTCCACCGTTCCACAGTCGCTCTGTGTCACCCTTTCTACATAAGGTAAAATTGTACATTA
GTGTGGGTTTGAGCATTGATTGTTGGATGTACATTCGCACCCACTTAACCTGTAGACCACATATTTTTGTTAC
CCTATAGATATATTCCCTTCCCCTACCACTGAGTTGTTACTTACCCTATAGATAGATCCTGAAAACTACTCAAA
CTCTTATTAGTGGATCGTTACCTCACAGATAAGCCGTAGAAAACCTTCCAAGCCCAACACGACAGGAAATTA
GATTTAACGTATTGCTCTCACATAGTATCATCTCGCTAGGTAAAGTAAGACAAACAATCGAAATAGTTATAAAA
AAAGGTGTCGAACTCATTTTTAATCCATAACTTACTAGCGCGTATGTGTACAAATTAGCTCAAGGAAAACGGA
ACTATGTAATTCGCCAAGGATTTATTTTTGGATATATAATATAAATAGGCTGACGGCTTGTTTCGTGACAACCT
GTTTCCGGTACAGAGTTAGCAGCCCACACCTGGTTTGTTCCATCCCACTACTATTCCAATCGTGTCGTCCAG
TGGTTGAGAGGACGGCACAAATGGAACCCTAGCTAATACGGAGTAGTATCTTATTAGTCCGTTTCAGACTGG
AGCTGCTATATATATAACGAGCTCGCCACTTGCGGTCTCGGCCTCGAA

ZmFER1

GGGCCAATCTGGTTCAGAGCGCGCGTGAGGTCGCCACGCGCTAGTGAGCTGAGTCATCAGTTGCTAGCGA
GATCTGAAGGTACAGCCAACAACCATTCAGACGGGTGCGGTGGTCCTTCACTCCAACAAACAGCCTTGGAA
TTCTATGTGTGTCCTGTCGCAACCACGAATCTTCATCTTATCTCCTCCATCCTTGCTCGGGTCATCATCAGAG
GCGTGTGCCGGCCGCACGACCACTTTTTTTATTTTATTTTATTTTAAATATACGTCAGGTTTGTTGAAATAATA
ACGTGCTGCCGTTAATTAGATGAACTGGACGATATAAATTCCAACACCTACAAGTCTCGTGGTTATCCTTTGC
TGCTCTGCATTTACATTGAATACTAGGGGTCTGTTTAGTTTAGCTTCCTAGACCGGATTCGTTGTAAAAAATC
TGAATTAACTATACAACAGAATAGATTTTTCATATTCATCTCTTCCATCTTACAAAAATGTCACGTTCTTATAAA
ATCTATAGTTGACAGTTATTTCAATCAAACAAAATAAAGATTTTCCACAATTGTTAATGTTCGAGGAGATTTTC
AACATTCTCAACCAAAGGGGTGGTGTCTGTATGCACCACTTCTCCACCACCAAATTTAAAGCCATTCAAAAAA
AATTATGTCTGTGACTGCACAAAGTTTGGTGAAATTGATGAAACGGGCCATCCGAAGCTTCGGAAACTATGA
AACTGAAGTTGCAAACTTTAAAACATTTTGATAAGTCATTTTTATTTAAATTATTTTTAAAAACTATTTAAATTTA
TATTATACACCACGGCTTCACGTCAAAACTAGAACCTAGAACAATGCCAGACTCCTTATGGTATCCCTGCCTC
ATGGGAAAATAAGTTCTAGGACAAGGGGAAATTTGAGTTTATTTAGGGCTAGTTTGGGAACACTAATGTTTCA
TGAGATTTTCATTTTTCCAAGGGAAATTAATTTATTTTCCATTGGGAAAAT

ZmFER2

A. T001

AATGGCGGATGGTTTCGAGATAGCTATCAAGAAACTGACGGAATGGCTTGCAAAGAACATTGACAAGAACAA
GACTAGGATATTTTTCGCAGGATCATCACCAACACATTCCTGGTAATATATATAAGATCGAGATGTGGTGTTG
TTGTTGTTGCTTTAATTAATTAATTAATTAATTTCAGGGCTAGCAACTGGGGCGGACAAGACAAGAACAAGTG
CCTGAACGAAACGGAGCCGATCAGCTACAGACCCGGCGGCGGGTACAAGGCTGCAACCACGGACTACAGC
CTGATGGCCATGGCCAGGTCCTACTTCCGGCGGACGCTGGAGCCGAGAGGCATACGCGTCCAGATACTCA

ACATCACGGAGCTGTCCGACTACCGCAAGGACGGGCATCCCACGGTGTTCAGGAGGCAGTTCGTTCCCCT
GACCAAGGAGCAGATCGCGGACCCGGCCAGCTACGCGGACTGCACGCACTGGTGCCTCCCAGGCGTCCC
CGACGTCTGGAACGAGTTCCTGTATGGCTACCTCACGCAGCAGAGCAAATGACGATGCATTATTACAAGAAT
GGCCTTGTTATATGTAGTAGTGTACAGTGCAGTACAGATTGCCAGCTGCTCCGTTTAGATTATTAGGGATGG
ACCTGCTGTTACATGTAGAGAGAGACAGACAGGAGATCTGTCTTGCCCAACATTAATTCTAGTATCTGTAAAT
CTTGTTCTTCTTGTCCAACATTAATTCTAGGATCTGCTGCTAACCATTTTTTTTGGATTTATGTAAACTGAGTG
ATTATCTGAAATAGAATGATCTGAGTTTGCTGTTCCAGTACTGAGCAAGTAAAAAAACCAAAAAAACAAATCG
CCCACCGTGGGGCTCGAACCCACGACCACAAGGTTAAGAGCCTTGCGCTCTACCGACTGAGCTAGACGGG
CTTGTTTTCCCTTTTTTTTTCTTTGGCTATAAATTGGATCCGCATCAGTCAGCTCAACACTTGTTACGGA

B. T002

CCAGGTCCTACTTCCGGCGGACGCTGGAGCCGAGAGGCATACGCGTCCAGATACTCAACATCACGGAGCT
GTCCGACTACCGCAAGGACGGGCATCCCACGGTGTTCAGGAGGCAGTTCGTTCCCCTGACCAAGGAGCAG
ATCGCGGACCCGGCCAGCTACGCGGACTGCACGCACTGGTGCCTCCCAGGCGTCCCCGACGTCTGGAAC
GAGTTCCTGTATGGCTACCTCACGCAGCAGAGCAAATGACGATGCATTATTACAAGAATGGCCTTGTTATAT
GTAGTAGTGTACAGTGCAGTACAGATTGCCAGCTGCTCCGTTTAGATTATTAGGGATGGACCTGCTGTTACA
TGTAGAGAGAGACAGACAGGAGATCTGTCTTGCCCAACATTAATTCTAGTATCTGTAAATCTTGTTCTTCTTG
TCCAACATTAATTCTAGGATCTGCTGCTAACCATTTTTTTTGGATTTATGTAAACTGAGTGATTATCTGAAATA
GAATGATCTGAGTTTGCTGTTCCAGTACTGAGCAAGTAAAAAAACCAAAAAAACAAATCGCCCACCGTGGGG
CTCGAACCCACGACCACAAGGTTAAGAGCCTTGCGCTCTACCGACTGAGCTAGACGGGCTTGTTTTCCCTTT
TTTTTTCTTTGGCTATAAATTGGATCCGCATCAGTCAGCTCAACACTTGTTACGGAGCAACTAAACATAGAGA
GAGAGAGAGAGGGTGGATGACGAGGACGGCCTACTGCTTTCTTTGAGACAGCATGCATGCATGGCCTGAG
CTTGCTTTGAGCCCTCCTCGCTCGCTCGCTCACCTTCTTATCTCCTCCATCCATCCAAAAATATCTCCATGCG
CTCAGACGGGGACGAGACGACGGGAGAAGGAGGAGGAGGGCGCCCGCCCGCCACGCGCCTCCTCCAAAT
CCCAGCCGTCCACGTGTCCCAGTCCGCGACCCCCACGCGCCTATATCCATCCATCATCCTATTCCTATCCC
G
